# Factors Influencing Adoption of Large Language Models in Health Care: Multicenter Cross-Sectional Mixed Methods Observational Study

**DOI:** 10.2196/84918

**Published:** 2025-12-11

**Authors:** Xiongwen Yang, Yi Xiao, Di Liu, Huiyin Deng, Jian Huang, Yubin Zhou, Maoli Liang, Longyan Dong, Zihao Yuan, Jing Yao, Wankai Guo, Chuan Xu

**Affiliations:** 1 Department of Thoracic Surgery, Guizhou Provincial People's Hospital, No. 83, Zhongshan East Road, Guiyang, Guizhou, 550000, China, 86 18620726507; 2 NHC Key Laboratory of Pulmonary Immunological Diseases, Guizhou Provincial People's Hospital, Guiyang, Guizhou, China, 1 18620726507; 3 Department of Cardio-Thoracic Surgery, Third Affiliated Hospital of Sun Yat-sen University, Guangzhou, Guangdong, China; 4 Department of Anesthesiology, Third Xiangya Hospital, Central South University, Changsha, Hunan, China; 5 Department of Thoracic Surgery, Jiangxi Provincial Cancer Hospital, Nanchang, Jiangxi, China; 6 Department of Dermatology, University of Hong Kong-Shenzhen Hospital, Shenzhen, Guangdong, Guizhou, China; 7 Department of Respiratory Medicine, Guizhou Provincial People's Hospital, Guiyang, Guizhou, China; 8 The Second Clinical Medical College, Guangdong Medical University, Dongguan, Guangdong, China

**Keywords:** large language models, artificial intelligence, digital health, health care professionals, patients, technology adoption, mixed-methods study

## Abstract

**Background:**

Large language models (LLMs) such as ChatGPT are transforming how health information is accessed, communicated, and applied. However, their adoption in health care remains limited by uncertainties surrounding trust, privacy, and digital readiness, particularly in low- and middle-income contexts.

**Objective:**

This study aimed to examine how trust, information behavior, and sociotechnical readiness influence the willingness of health care professionals (HCPs) and patients or caregivers (PCs) in China to adopt LLMs for medical information and decision support.

**Methods:**

We conducted a multicenter, cross-sectional mixed methods observational study across five tertiary hospitals, combining surveys of 240 HCPs and 480 PCs with semistructured interviews (n=30). Quantitative analyses included logistic regression (LR), random forest (RF), and extreme gradient boosting models with Shapley additive explanations-based interpretability. Qualitative data were thematically analyzed to capture role-specific concerns and expectations.

**Results:**

Among HCPs, mean age 39.9 (SD 6.5 years; 159/240, 66.2% physicians), 69.2% (166/240) were aware of LLMs and 36.7% (88/240) had previous experience. Among PCs (mean age 50.1, SD 12.6 years; 242/480, 50.4% male), only 26% (125/480) had previous exposure. Trust, perceived usefulness, and digital readiness were the strongest facilitators of adoption. Multivariable models identified trust as the dominant predictor for both groups (HCPs: odds ratio [OR] 3.78, 95% CI 2.15‐6.63; PCs: OR 36.34, 95% CI 18.41‐71.74; *P*<.001). For HCPs, previous use (OR 5.61, 95% CI 3.02‐10.44; *P*<.001) and legal clarity (OR 1.56, 95% CI 1.07‐2.27; *P*=.02) increased willingness, while privacy concerns reduced it (OR 0.72, 95% CI 0.53‐0.97; *P*=.03). Among PCs, perceived usefulness (OR 2.01, 95% CI 1.52‐2.67; *P*<.001), education, and digital tool use were positive predictors. Model performance was high (area under the receiver operating characteristic curve [AUC] 0.83‐0.85 for HCPs and 0.93‐0.96 for PCs). Qualitative findings identified 11 themes: HCPs stressed workflow integration and accountability, while PCs emphasized comprehensibility, reassurance, and equitable access; trust consistently linked technical credibility with social legitimacy.

**Conclusions:**

Adoption of LLMs in health care depends less on algorithmic performance than on the management of trust, literacy, and institutional readiness. Trust functions as a multidimensional construct rooted in transparency, reliability, and contextual validation. Theoretically, this study extends technology adoption frameworks by embedding ethical trust, digital literacy, and institutional support within a unified sociotechnical readiness model, advancing information management theory beyond performance-centric paradigms. Empirically, trust and perceived usefulness outweighed demographic or structural factors, with predictive accuracy exceeding 0.9 across user groups. Practically, these findings offer actionable guidance for the design and governance of artificial intelligence systems, emphasizing role-sensitive interfaces, plain-language communication, and transparent accountability mechanisms to promote equitable and trustworthy adoption.

## Introduction

The rapid advancement of large language models (LLMs), such as ChatGPT (OpenAI) and other generative artificial intelligence (AI) tools, has opened new opportunities in the management and use of information across multiple sectors, including health care [1-4]. These models, trained on vast datasets of biomedical literature and clinical texts, are increasingly capable of performing tasks that fundamentally reshape the way information is accessed and applied, such as explaining medical concepts, summarizing patient records, supporting decision-making, and facilitating communication between patients and professionals [5-10]. Their accessibility, versatility, and potential for integration into organizational workflows highlight LLMs as a significant force in transforming information practices, with implications for efficiency, equity, and engagement [11].

In the field of information systems, the integration of LLMs into enterprise and knowledge management systems is shown to optimize data processing and automate business workflows, marking a new era of intelligent data capabilities [12]. Research in knowledge management has documented how generative AI enhances knowledge transfer, decision-making efficiency, and operational effectiveness through improved content creation and knowledge dissemination [13,14]. In the realm of information systems research, generative AI has been identified as a transformative technology with profound societal and organizational implications, calling for re-evaluation of research agendas and considering its evolving influence on business and social systems [15]. Additionally, LLMs are being deployed to support knowledge workers, streamlining complex tasks through natural language interfaces and enabling new kinds of workflow integration [16,17].

However, the integration of LLMs into sensitive domains such as health care raises critical questions for information management theory and practice. Concerns about accuracy, data privacy, transparency, embedded biases, and accountability are by no means resolved, reflecting broader challenges in governing AI-augmented information systems. Recent studies have shown that the trustworthiness, explainability, and ethical transparency of generative AI remain the decisive barriers for clinical and public adoption, especially when users lack institutional guidance or previous exposure to AI tools [18-21]. Information management research emphasizes that the governance of information artifacts in high-stakes environments must address these sociotechnical risks at multiple levels, from technology design to organizational oversight [22,23]. Importantly, successful adoption of LLMs depends not only on technical performance but also on stakeholder perceptions and acceptance.

Emerging literature now highlights trust, fairness, and data governance as critical enablers of responsible AI implementation in health and public services [24-27]. Understanding how health care professionals (HCPs) and patients or caregivers (PCs) assess usefulness, trustworthiness, and risk is essential, not only to ensure that LLMs enhance information use and knowledge exchange but also to prevent them from reinforcing existing inequalities or mistrust [28].

Previous studies in high- income contexts have begun to explore how clinicians interact with AI-generated information. However, limited evidence exists from low and middle-income countries, where factors like digital literacy, information trust, and institutional readiness critically shape technology adoption. A systematic scoping review highlights that AI initiatives in low- and middle-income countries’ health care, such as clinical decision support and chatbots, face persistent challenges, including reliability, user-friendliness, context misalignment, and distrust of AI systems [29]. Recent empirical evidence further emphasizes that digital readiness, cultural values, and governance frameworks substantially moderate AI acceptance across diverse health care environments, suggesting the need for region-specific analyses to guide equitable adoption [30-33].

Specifically in China, the socioeconomic diversity between urban and rural regions suggests that attitudes toward LLMs may vary significantly. Insights from information management research emphasize the digital divide in low- and middle-income countries, pointing to disparities not merely in access but in digital capacity, health literacy, and sociotechnical support systems [34,35]. Therefore, examining LLM adoption in China becomes an inquiry not just into technology implementation, but into information behavior and management within complex sociocultural and infrastructural contexts [36]. Given China’s unique ecosystem, where rapid digital transformation intersects with uneven regional literacy and regulatory evolution, understanding user trust and institutional readiness offers critical insights for both national policy and global information systems theory [37-40].

Against this backdrop, this study aims to systematically examine how trust, information behavior, and sociotechnical readiness influence the willingness of HCPs and PCs in China to adopt LLMs for medical information access and decision support. Specifically, we investigate the relative importance of psychological, informational, and institutional factors in shaping adoption intentions. We hypothesize that trust and digital readiness, rather than demographic attributes, serve as the strongest predictors of willingness to adopt LLMs. By using a multicenter mixed methods design, this study provides empirical evidence that bridges theory, governance, and real-world implementation, offering insights for both information management theory and responsible AI deployment in health care.

## Methods

### Reporting Standards

The study was reported in accordance with the Good Reporting of A Mixed Methods Study (GRAMMS) guideline [41], which provides a comprehensive framework for ensuring transparency, integration, and methodological rigor in mixed methods research (Multimedia Appendix 1). This approach was chosen to better reflect the interconnected design and analytical structure of the quantitative and qualitative components of this study.

### Study Design and Setting

We conducted a multicenter, cross-sectional mixed methods observational study across 5 tertiary hospitals located in 4 provinces of China—Guangdong, Hunan, Jiangxi, and Guizhou—between August 2024 and March 2025. The study was initiated and centrally designed by the research team at Guizhou Provincial People’s Hospital, with all other hospitals participating as independent data collection sites under a unified protocol. The participating institutions were the Third Affiliated Hospital of Sun Yat-sen University, the Third Xiangya Hospital of Central South University, the University of Hong Kong–Shenzhen Hospital, and Jiangxi Cancer Hospital.

These sites were purposively selected to ensure diversity in regional economic development, digital infrastructure, and patient population characteristics. Each hospital facilitated participant recruitment and on-site data collection through its own clinical departments, while all data management, integration, and analysis were performed by the coordinating research team at Guizhou Provincial People’s Hospital.

Data collection occurred in parallel across all sites to minimize temporal bias and included both quantitative surveys and qualitative interviews conducted on-site or through secure teleconferencing platforms.

### Participant Recruitment

Participants were recruited consecutively from outpatient and inpatient departments of the 5 hospitals. Health care professionals were invited through internal departmental briefings, institutional WeChat (Tencent) groups, and email lists, while PCs were approached in clinical waiting areas and wards by trained research assistants.

Eligibility criteria required health care professionals to be licensed physicians or registered nurses currently engaged in clinical duties, and patients or caregivers to be adults (≥18 years) receiving or accompanying medical care during the study period. Individuals unable to communicate in Mandarin or with cognitive impairment were excluded.

Recruitment followed a purposive-consecutive hybrid approach to ensure representation across different departments, hospital types, and regional economic levels. The final sample size (240 health care professionals and 480 patients or caregivers) was consistent with power analysis targets and provided sufficient heterogeneity for multivariate modeling and subgroup comparisons.

Data collection was conducted on-site using either paper-based or secure QR code–linked electronic questionnaires, according to local logistical capacity. Research assistants were trained to ensure standardized recruitment, eligibility screening, and data integrity across all centers.

### Assessments and Data Sources

**Figure 1.**
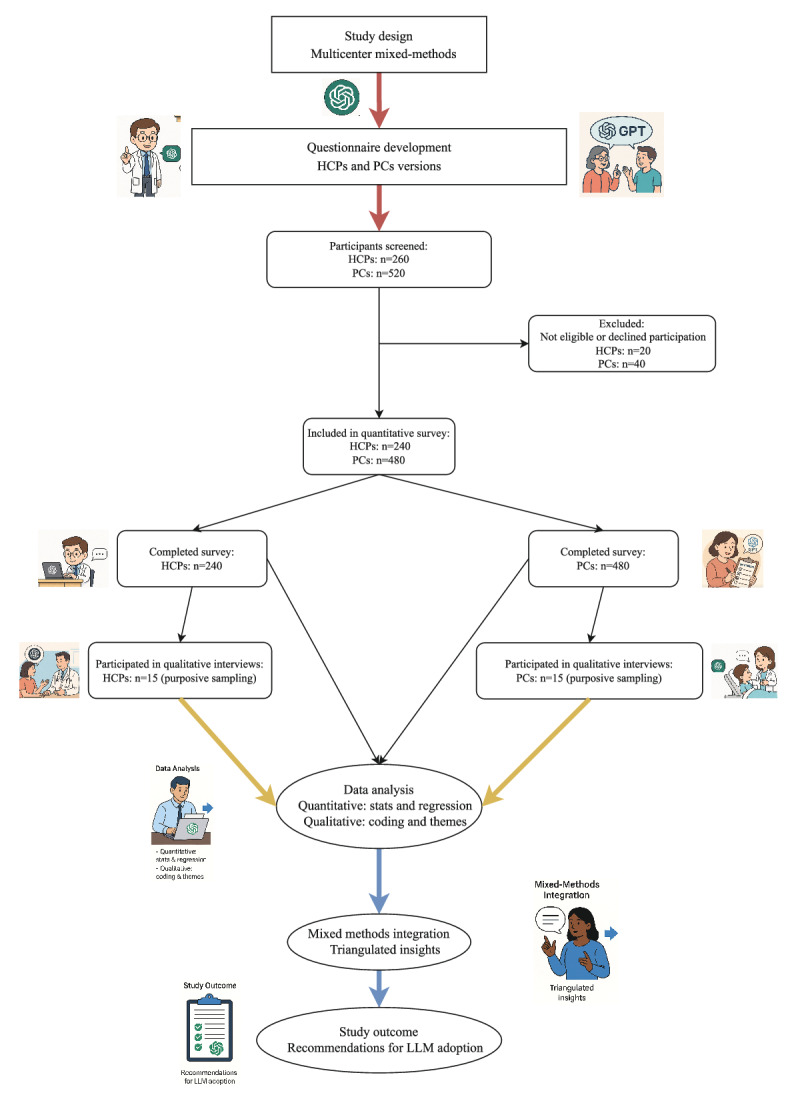
Overview of the study design, methods, and analytical framework.HCP: health care professional; LLM: large language models; PC: patients or caregivers.

The overall study flow is illustrated in Figure 1. In total, 2 structured questionnaires were developed separately for health care professionals and patients or caregivers (Tables S1 and S2 in Multimedia Appendix 2). Variables included demographic and contextual characteristics (age, gender, region, education, role, and clinical experience), digital tool use, previous exposure to AI or LLMs, and perceptions related to (1) perceived usefulness (expected benefits of LLMs for information tasks), (2) trust and perceived ease of use (confidence in reliability and accessibility of LLM outputs), (3) behavioral intention (willingness to use LLMs in information-seeking or communication scenarios), and (4) facilitating conditions (eg, privacy concerns, legal accountability, and digital infrastructure).

Figure 1 illustrates the overall workflow of a multicenter, cross-sectional mixed methods observational study conducted across 5 tertiary hospitals in Guangdong, Hunan, Jiangxi, and Guizhou Provinces, China, between August 2024 and March 2025. The study included two participant groups, HCPs and PCs, with parallel questionnaire development and data collection. A total of 260 HCPs and 520 PCs were screened, of whom 240 HCPs and 480 PCs met eligibility criteria and completed the quantitative survey. From these, 15 HCPs and 15 PCs were purposively sampled for qualitative interviews. Quantitative data were analyzed using statistical and regression modeling, while qualitative data underwent thematic coding and interpretation. Findings were integrated through mixed methods triangulation to generate consolidated insights and practical recommendations for trust-based and equitable adoption of LLMs in health care.

All items were rated on 5-point Likert scales (1=strongly disagree and 5=strongly agree). The outcome variable is willingness to adopt LLMs (binary). Predictors include trust, perceived usefulness, digital literacy, previous exposure, and socioeconomic context. Potential confounders include age, gender, education, and institutional type.

Data were collected using secure QR code–based online surveys or paper forms, depending on site logistics. Trained assistants supported participants with limited literacy to ensure data integrity. The instruments were validated via expert review, pilot testing (n=30), and Cronbach α reliability (>0.85 across constructs).

### Qualitative Interviews

To complement quantitative findings, we conducted semistructured interviews with 30 participants (15 HCPs and 15 PCs) purposively selected based on demographic and digital literacy diversity. Interviews lasted 30‐45 minutes, were conducted in private rooms or encrypted online sessions, and followed a guide covering (1) perceived benefits and risks of LLMs; (2) trust, accuracy, and data privacy; (3) expectations for accountability and ethical safeguards; and (4) anticipated impact on patient-provider communication.

Interviews were conducted on-site by trained research assistants at each participating hospital, following a standardized interview guide. A total of two external qualitative experts—a public health researcher and a clinical psychologist, both experienced in health communication research—subsequently reviewed the audio recordings and deidentified transcripts to evaluate thematic coherence and methodological rigor. The interview guide was pilot-tested among 3 participants to ensure clarity and relevance. No repeat interviews were conducted. Data saturation was reached when no new themes emerged after approximately 25 interviews. Transcripts were not returned to participants for comment.

All interviews were audio recorded with consent, transcribed verbatim, deidentified, and analyzed inductively using NVivo (NVivo qualitative data analysis software, QSR International) and the R Qualitative Data Analysis package. In total, 2 coders independently conducted open and axial coding, achieving intercoder reliability (κ=0.82). An inductive thematic analysis approach guided by grounded theory principles was adopted to identify key patterns and relationships. Thematic analysis yielded major categories and subthemes, supported by visualization techniques (node diagrams, frequency plots, and Sankey diagrams).

### Quantitative and Predictive Analyses

Quantitative analyses were performed using Python (version 3.12) and R software (version 4.3, R Foundation for Statistical Computing). Descriptive statistics were reported as means (SDs) for continuous variables and frequencies (%) for categorical variables. Group differences were assessed using Chi-square tests and independent-sample *t* tests (2-sided) as appropriate.

No missing data were identified in the dataset. All questionnaire items were mandatory in the electronic version, and paper-based responses were reviewed immediately by trained research assistants to ensure completeness. As such, the final dataset contained 100% valid observations across all variables, and no imputation or missing completely at random testing was required.

To identify determinants of adoption intent, we estimated multivariable logistic regression (LR) models, controlling for demographic and contextual covariates. To complement regression, 2 machine learning approaches—random forest (RF) and extreme gradient boosting (XGBoost)—were implemented to explore nonlinear patterns and improve the prediction of willingness to use LLMs. Model training used stratified sampling (HCPs: 168/240 and PCs: 336/480, 70% training and HCPs 72/240 and PCs 144/480, 30% validation), with 5-fold cross-validation applied to reduce overfitting.

Model performance was assessed primarily through discrimination (area under the receiver operating characteristic curve [AUC]) and calibration (Brier score and calibration plots). While conventional clinical decision-making metrics (eg, decision curve analysis) were not central to this study, additional evaluation measures such as *F*_1_-score, precision, recall, and accuracy were included to examine classification quality. Variable importance rankings and Shapley additive explanation (SHAP) values were used to enhance interpretability, highlighting the most influential factors shaping adoption intent (eg, digital literacy, trust, and privacy concerns). An overview of the statistical modeling and analytical procedures is provided in Multimedia Appendix 3**.**

### Ethical Considerations

#### Ethics Review and Approval

This study was reviewed and approved by the institutional review boards or ethics committees of all participating hospitals, including Guizhou Provincial People’s Hospital (ethics number: 2024004), the Third Affiliated Hospital of Sun Yat-sen University (ethics number: B2023074), the Third Xiangya Hospital of Central South University (ethics number: 2024011), the University of Hong Kong–Shenzhen Hospital (ethics number: B2024021), and Jiangxi Cancer Hospital (ethics number: JC2024006). The research protocol complied with the Declaration of Helsinki (2013 revision) and local institutional regulations governing research involving human participants.

#### Informed Consent

All participants provided written informed consent before participation. The consent process included clear explanations of study aims, data collection procedures, voluntary participation, and withdrawal rights. For the qualitative component, participants were also informed of audio recording and transcription procedures. No waiver of consent was applied.

#### Privacy and Confidentiality

Participant data were anonymized and deidentified before analysis. No personally identifying information (eg, names, contact details, and hospital ID numbers) was collected. Data were stored on password-protected institutional servers with access restricted to authorized research team members only. All analyses were conducted using deidentified datasets to ensure confidentiality and compliance with institutional data protection standards.

#### Participant Compensation

Participants did not receive financial compensation for participation. However, small tokens of appreciation (eg, stationery or refreshments of RMB ¥30, approximately US $4) were provided in accordance with local ethical norms to acknowledge participants’ time.

#### Image and Identifiable Data Protection

No images or materials containing identifiable personal information are included in the manuscript or supplementary materials. All figures are based on aggregated or simulated data. If any future publication materials include identifiable content, appropriate written consent will be obtained and submitted to the editorial office in accordance with Journal of Medical Internet Research policies.

## Results

### Participant Characteristics

Table 1 summarizes the sociodemographic and professional characteristics of HCPs (eg, role, department, years of experience, and attitudes toward digital tools), while Table 2 presents PCs demographic features (eg, age, gender, education, digital literacy, and perspectives on AI-assisted interpretation).

**Table 1.** Characteristics of health care professionals (HCPs, n=240).

Variables	Values
Sex, n (%)	
Male	141 (58.8)
Female	99 (41.3)
Age (years), mean (SD)	39.9 (6.5)
Profession, n (%)	
Physician	159 (66.2)
Nurse	81 (33.8)
Years of clinical experience, mean (SD)	9.9 (4.8)
Department, n (%)	
Internal medicine	54 (22.5)
Surgery	41 (17.1)
ICU^a^	47 (19.6)
Emergency	60 (24.0)
Other	38 (15.8)
Hospital affiliation, n (%)	
Nonaffiliated	101 (42.1)
Affiliated	139 (57.9)
Hospital type, n (%)	
Oncology	44 (18.3)
General	196 (81.7)
Province economic level, n (%)	
Low	46 (19.2)
Medium	101 (42.1)
High	93 (38.7)
Frequency of digital tool use, n (%)	
Rarely	38 (15.8)
Occasionally	85 (35.4)
Often	117 (48.8)
Prior awareness of LLMs^b^, n (%)	
Yes	166 (69.2)
No	74 (30.8)
Prior use of LLMs, n (%)	
Yes	88 (36.7)
No	152 (63.3)
Received AI^c^ or informatics training, n (%)	
Yes	92 (38.3)
No	148 (61.7)
Trust in LLMs (1-5), mean (SD)	3.09 (1.21)
Perceived usefulness (1-5), mean (SD)	2.98 (1.25)
Privacy concerns (1-5), mean (SD)	3.23 (1.33)
Clarity on legal responsibility (1-5), mean (SD)	3.11 (1.32)

a ICU: intensive care unit.

b LLM: large language model.

c AI: artificial intelligence.

**Table 2.** Characteristics of patients and caregivers (PCs, n=480).

Variables	Values
Sex, n (%)	
Male	242 (50.4)
Female	238 (49.6)
Age (years), mean (SD)	50.1 (12.6)
Role, n (%)	
Patient	360 (75.0)
Caregiver	120 (25.0)
Education level, n (%)	
Primary school or below	121 (25.2)
Junior high school	100 (20.8)
High school	96 (20.0)
College	91 (19.0)
Bachelor’s degree or above	72 (15.0)
Hospital affiliation, n (%)	
Nonaffiliated	197 (41.0)
Affiliated	283 (59.0)
Hospital type, n (%)	
Oncology	94 (19.6)
General	386 (80.4)
Province economic level, n (%)	
Low	88 (18.3)
Medium	202 (42.1)
High	190 (39.6)
Frequency of digital tool use, n (%)	
Rarely	95 (19.8)
Occasionally	162 (33.7)
Often	223 (46.5)
Previous awareness of LLMs^a^, n (%)	
Yes	247 (51.5)
No	233 (48.5)
Previous use of LLMs, n (%)	
Yes	125 (26.0)
No	355 (74.0)
Trust in LLMs (1-5), mean (SD)	2.83 (1.22)
Perceived usefulness (1-5), mean (SD)	2.61 (1.14)
Privacy concerns (1-5), mean (SD)	2.72 (1.29)

a LLM: large language model.

In Table 1, data were collected from 5 tertiary hospitals located in Guangdong, Hunan, Jiangxi, and Guizhou Provinces, China, between August 2024 and March 2025. Variables include age, gender, profession, years of clinical experience, department type, hospital affiliation and type, provincial economic level, frequency of digital tool use, prior awareness and use of LLMs, and receipt of AI or informatics training. In addition, self-reported perceptions—trust in LLMs, perceived usefulness, privacy concern, and clarity on legal responsibility—were measured using a 5-point Likert scale (1=strongly disagree and 5=strongly agree).

Table 2 presents the demographic, educational, and digital behavior characteristics of patients or caregivers who participated in a multicenter, cross-sectional mixed methods observational study on the adoption of LLMs in health care. Data were collected from 5 tertiary hospitals located in Guangdong, Hunan, Jiangxi, and Guizhou Provinces, China, between August 2024 and March 2025. Variables include age, gender, education level, role (patient or caregiver), hospital affiliation and type, provincial economic level, frequency of digital tool use, previous awareness and use of LLMs, and attitudinal measures—including trust in LLMs, perceived usefulness, and privacy concerns—assessed using a 5-point Likert scale (1=strongly disagree and 5=strongly agree).

A total of 720 individuals (240/720 HCPs and 480/720 PCs) participated in the study (Tables1  2). Among HCPs, two-thirds were physicians (159/240, 66.2%) and one-third nurses (81/240, 33.8%), with an average age of 39.9 (SD 6.5) years and 9.9 (SD 4.8) years of clinical experience. Participants were drawn from diverse departments, with representation from internal medicine, surgery, intensive care, and emergency care. Almost half (117/240, 48.8%) reported frequent use of digital tools, and 166/240 (69.2%) were already aware of LLMs, though only 36.7% (88/240) had previous experience using them.

Patients or caregivers were evenly distributed by sex (242/480, 50.4% male), with a mean age of 50.1 (SD 12.6) years. Educational attainment varied considerably: 121/480 (25.2%) PCs had only primary schooling or below, while 15.0% (72/480) held a Bachelor’s degree or higher. Frequent digital tool use was reported by 15.0% (72/480), and about half (247/480, 51.5%) had heard of LLMs, though only 26.0% (125/480) had ever used them. These characteristics reflected broad heterogeneity in digital readiness and information familiarity across both user groups.

### Group Differences in Perceptions of LLMs

Perceptions of LLMs varied significantly between HCPs and PCs. On a 5-point Likert scale, HCPs rated perceived usefulness higher (mean 2.98, 95% CI 2.86‐3.10 vs mean 2.61, 95% CI 2.52‐2.70; *P*<.001) and expressed stronger privacy concerns (mean 3.23, 95% CI 3.12‐3.34 vs mean 2.72, 95% CI 2.63‐2.81; *P*<.001). Trust in LLMs, however, was comparable between groups (mean 3.09, 95% CI 2.97‐3.21 vs mean 2.83, 95% CI 2.72‐2.94; *P*=.20). These results suggest that HCPs view LLMs as potentially valuable but remain cautious about risks, while PCs perceive fewer benefits but also fewer risks (Multimedia Appendix 4).

### Correlates of Adoption Willingness

Among HCPs, willingness to adopt LLMs was positively associated with previous awareness and use (*P*<.01), frequent digital tool use (*P*=.02), AI or informatics training (*P*=.03), higher trust (*P*<.001), and perceived usefulness (*P*<.001). In contrast, privacy concerns (*P*=.04) and uncertainty about legal accountability (*P*=.02) reduced willingness (Table 3).

**Table 3.** Bivariate comparisons of characteristics by willingness to adopt large language models among health care professionals. Associations were tested using *t* tests for continuous variables and Chi-square tests for categorical variables.

Variables	Willing	Unwilling or uncertain	*P* value^a^
Sex, n (%)			.927
Male	69/116 (59.5)	72/124 (58.1)	
Female	47/116 (40.5)	52/124 (41.9)	
Profession, n (%)			.712
Physician	75/116 (64.7)	84/124 (67.7)	
Nurse	41/116 (35.3)	40/124 (32.3)	
Department, n (%)			.751
Internal medicine	27/116 (23.3)	27/124 (21.8)	
Surgery	22/116 (19)	19/124 (15.3)	
ICU^b^	22/116 (19)	25/124 (20.2)	
Emergency	25/116 (21.6)	35/124 (28.2)	
Other	20/116 (17.2)	18/124 (14.5)	
Hospital affiliation, n (%)			.137
Nonaffiliated	55/116 (47.4)	46/124 (37.1)	
Affiliated	61/116 (52.6)	78/124 (62.9)	
Hospital type, n (%)			.938
Oncology	22/116 (19)	22/124 (17.7)	
General	94/116 (81)	102/124 (82.3)	
Province’s economic level, n (%)			<.001
Low	11/116 (9.5)	35/124 (28.2)	
Medium	34/116 (29.3)	59/124 (47.6)	
High	71/116 (61.2)	30/124 (24.2)	
Frequency of digital tool use, n (%)			<.001
Rarely	5/116 (4.3)	33/124 (26.6)	
Occasionally	36/116 (31)	49/124 (39.5)	
Often	75/116 (64.7)	42/124 (33.9)	
Previous awareness of LLMs^c^, n (%)			<.001
Yes	93/116 (80.2)	73/124 (58.9)	
No	23/116 (19.8)	51/124 (41.1)	
Previous use of LLMs, n (%)			<.001
Yes	56/116 (48.3)	32/124 (25.8)	
No	60/116 (51.7)	92/124 (74.2)	
Received AI^d^ or informatics training, n (%)			.030
Yes	53/116 (45.7)	39/124 (31.5)	
No	63/116 (54.3)	85/124 (68.5)	
Age (years), mean (SD)	39.5 (6.4)	40.3 (6.6)	.382
Years of clinical experience, mean (SD)	10.7 (5.1)	9.2 (4.4)	.017
Trust in LLMs (1-5), mean (SD)	3.20 (1.22)	2.78 (1.26)	.011
Perceived usefulness (1-5), mean (SD)	3.70 (1.11)	2.52 (1.02)	<.001
Privacy concerns (1-5), mean (SD)	2.82 (1.20)	3.62 (1.33)	<.001
Clarity on legal responsibility (1-5), mean (SD)	3.41 (1.29)	2.84 (1.29)	<.001

a *P*<.05 was considered statistically significant.

b ICU: intensive care unit.

c LLM: large language model.

d AI: artificial intelligence.

Table 3 presents bivariate comparisons (Chi-square tests for categorical variables and independent-sample *t* tests for continuous variables) examining factors associated with willingness to adopt LLMs among health care professionals. Data were derived from a multicenter, cross-sectional mixed methods observational study conducted in 5 tertiary hospitals located in Guangdong, Hunan, Jiangxi, and Guizhou Provinces, China, between August 2024 and March 2025. Variables analyzed include age, gender, profession, department, hospital characteristics, provincial economic level, digital tool use frequency, previous awareness and use of LLMs, receipt of AI or informatics training, and attitudinal measures—trust in LLMs, perceived usefulness, privacy concern, and clarity on legal responsibility—assessed on a 5-point Likert scale (1=strongly disagree and 5=strongly agree).

Among PCs, willingness was more strongly shaped by sociodemographic and contextual factors: higher education level (*P*<.001), better regional economic conditions (*P*<.001), and frequent digital tool use (*P*<.001) were all associated with stronger adoption intent. Trust (*P*<.001) and perceived usefulness (*P*<.001) were again positive predictors, while privacy concerns acted as deterrents (*P*=.01). Unlike HCPs, previous LLM exposure was not a significant factor for PCs (Table 4).

**Table 4.** Bivariate comparisons of characteristics by willingness to adopt large language models among patients and caregivers. Statistical comparisons were performed using *t* tests for continuous variables and Chi-square tests for categorical variables.

Variables	Participants		*P* value^a^
	Willing (n=187)	Unwilling/uncertain (n=293)	
Sex, n (%)			.547
Male	98 (52.4)	144 (49.1)	
Female	89 (47.6)	149 (50.9)	
Role, n (%)			.787
Patient	142 (75.9)	218 (74.4)	
Caregiver	45 (24.1)	75 (25.6)	
Education level, n (%)			<.001
Primary school or below	19 (10.2)	86 (29.4)	
Junior high school	28 (15)	66 (22.5)	
High school	40 (21.4)	65 (22.2)	
College	58 (31)	45 (15.4)	
Bachelor’s degree or above	42 (22.5)	31 (10.6)	
Hospital affiliation, n (%)			.886
Nonaffiliated	78 (41.7)	119 (40.6)	
Affiliated	109 (58.3)	174 (59.4)	
Hospital type, n (%)			.462
Oncology	33 (17.6)	61 (20.8)	
General	154 (82.4)	232 (79.2)	
Province economic level, n (%)			<.001
Low	23 (12.3)	67 (22.9)	
Medium	97 (51.9)	61 (20.8)	
High	67 (35.8)	165 (56.3)	
Frequency of digital tool use, n (%)			<.001
Rarely	4 (2.1)	90 (30.7)	
Occasionally	24 (12.8)	138 (47.1)	
Often	159 (85)	65 (22.2)	
Previous awareness of LLMs^b^, n (%)			.177
Yes	102 (54.5)	140 (47.8)	
No	85 (45.5)	153 (52.2)	
Previous use of LLMs, n (%)			.118
Yes	91 (48.7)	120 (41)	
No	96 (51.3)	173 (59)	
Age (years), mean (SD)	49.5 (12.0)	50.5 (13.0)	.410
Trust in LLMs (1-5), mean (SD)	3.02 (1.18)	2.36 (1.04)	<.001
Perceived usefulness (1-5), mean (SD)	3.97 (0.70)	2.10 (0.88)	<.001
Privacy concerns (1-5), mean (SD)	2.21 (1.77)	3.04 (1.25)	<.001

a *P*<.05 considered statistically significant.

b LLM: large language model.

Table 4 presents bivariate comparisons (Chi-square tests for categorical variables and independent-sample *t* tests, 2-tailed for continuous variables) examining factors associated with willingness to adopt LLMs among PCs. Data were obtained from a multicenter, cross-sectional mixed methods observational study conducted in 5 tertiary hospitals located in Guangdong, Hunan, Jiangxi, and Guizhou Provinces, China, between August 2024 and March 2025. Variables analyzed include age, gender, role (PC), education level, hospital affiliation and type, provincial economic level, frequency of digital tool use, previous awareness and use of LLMs, and attitudinal measures—trust in LLMs, perceived usefulness, and privacy concern—assessed on a 5-point Likert scale (1=strongly disagree and 5=strongly agree).

### Independent Predictors From Multivariate Modeling

Multivariable LR highlighted both common and distinct predictors across groups.

**Figure 2.**
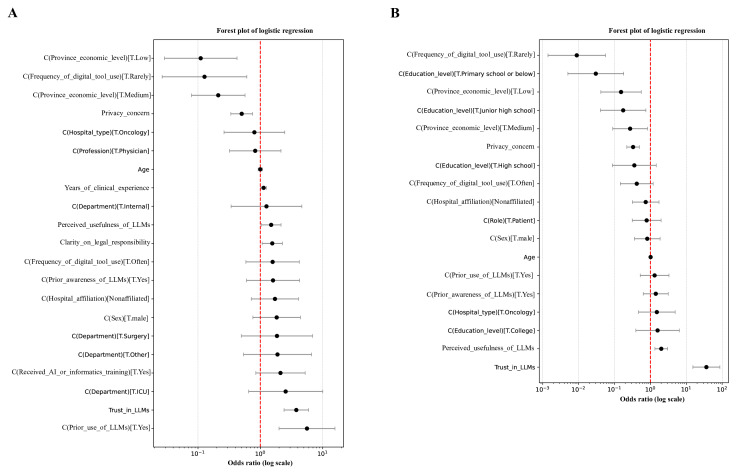
Multivariate logistic regression models identifying predictors of willingness to adopt large language models (**A**) healthcare professionals, (B) patients or caregivers. Values are shown on a logarithmic scale, with the red dashed line indicating an OR of 1 (no effect). Variables positioned to the right of the line indicate positive associations with large language model adoption willingness, while those to the left indicate negative associations. HCP: health care professional; LLM: large language model; OR: odds ratio; PC: patient or caregiver.

For HCPs, adoption was most strongly predicted by trust (odds ratio [OR] 3.78, 95% CI 2.15‐6.63; *P*<.001), previous use (OR 5.61, 95% CI 3.02‐10.44; *P*<.001), and clarity regarding legal responsibility (OR 1.56, 95% CI 1.07‐2.27; *P*=.02). Privacy concerns were a negative predictor (OR 0.72, 95% CI 0.53‐0.97; *P*=.03) (Table S3 in Multimedia Appendix 2 and Multimedia Appendix 5; Figure 2A).

Figure 2 presents forest plots from multivariate logistic regression analyses examining predictors of willingness to adopt LLMs among 2 user groups: HCPs and PCs. Data were derived from a multicenter, cross-sectional mixed methods observational study conducted in 5 tertiary hospitals across Guangdong, Hunan, Jiangxi, and Guizhou Provinces, China, between August 2024 and March 2025. Each plot displays the ORs with 95% CIs for independent variables including demographic characteristics (eg, age, gender, and education), digital behavior (eg, frequency of digital tool use, previous use or awareness of LLMs), and attitudinal factors (eg, trust, perceived usefulness, privacy concern, and legal clarity).

For patients or caregivers, trust (OR 36.34, 95% CI 18.41‐71.74; *P*<.001) dominated as the single strongest determinant, alongside perceived usefulness (OR 2.01, 95% CI 1.52‐2.67; *P*<.001). Lower education, infrequent digital tool use, and disadvantaged economic background significantly reduced adoption likelihood (Table S4 in Multimedia Appendix 2; Figure 2B).

These results underscore that attitudinal and informational readiness factors (trust, usefulness, and digital literacy) are more predictive of willingness than static demographic variables.

### Model Performance and Interpretability

**Figure 3.**
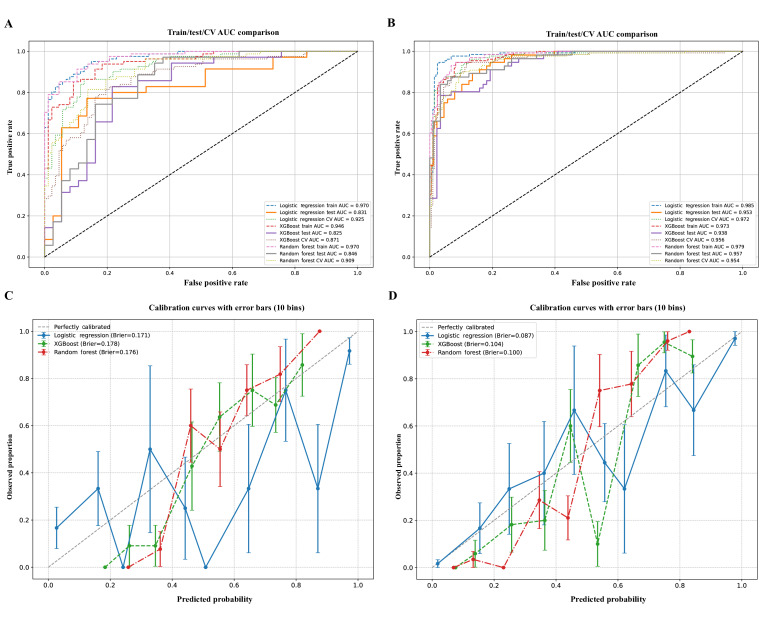
Discriminative and calibration performance of logistic regression, random forest, and extreme gradient boosting models in predicting willingness to adopt large language models. (**A**) Receiver operating characteristic curves for health care professionals, (**B**) Receiver operating characteristic curves for patients or caregivers, (**C**) Calibration curves with 10-bin error bars for health care professionals, (**D**) Calibration curves with 10-bin error bars for patients or caregivers. (A) and (B) depict ROC curves comparing training, testing, and CV performance across models, with AUC values presented for each subset. Panels (C) and (D) display calibration curves with 10-bin error bars, comparing predicted probabilities to observed outcomes; the dashed diagonal line represents perfect calibration. AUC: area under the receiver operating characteristic curve ; CV: cross-validation.

Across LR, RF, and XGBoost, predictive performance was strong, with AUCs ranging from 0.83‐0.85 among HCPs and 0.93‐0.96 among PCs. Classification metrics (precision, recall, and *F*_1_-score) confirmed robustness across models (Figures3^5^; MultimediaAppendices 6  7; Table 5; and Tables S5-S8 in Multimedia Appendix 2).

**Figure 4.**
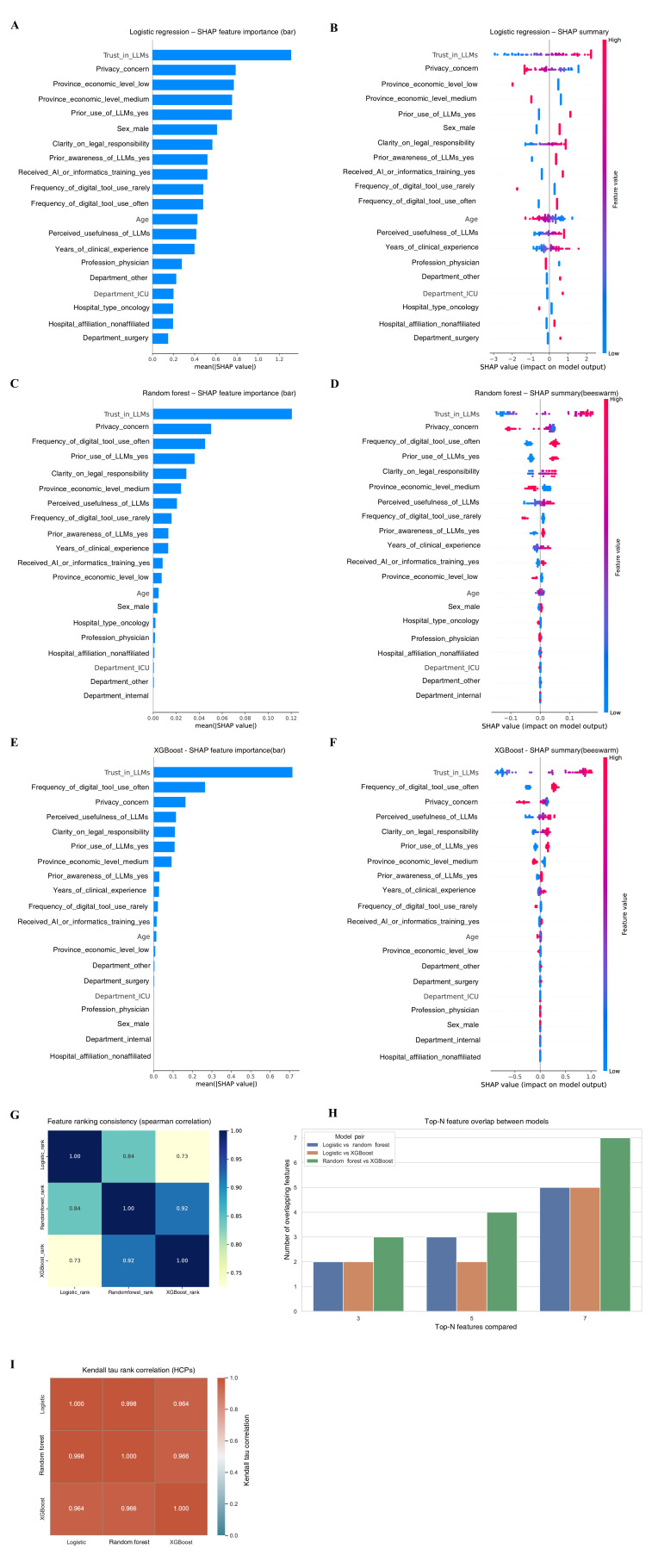
Model interpretability and cross-model agreement in predicting large language model adoption willingness among health care professionals. Shapley additive explanations bar plot (**A**) and beeswarm plot (**B**) for logistic regression. Shapley additive explanations bar plot (**C**) and beeswarm plot (**D**) for random forest. SHAP bar plot (**E**) and beeswarm plot (**F**) for extreme gradient boosting. (**G**) Heatmap of feature importance rank consistency across the 3 models. (**H**) Overlap in the top 3, 5, and 7 ranked features across models. (**I**) Kendall tau rank correlation matrix assessing agreement in feature importance rankings across models. High concordance of feature rankings (G-I) confirmed model robustness and cross-model agreement, with correlation coefficients exceeding 0.9 across methods. AI: artificial intelligence; HCP: health care professional; ICU: intensive care unit; LLM: large language models; SHAP: Shapley additive explanations; XGBoost: extreme gradient boosting.

**Figure 5.**
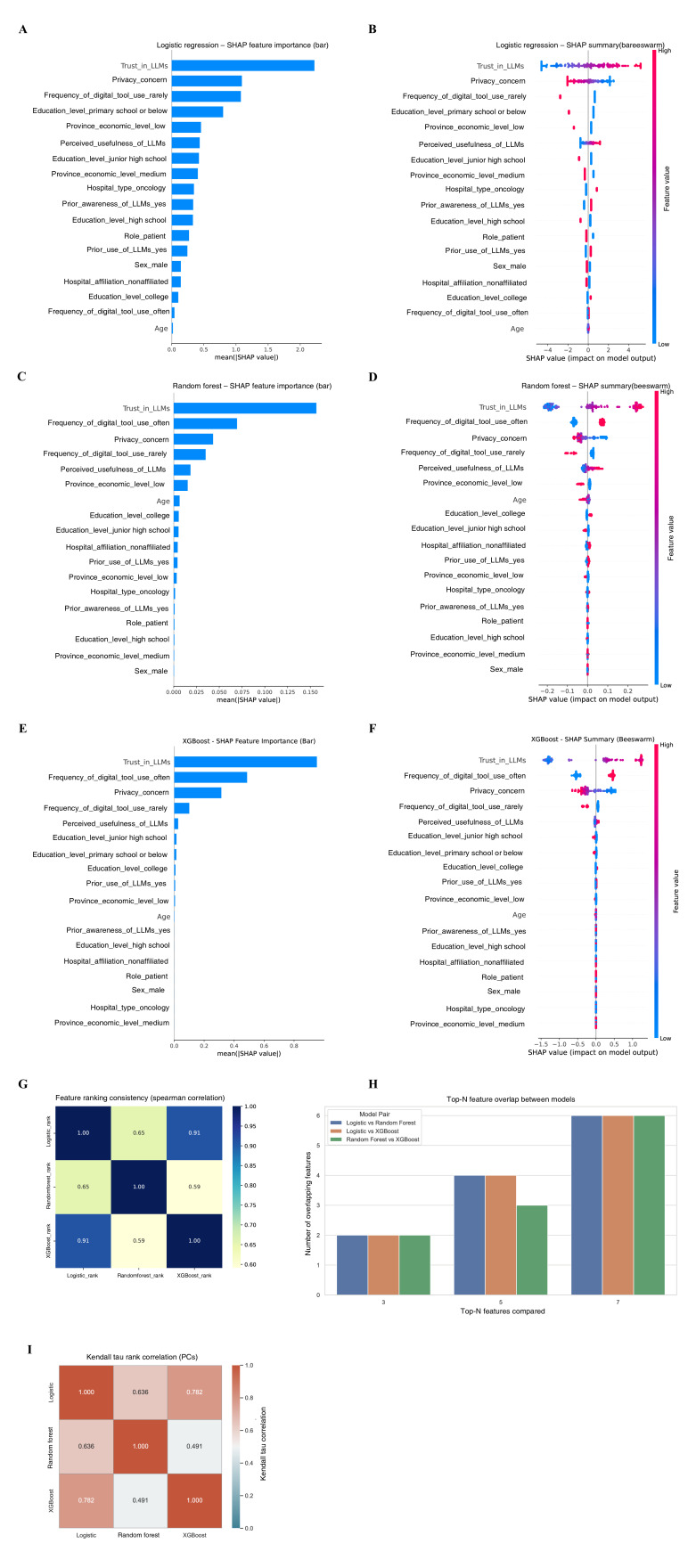
Model interpretability and cross-model agreement in predicting large language models’ adoption willingness among patients or caregivers. (**A**) Shapley additive explanations bar plot and (**B**) beeswarm plot for logistic regression. (**C**) Shapley additive explanations bar plot and (**D**) beeswarm plot for random forest. (**E**) Shapley additive explanations bar plot and (**F**) beeswarm plot for extreme gradient boosting. (**G**) Heatmap of feature importance rank consistency across the three models. (**H**) Overlap in the top 3, 5, and 7 ranked features across models. (**I**) Kendall tau rank correlation matrix assessing agreement in feature importance rankings across models. Cross-model comparisons (G-I) demonstrated strong consistency in feature rankings, with high Spearman and Kendall correlation coefficients indicating robust agreement in variable importance across the 3 algorithms. LLM: large language model; PC: patients or caregivers; SHAP: Shapley additive explanations; XGBoost: extreme gradient boosting.

Figure 3 summarizes the discrimination and calibration performance of three predictive models—LR, RF, and XGBoost—used to estimate willingness to adopt LLMs among HCPs and PCs. Data were obtained from a multicenter, cross-sectional mixed methods observational study conducted in five tertiary hospitals across Guangdong, Hunan, Jiangxi, and Guizhou Provinces, China, between August 2024 and March 2025. Brier scores are reported to quantify prediction error. All models were trained using stratified 5-fold CV with a held-out validation set, ensuring balanced sampling and robust generalizability across user groups.

Figure 4 illustrates model interpretability and feature importance comparisons for predicting HCPs' willingness to adopt LLMs in clinical and research contexts. Data were derived from a multicenter cross-sectional survey of 240 physicians and nurses conducted between August 2024 and March 2025 across 5 tertiary hospitals in Guangdong, Hunan, Jiangxi, and Guizhou Provinces, China. Three supervised machine learning algorithms—logistic regression, random forest, and XGBoost—were trained to identify key determinants of LLM adoption willingness. Predictor variables encompassed demographic, professional, and attitudinal factors, including trust in LLMs, privacy concern, perceived usefulness, frequency of digital-tool use, prior exposure to AI or informatics training, and clarity of legal responsibility. Across all models, trust in LLMs and privacy concern emerged as the most influential predictors, indicating that sociotechnical and psychological readiness outweighed demographic or institutional attributes in shaping adoption intent.

Figure 5 presents model interpretability and feature-importance comparisons for predicting PCs' willingness to adopt LLMs for medical information seeking and decision support. Data were obtained from a multicenter cross-sectional survey of 480 adult patients and caregivers conducted between August 2024 and March 2025 across 5 tertiary hospitals in Guangdong, Hunan, Jiangxi, and Guizhou Provinces, China. Three supervised machine learning algorithms—logistic regression, random forest, and XGBoost—were trained to identify key determinants of LLM adoption willingness. Predictor variables included trust in LLMs, privacy concern, perceived usefulness, digital tool use frequency, education level, prior exposure to AI or LLMs, and regional economic context. Across all models, trust in LLMs consistently emerged as the dominant positive predictor of adoption willingness, whereas privacy concern and low education level were the primary barriers. Frequent digital tool use and higher perceived usefulness further increased the likelihood of adoption. These findings highlight that, among patients and caregivers, trust and digital readiness outweighed demographic or institutional factors in determining openness to adopting LLM-based health information tools.

**Table 5.** Pairwise DeLong test results for the areas under the receiver operating characteristic curves comparison across models (health care professionals and patients or caregivers).

Model 1	Model 2	AUC^a^ 1	AUC 2	ΔAUC	*P* value
Health care professionals					
Logistic regression	XGBoost^b^	0.831	0.825	0.006	.90
Logistic regression	Random forest	0.831	0.846	–0.015	.70
XGBoost	Random forest	0.825	0.846	–0.022	.30
Patients or caregivers					
Logistic regression	XGBoost	0.953	0.938	0.014	.33
Logistic regression	Random forest	0.953	0.957	–0.004	.78
XGBoost	Random forest	0.938	0.957	–0.018	.02

a AUC: area under the receiver operating characteristic curve.

b XGBoost: extreme gradient boosting.

Table 5 summarizes the results of pairwise DeLong tests comparing the AUCs for 3 predictive models—logistic regression, random forest, and XGBoost—used to estimate the willingness to adopt large language models (LLMs) in health care. Analyses were performed separately for HCPs and PCs based on data from a multicenter, cross-sectional mixed methods observational study conducted in 5 tertiary hospitals across Guangdong, Hunan, Jiangxi, and Guizhou Provinces, China, between August 2024 and March 2025. The DeLong test was applied to assess whether differences in model discrimination (AUC values) were statistically significant, providing a measure of the comparative robustness and generalizability of predictive models across user groups.

Interpretability analyses (SHAP values) consistently identified trust in LLMs as the most influential predictor of adoption across both groups. Among HCPs, previous use, legal clarity, and digital tool use also ranked highly. For PCs, education and regional economic status were more prominent, reflecting underlying disparities in digital readiness. Demographic and institutional factors (eg, age, department, and hospital type) had minimal predictive power.

### Marginal Effects and Interaction Patterns

**Figure 6.**
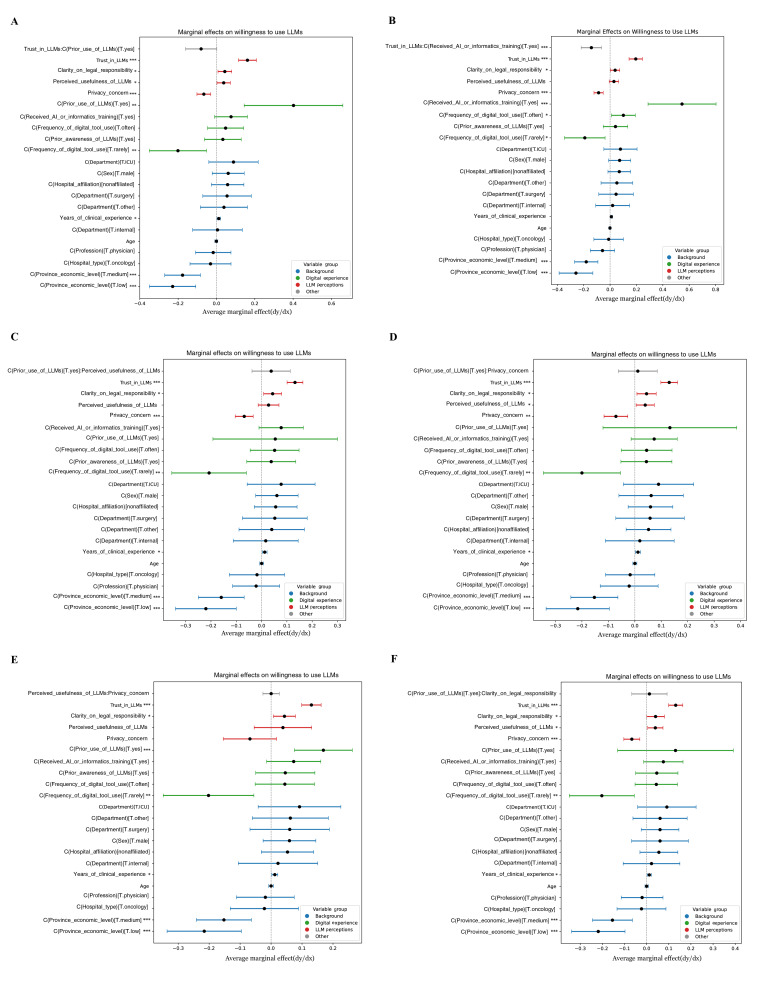
Marginal effects of key predictors on willingness to adopt large language models among health care professionals. (**A**) Interaction between trust in large language models and prior use of large language models. (**B**) Interaction between trust in large language models and receipt of AI or informatics training. (**C**) Interaction between prior use of large language models and perceived usefulness. (**D**) Interaction between prior use of large language models and privacy concerns. (**E**) Interaction between perceived usefulness and privacy concern. (**F**) Interaction between prior use of large language models and clarity on legal responsibility. LLM: large language models.

Marginal effects analysis confirmed that trust in LLMs exerted the strongest positive effect on adoption across both groups, while privacy concern consistently deterred adoption. Among HCPs, previous use amplified the effect of trust, whereas among PCs, education and digital literacy had stronger moderating roles. Interaction effects were limited but indicated that combinations such as trust×legal clarity (for HCPs) and usefulness×previous exposure (for PCs) enhanced adoption intent (Figures6^7^, MultimediaAppendices 5, 8^11^).

**Figure 7.**
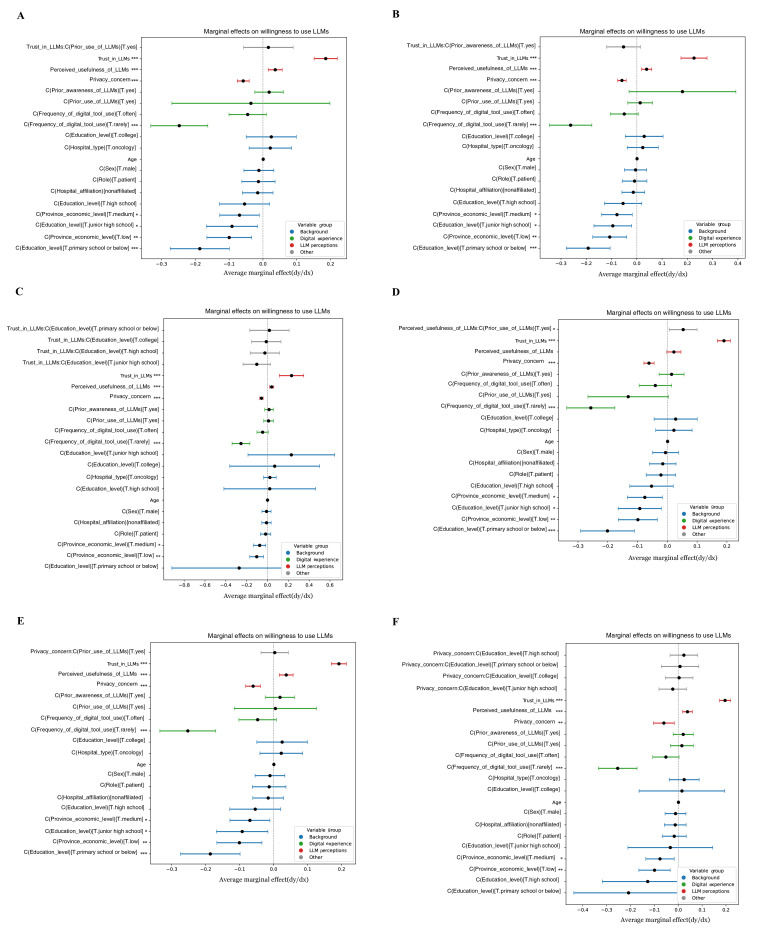
Marginal effects of key predictors on willingness to adopt large language models among patients or caregivers. (**A**) Interaction between trust in large language models and prior use of LLMs. (**B**) Interaction between trust in large language models and prior awareness of large language models. (**C**) Interaction between trust in large language models and low education level (primary school or below). (**D**) Interaction between prior use of large language models and perceived usefulness. (**E**) Interaction between prior use of large language models and privacy concern. (**F**) Interaction between privacy concern and education level (high school). LLM: large language models.

Figure 6 depicts the marginal effects and interaction patterns of key predictors on HCPs’ willingness to adopt LLMs for clinical and research purposes. Data were derived from a multicenter cross-sectional survey of 240 physicians and nurses conducted between August 2024 and March 2025 across 5 tertiary hospitals in Guangdong, Hunan, Jiangxi, and Guizhou Provinces, China. Multivariable logistic regression models were estimated with average marginal effects to visualize how trust, prior exposure, perceived usefulness, privacy concern, and legal clarity interact to influence adoption intent. Positive marginal effects (points to the right of zero) indicate increased probability of adoption. Across all interactions, trust in LLMs consistently amplified the effect of favorable experience or clear governance conditions, whereas privacy concern attenuated these relationships. These findings suggest that institutional training and regulatory clarity strengthen the trust–usefulness pathway underlying professionals’ readiness to integrate LLMs into practice.

Figure 7 illustrates the marginal effects and interaction patterns of major predictors influencing PCs’ willingness to adopt LLMs for health information seeking and decision support. Data were obtained from a multicenter cross-sectional survey of 480 adults conducted between August 2024 and March 2025 across 5 tertiary hospitals in Guangdong, Hunan, Jiangxi, and Guizhou Provinces, China. Average marginal effects were derived from multivariable logistic regression models to visualize how trust, previous exposure, perceived usefulness, privacy concern, and education level jointly shape adoption intent. Positive marginal effects represent increased probability of LLM adoption, whereas negative values indicate deterrent effects. Across models, trust in LLMs remained the strongest positive driver, amplifying the influence of prior awareness and perceived usefulness, while privacy concern and low educational attainment significantly weakened adoption intent. These results underscore that, among PCs, cognitive accessibility and trust interact closely with privacy literacy and digital readiness to determine willingness to engage with AI-enabled health information systems.

### Qualitative Insights

**Figure 8.**
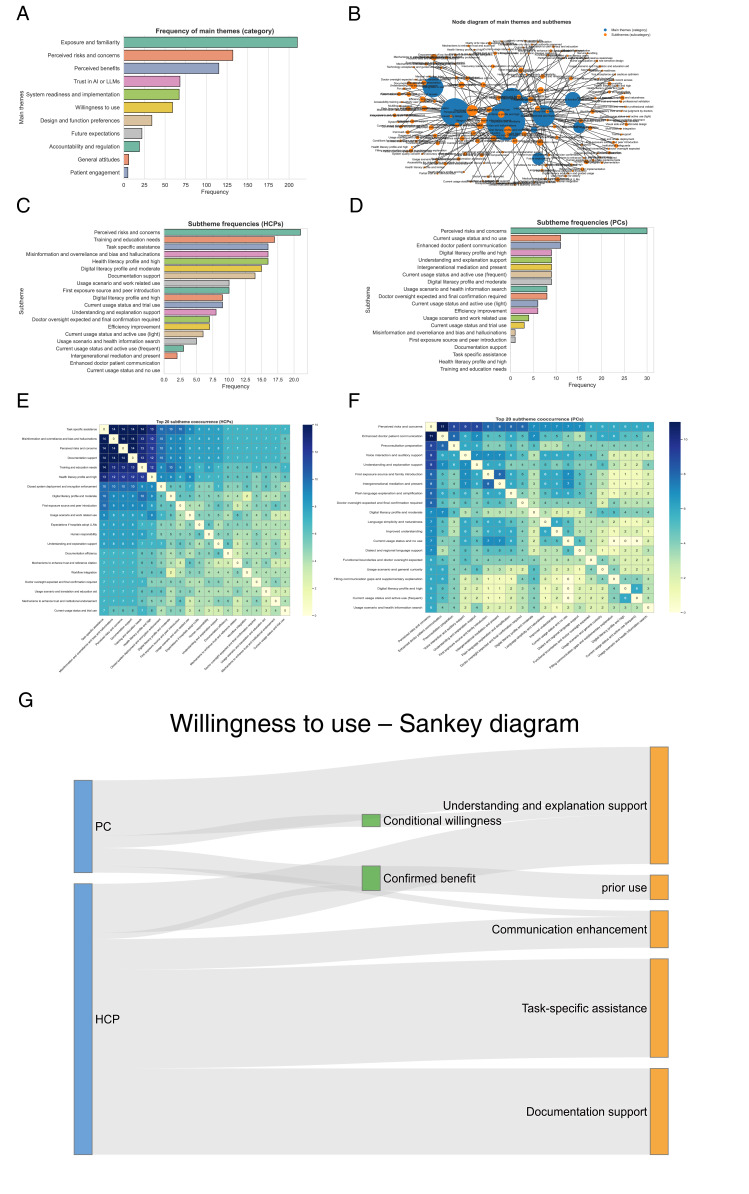
Thematic structure, key subthemes, and motivational patterns shaping willingness to adopt large language models across health care professionals and patients or caregivers. (**A**) Theme frequency distribution. (**B**) Subtheme node diagram. (**C**) Top 20 subthemes in health care professionals. (**D**) Top 20 subthemes in patients or caregivers. (**E**) Subtheme co-occurrence in health care professionals. (**F**) Subtheme co-occurrence in patients or caregivers. (**G**) Motivational pathways for large language model adoption (Sankey). HCP: health care professional; LLM: large language model; PC: patients or caregivers.

Semistructured interviews (n=30) revealed 11 thematic categories providing depth to the survey findings (Figure 8). Representative quotations from participants (eg, “HCP#3, female, oncology day care unit nurse;” “PC#8, male, caregiver”) were included to illustrate key themes and ensure analytic transparency.

Figure 8 summarizes the qualitative thematic analysis describing how HCPs and PCs conceptualize the benefits, risks, and conditions influencing their willingness to adopt LLMs in health care. Data were drawn from semistructured interviews with 30 participants (15 HCPs and 15 PCs) conducted between August 2024 and March 2025 across 5 tertiary hospitals in Guangdong, Hunan, Jiangxi, and Guizhou Provinces, China. Transcripts were coded inductively following thematic and co-occurrence analysis to identify dominant categories, subthemes, and motivational linkages. Overall, qualitative findings indicate that both HCPs and PCs perceive LLM adoption as a trust-dependent, literacy-mediated process shaped by previous exposure, perceived utility, and contextual reassurance through institutional safeguards.

Health care professionals emphasized risks and responsibilities, focusing on concerns about misinformation, privacy, and institutional accountability. They also stressed the need for structured onboarding, training, and workflow integration.

Patients or caregivers prioritized accessibility and comprehensibility, particularly among participants with lower digital literacy. Themes such as plain-language explanations, conversational interaction, and support for understanding medical information emerged strongly.

Across both groups, trust surfaced repeatedly as the foundation for adoption, while digital readiness shaped whether users perceived LLMs as beneficial or burdensome. Thematic co-occurrence analyses highlighted that risk-related concerns were often tied to calls for oversight and institutional safeguards. Patients or caregivers’ adoption intent was more closely tied to communication benefits, while HCPs’ intent was more closely tied to efficiency and professional utility.

## Discussion

### Summary of Main Findings

This multicenter mixed methods study investigated how trust, perceived usefulness, and sociotechnical readiness influence willingness to adopt LLMs among HCPs and PCs in China. Consistent with our study objectives, quantitative analyses and multivariate models demonstrated that trust in LLMs was the most significant predictor for both groups. Model performance remained robust across approaches, and interpretability analyses consistently identified trust as the most influential feature. Interaction analyses further revealed that trust, privacy concern, and digital literacy jointly shaped adoption trajectories.

Complementary qualitative findings echoed these patterns: HCPs emphasized professional accountability and workflow integration, whereas PCs valued comprehension, reassurance, and accessible communication. Together, the mixed methods evidence indicates that the adoption of generative AI in health care depends less on algorithmic performance and more on human-centered and ethical readiness.

### Trust as a Central Determinant

Trust emerged as the cornerstone of adoption intentions for both clinicians and patients [42,43]. From a sociotechnical perspective, trust encompasses confidence in technical reliability, institutional endorsement, and perceived integrity of AI outputs. Among HCPs, trust was reinforced by prior experience, legal clarity, and professional training, while among PCs, it was closely linked to transparency, interpretability, and educational background [44,45].

These findings suggest that trust functions as a bridge between perceived usefulness and behavioral intention, shaped by previous exposure and regulatory assurance. Therefore, cultivating trust requires not only transparent algorithms but also institutional validation and explainability-by-design—principles essential for ensuring safe AI integration in clinical environments [46,47].

### Perceived Usefulness and Role-Specific Value

Perceived usefulness was another important enabler of adoption, reflecting both functional and cognitive dimensions. For HCPs, usefulness aligned with workflow efficiency gains and decision support, while PCs associated usefulness with plain-language explanations and empathy in communication.

This role-specific distinction illustrates that functional usefulness (improved professional performance) and cognitive usefulness (enhanced understanding and reassurance) are complementary but context-dependent. Designers should therefore tailor AI interfaces to user goals—embedding documentation tools and audit trails for clinicians, while simplifying outputs and promoting conversational transparency for PCs [48-53].

### Barriers: Privacy Concerns, Literacy, and Socioeconomic Inequality

Privacy concerns consistently acted as a barrier to adoption, reflecting apprehensions about data misuse and accountability. Clinicians expressed worries about data liability and auditability, while PCs feared data misuse or unintended disclosure. These findings align with global evidence that perceived privacy risk undermines trust formation and information sharing [54,55].

Furthermore, education and digital tools use moderated adoption, highlighting how the digital divide extends beyond access to include literacy and contextual capability. Participants with lower education or from less developed provinces demonstrated markedly reduced adoption intent. This supports a sociotechnical interpretation of digital inequality—as a function of literacy, institutional support, and participatory capacity rather than infrastructure alone [34,54,55].

To mitigate these barriers, our findings suggest several actionable design and policy interventions. First, privacy-by-design features—such as decentralized data processing, built-in anonymization, and user-controllable consent dashboards—should be integrated into LLM-based systems to minimize exposure risks while preserving functionality. Second, transparent data-use and auditability frameworks are critical for both HCPs and PCs; clear communication of how data are stored, used, and protected can strengthen perceived institutional credibility and accountability.

Third, targeted privacy literacy and digital readiness education programs can empower users to understand their data rights and engage more confidently with AI systems. Finally, context-aware system design, including adaptive privacy settings and local-language interfaces, can bridge literacy and regional disparities.

Together, these strategies operationalize privacy protection as a foundation for trust, transforming user apprehension into informed confidence and facilitating equitable LLM adoption across diverse health care settings.

### Model Performance and Interpretability

Comparative analyses demonstrated that all predictive models—LR, RF, and XGBoost—performed consistently well, confirming the robustness of observed associations. RF achieved the best calibration and interpretability balance, and ensemble approaches offered marginal accuracy gains. Overall, these results reinforce that the identified determinants, especially trust, usefulness, and privacy, are statistically and behaviorally meaningful.

### Qualitative Integration and Thematic Insights

The qualitative component contextualized quantitative predictors by illustrating how users experience them in practice. Health care professionals’ motivation was efficiency- and accountability-driven; PCs’ motivation centered on clarity, empathy, and reduced uncertainty. These qualitative data contextualize the quantitative predictors by showing why these factors matter—trust is experienced as emotional reassurance and institutional safety rather than abstract confidence. This integration underscores the mixed methods value: statistical predictors explain “what matters,” while qualitative insights explain “why it matters.”

### Interpretation of Variability Across Measures

Variability in clinical experience and digital familiarity among participants was expected, given the multicenter design, and enhances generalizability. Differences in perceived usefulness reflected heterogeneity in exposure to digital tools and institutional environments. Such real-world diversity strengthens, rather than weakens, interpretive validity by highlighting subgroup differences that warrant attention in future implementation studies.

### Theoretical Contributions

This study contributes to information management theory in several ways.

First, it extends adoption models such as Technology Acceptance Model and Unified Theory of Acceptance and Use of Technology by empirically demonstrating that trust and ethical risk outweigh demographic factors in shaping adoption intent for AI-driven information tools. By quantifying these relationships across both HCPs and PCs, this research offers a novel, data-driven framework for understanding sociotechnical readiness in digital health [56,57].

Second, it introduces sociotechnical readiness as a critical construct, capturing how digital literacy, institutional support, and privacy concerns jointly influence information behaviors. This theoretical construct bridges individual psychology and institutional systems, offering a holistic model for studying complex technology ecosystems in health care [58-60].

Third, the findings highlight the multidimensional nature of information use, where the same technology offers distinct value propositions depending on user roles and contexts—pointing to the need for adaptive system design and role-based evaluation. This supports a paradigm shift in information systems research: from performance-centric models toward equity- and trust-centered frameworks that emphasize human values and ethical alignment.

Collectively, these contributions support a broader agenda for sociotechnical information systems research, emphasizing equity, governance, and the coconstruction of trust—reinforcing that effective information tools require integration of social factors, institutional policies, and technological affordances rather than standalone technical performance.

### Practical and Policy Implications

From a practical standpoint, the findings of this study point to several priorities for the responsible deployment of LLMs as information systems.

First, trust must be intentionally engineered through transparent interfaces, verifiable information sources, and institutional endorsement mechanisms. Explainable and auditable AI systems can strengthen confidence among HCPs and PCs alike [46].

Second, system design should be role-sensitive—integrating workflow automation and accountability tools for HCPs while offering plain-language, empathetic interfaces for PCs. This aligns with information systems research emphasizing that system value is contingent on matching technologies to user roles and contexts [61].

Third, bridging the digital divide is imperative. Accessibility-by-design, multilingual support, and targeted digital literacy programs are essential for equitable participation. Evidence shows that the digital divide in AI use is not only about infrastructure, but also about literacy and sociotechnical support systems, requiring intentional interventions to foster equitable participation [35].

Finally, governance frameworks must evolve to clarify liability, privacy protection, and data sovereignty, ensuring that trust is institutionally maintained, not individually assumed. Collectively, these strategies translate theoretical findings into actionable design and policy recommendations, advancing the responsible and inclusive adoption of AI in health care [62,63].

### Model Robustness and Potential Overfitting

Although the sample size for both HCPs and PCs was modest, multiple strategies were employed to minimize overfitting and enhance the generalizability of our models. Nested five-fold cross-validation and independent test evaluation demonstrated that model performance was stable across folds, with minimal variance between test and cross-validation AUCs. Regularization and feature selection procedures further reduced dimensionality and noise. The consistent performance of LR, RF, and XGBoost models—alongside similar AUC and Brier scores—supports the robustness of findings.

### Model Selection and Interpretability Considerations

Although all 3 models—LR, RF, and XGBoost—demonstrated comparable discriminative performance and stable calibration, LR was identified as the optimal model for interpretation and practical deployment.

Its parsimony, transparency, and statistical stability make it particularly suitable for health care apps, where explainability and auditability are critical for clinical trust and ethical accountability.

Machine learning models such as RF and XGBoost offered marginal performance gains but introduced complexity without substantial improvement in predictive accuracy or calibration.

Therefore, LR was prioritized in interpretive analyses and policy recommendations as the most trustworthy and generalizable framework for understanding adoption behavior.

### Limitations and Future Research

Several limitations should be noted. First, although trust consistently emerged as the strongest determinant, the causal mechanisms underlying trust formation require further longitudinal validation [62,64].

Second, the study focused on adoption intention rather than actual behavioral engagement, leaving room for future longitudinal and experimental studies [65].

Third, despite the use of nested cross-validation and calibration analyses to minimize overfitting, residual model instability cannot be entirely excluded—particularly among HCPs, where the sample size was relatively smaller and calibration plots showed wider error bars. The variability in secondary feature rankings across models indicates that predictors beyond trust should be interpreted with caution. Future research with larger, multicenter samples and external validation using independent datasets will be essential to confirm model robustness and generalizability.

Fourth, our analysis was conducted within China; cross-cultural replication is necessary to test the generalizability of sociotechnical readiness frameworks across differing regulatory and cultural contexts.

Finally, broader ethical issues—such as algorithmic bias, consent transparency, and localized governance—should be systematically integrated into future research agendas to ensure AI systems advance not only efficiency but also justice and inclusion.

### Conclusion

Overall, this study advances the understanding of how trust, literacy, and institutional readiness jointly shape LLM adoption in health care. It establishes empirical evidence that trust functions as the primary mechanism linking technical credibility with social legitimacy. By conceptualizing sociotechnical readiness as the foundation for equitable AI adoption, this research offers both theoretical enrichment and practical direction. Theoretically, it advances existing technology adoption models by embedding ethical trust, literacy, and governance as core constructs of readiness, thereby bridging behavioral and institutional dimensions of digital transformation. Practically, it informs the design and policy development of AI implementation in health care—emphasizing trust-building mechanisms, literacy training, and transparent governance as prerequisites for sustainable integration. This study advances current understanding by shifting the focus from algorithmic performance toward the social, ethical, and institutional factors—particularly trust, literacy, and organizational readiness—that determine equitable AI adoption.

## Supplementary material

10.2196/84918 Multimedia Appendix 1 Good Reporting of A Mixed Methods Study (GRAMMS) reporting summary.

10.2196/84918 Multimedia Appendix 2 Health care professionals’ and patients’ perceptions of large language models, multivariate correlates of large language model adoption, model performance and classification metrics, net reclassification improvement and integrated discrimination improvement comparisons, and calibration metrics across models.

10.2196/84918 Multimedia Appendix 3 Overview of statistical modeling and analytical procedures.

10.2196/84918 Multimedia Appendix 4 Group comparisons of trust, perceived usefulness, and privacy concerns related to large language models.

10.2196/84918 Multimedia Appendix 5 Interaction effects of key predictors on willingness to adopt large language models among health care professionals. (A) Trust in large language models × prior use of large language models. (B) Trust in large language models × artificial intelligence or informatics training. (C) Prior use of large language models × perceived usefulness. (D) Prior use of large language models × privacy concern. (E) Perceived usefulness × privacy concern. (F) Prior use of large language models × clarity on legal responsibility.

10.2196/84918 Multimedia Appendix 6 Comparative clinical utility of large language models’ adoption prediction models based on cumulative incremental accuracy and net reclassification improvement. (A and B) Cumulative incremental accuracy curves across decision thresholds in health care professionals and patients or caregivers, demonstrating relative model performance in identifying true positives. (C and D) Net reclassification improvement analyses for health care professionals and patients or caregivers, highlighting the added value of the random forest model compared to logistic regression and extreme gradient boosting.

10.2196/84918 Multimedia Appendix 7 Calibration assessment of large language models’ adoption prediction models across user groups using Hosmer-Lemeshow plots. (A and B) Hosmer-Lemeshow plots for health care professionals across logistic regression, random forest, and extreme gradient boosting models. (C and D) Hosmer-Lemeshow plots for patients or caregivers across logistic regression, random forest, and extreme gradient boosting models, showing calibration performance and deviations from perfect prediction.

10.2196/84918 Multimedia Appendix 8 Interaction effects of key predictors on willingness to adopt large language models among patients or caregivers. (A) Trust in large language models × prior use of large language models. (B) Trust in large language models × prior awareness of large language models. (C) Trust in large language models × low education level (Primary school or below). (D) Prior use of large language models × perceived usefulness. (E) Prior use of large language models × privacy concern. (F) Privacy concern × education level (high school).

10.2196/84918 Multimedia Appendix 9 Sensitivity analysis of predictor perturbations in health care professionals. (A-C) Probability distribution shifts in logistic regression, random forest, and extreme gradient boosting models when trust in large language models is perturbed (D-F). Heatmaps of model performance changes (ΔProba, ΔAUC, ΔLogLoss) across top predictors.

10.2196/84918 Multimedia Appendix 10 Sensitivity analysis of predictor perturbations in patients or caregivers. (A-C) Probability distribution shifts in logistic regression, random forest, and extreme gradient boosting models when trust in large language models is perturbed. (D-F) Heatmaps of model performance changes (ΔProba, ΔAUC, ΔLogLoss) across top predictors.

10.2196/84918 Multimedia Appendix 11 Decision curve analysis comparing clinical utility of predictive models for large language models adoption across user groups. (A) Net benefit curves for logistic regression, random forest, and extreme gradient boosting in health care professionals. (B) Net benefit curves for the same models in patients or caregivers.

10.2196/84918 Multimedia Appendix 12 GPT-5 Interaction summary.

## Abbreviations

AI: artificial intelligence
AUC: area under the receiver operating characteristic curve
GRAMMS: Good Reporting of A Mixed Methods Study
LLM: large language model
LR: logistic regression
OR: odds ratio
RF: random forest
SHAP: Shapley additive explanations
XGBoost: extreme gradient boosting
