# Supplementary material for: Factors Influencing Adoption of Large Language Models in Health Care: Multicenter Cross-Sectional Mixed Methods Observational Study
Source: J Med Internet Res. 2025 Dec 11;27:e84918. doi: 10.2196/84918 (PMC12697921; doi:10.2196/84918)
Supplement: Multimedia Appendix 1 [file jmir-v27-e84918-s001.pdf]

Supplementary File – GRAMMS Reporting Summary

Reference: O'Cathain A, et al. *The quality of mixed methods studies in health services research.* J Health Serv Res Policy. 2008;13(2):92–98.

This study followed the **Good Reporting of A Mixed Methods Study (GRAMMS)** guideline.

The six core principles and how they were addressed in this manuscript are summarized below:

GRAMMS Domain	How Addressed in This Study
**1. Justification for using mixed methods**	Mixed methods were adopted to comprehensively explore factors influencing LLM adoption, combining quantitative predictors (trust, usefulness, literacy) with qualitative perspectives (workflow, communication, ethics). Reported in *Introduction* and *Methods → Study Design and Setting*.
**2. Description of design**	The study used a parallel convergent design, integrating quantitative survey data and qualitative interviews collected concurrently. Described in *Methods → Study Design and Setting*.
**3. Description of each component**	Quantitative and qualitative methods are detailed in *Methods → Assessments and Data Sources* and *Methods → Qualitative Interviews*.
**4. Integration of components**	Integration occurred at the interpretation level through joint discussion of convergent and divergent findings (*Discussion → Qualitative Integration and Thematic Insights*).
**5. Interpretation of the integrated findings**	Integration emphasized how qualitative findings explained and contextualized quantitative predictors (*Discussion* section).
**6. Limitations of the mixed-methods approach**	Discussed in *Limitations and Future Research*, including potential imbalance between components and sample size limitations.
