# Supplementary material for: Factors Influencing Adoption of Large Language Models in Health Care: Multicenter Cross-Sectional Mixed Methods Observational Study
Source: J Med Internet Res. 2025 Dec 11;27:e84918. doi: 10.2196/84918 (PMC12697921; doi:10.2196/84918)
Supplement: Multimedia Appendix 2 [file jmir-v27-e84918-s002.docx]

**Table S1.** Health care professionals’ perceptions and acceptance of large language models in medical settings.

| **A. Demographics** |
| --- |
| Hospital (Single choice) |
| Department (Internal Medicine / Surgery / ICU / Emergency / Other) |
| Profession (Physician / Nurse) |
| Gender (Male / Female / Other) |
| Age (numeric input) |
| Years of clinical experience (numeric input) |
| **B. Awareness and Use of LLMs** |
| 1. Have you heard of ChatGPT, Wenxin Yiyan, DeepSeek, or similar LLM tools? (Yes/No) |
| 1. Have you ever used or attempted to use LLM tools in your clinical work? (Yes/No) |
| **C. Attitudes Toward LLMs (5-point Likert scale: Strongly Disagree to Strongly Agree)** |
| 1. I am willing to use LLMs as an aid in clinical decision-making. |
| 1. I believe LLMs can improve my work efficiency. |
| 1. I believe LLMs provide reliable suggestions in most cases. |
| 1. I clearly understand who is legally responsible when using LLMs. |
| 1. I am concerned that LLMs may lead to patient privacy breaches. |
| 1. I have a positive attitude towards future use of LLMs in hospitals. |
| **D. Optional Open-Ended Questions** |
| 1. What is your biggest concern when using or learning about LLMs? |
| 1. In which clinical scenarios do you think LLMs are most applicable? |

**Table S2.** Patients’ and caregivers’ perceptions and acceptance of large language models in medical settings.

| **A. Demographics** |
| --- |
| Hospital (Single choice) |
| Role (Patient / Caregiver) |
| Gender (Male / Female / Other) |
| Age (numeric input) |
| Education Level (Primary or below / Junior high / High school / College / Bachelor or above) |
| Frequency of digital tool use (Often / Occasionally / Rarely) |
| **B. Awareness and Willingness to Use LLMs** |
| 1. Have you heard of ChatGPT, AI doctors, or intelligent medical assistants? (Yes/No) |
| 1. Would you mind if your doctor uses AI tools to help explain your condition? (Yes/No/Uncertain) |
| **C. Attitudes Toward LLMs (5-point Likert scale: Strongly Disagree to Strongly Agree)** |
| 1. I am willing to receive explanations of my condition or test results from an LLM. |
| 1. I find explanations by AI easier to understand than those by doctors. |
| 1. I trust that AI will not provide incorrect or misleading information. |
| 1. I am concerned about privacy issues when AI uses my information. |
| 1. I would be more willing to use AI tools if the service is free. |
| **D. Optional Open-Ended Questions** |
| 1. What kind of information would you most like AI to explain when used in a hospital setting? |

**Table S3**. Multivariate correlations of large language models’ adoption among health care professionals.

| Variable | OR | 95% CI | *P* value |
| --- | --- | --- | --- |
| **Intercept** | - | - | 0.002 |
| Gender: Male vs. Female | 1.83 | [0.76, 4.41] | 0.177 |
| Profession: Physician vs. Nurse/Others | 0.83 | [0.32, 2.15] | 0.698 |
| Department: ICU vs. ER | 2.55 | [0.65, 10.04] | 0.180 |
| Department: Internal vs. ER | 1.26 | [0.34, 4.64] | 0.733 |
| Department: Other vs. ER | 1.89 | [0.54, 6.64] | 0.323 |
| Department: Surgery vs. ER | 1.85 | [0.50, 6.91] | 0.360 |
| Hospital affiliation: Non-affiliated vs. Affiliated | 1.72 | [0.72, 4.12] | 0.225 |
| Hospital type: Oncology vs. General | 0.80 | [0.26, 2.46] | 0.702 |
| Province economic level: Low vs. High | **0.11** | **[0.03, 0.42]** | **0.001** |
| Province economic level: Medium vs. High | **0.21** | **[0.08, 0.57]** | **0.002** |
| Frequency of digital tool use: Often vs. Never | 1.58 | [0.59, 4.23] | 0.367 |
| Frequency of digital tool use: Rarely vs. Never | **0.13** | **[0.03, 0.61]** | **0.010** |
| Prior awareness of LLMs: Yes vs. No | 1.6 | [0.60, 4.27] | 0.348 |
| Prior use of LLMs: Yes vs. No | **5.61** | **[1.99, 15.76]** | **0.001** |
| Received AI/informatics training: Yes vs. No | 2.11 | [0.85, 5.25] | 0.109 |
| Age | 1.00 | [0.93, 1.08] | 0.989 |
| Years of clinical experience | **1.13** | **[1.02, 1.25]** | **0.017** |
| Perceived usefulness of LLMs | **1.49** | **[1.04, 2.15]** | **0.030** |
| Trust in LLMs | **3.78** | **[2.40, 5.95]** | **<0.001** |
| Privacy concern | **0.50** | **[0.34, 0.75]** | **<0.001** |
| Clarity on legal responsibility | **1.56** | **[1.07, 2.27]** | **0.021** |

**Table S4**. Multivariate correlates of large language models’ adoption among patients or caregivers.

| Variable | OR | 95% CI | *P* value |
| --- | --- | --- | --- |
| **Intercept** | - | - | <0.001 |
| Gender: Male vs. Female | 0.82 | [0.36, 1.86] | 0.634 |
| Role: Patient vs. Caregiver | 0.79 | [0.31, 1.99] | 0.614 |
| Education: College vs. Bachelor+ | 1.59 | [0.39, 6.42] | 0.517 |
| Education: High school vs. Bachelor+ | 0.36 | [0.09, 1.46] | 0.152 |
| Education: Junior high vs. Bachelor+ | **0.18** | **[0.04, 0.75]** | **0.019** |
| Education: Primary or below vs. Bachelor+ | **0.03** | **[0.01, 0.18]** | **<0.001** |
| Hospital affiliation: Non-affiliated vs. Affiliated | 0.74 | [0.32, 1.74] | 0.492 |
| Hospital type: Oncology vs. General | 1.51 | [0.47, 4.90] | 0.489 |
| Province economic level: Low vs. High | **0.15** | **[0.04, 0.56]** | **0.005** |
| Province economic level: Medium vs. High | **0.27** | **[0.09, 0.84]** | **0.023** |
| Frequency of digital tool use: Often vs. Never | 0.42 | [0.15, 1.20] | 0.104 |
| Frequency of digital tool use: Rarely vs. Never | **0.009** | **[0.001, 0.057]** | **<0.001** |
| Prior awareness of LLMs: Yes vs. No | 1.42 | [0.64, 3.15] | 0.394 |
| Prior use of LLMs: Yes vs. No | 1.31 | [0.52, 3.29] | 0.562 |
| Age (continuous) | 1.01 | [0.98, 1.05] | 0.397 |
| Perceived usefulness of LLMs | **2.01** | **[1.34, 3.03]** | **<0.001** |
| Trust in LLMs | **36.34** | **[15.28, 86.38]** | **<0.001** |
| Privacy concern | **0.33** | **[0.22, 0.49]** | **<0.001** |

**Table S5**. Model performance metrics for large language models’ adoption (health care professionals and patients or caregivers).

| Model | Train AUC | Test AUC | CV AUC | Train Brier Score | Test Brier Score | CV Brier Score |
| --- | --- | --- | --- | --- | --- | --- |
| **Healthcare Professionals (HCPs)** | | | | | | |
| Logistic Regression | 0.97 | 0.83 | 0.93 | 0.07 | 0.17 | 0.11 |
| XGBoost | 0.95 | 0.83 | 0.87 | 0.12 | 0.18 | 0.16 |
| Random Forest | 0.97 | 0.85 | 0.91 | 0.12 | 0.18 | 0.16 |
| **Patients/Caregivers (PCs)** | | | | | | |
| Logistic Regression | 0.99 | 0.95 | 0.97 | 0.04 | 0.09 | 0.06 |
| XGBoost | 0.97 | 0.94 | 0.96 | 0.08 | 0.10 | 0.09 |
| Random Forest | 0.98 | 0.96 | 0.95 | 0.08 | 0.01 | 0.10 |

**Table S6**. Predictive classification metrics for large language model adoption (health care professionals and patients or caregivers).

| Model |  |  |  |  |
| --- | --- | --- | --- | --- |
| **Healthcare Professionals (HCPs)** | F1 Score | F1 95% CI | Precision | Precision 95% CI |
| Logistic Regression | 0.77 | [0.64, 0.88] | 0.77 | [0.62, 0.91] |
| XGBoost | 0.78 | [0.67, 0.88] | 0.71 | [0.58, 0.85] |
| Random Forest | 0.76 | [0.64, 0.85] | 0.72 | [0.57, 0.86] |
|  | Recall | Recall 95% CI | Accuracy | Accuracy 95% CI |
| Logistic Regression | 0.77 | [0.61, 0.91] | 0.78 | [0.68, 0.88] |
| XGBoost | 0.86 | [0.74, 0.97] | 0.76 | [0.67, 0.86] |
| Random Forest | 0.80 | [0.67, 0.92] | 0.75 | [0.65, 0.85] |
|  |  |  |  |  |
| **Patients/Caregivers (PCs)** | F1 Score | F1 95% CI | Precision | Precision 95% CI |
| Logistic Regression | 0.83 | [0.74, 0.89] | 0.81 | [0.71, 0.90] |
| XGBoost | 0.79 | [0.71, 0.86] | 0.78 | [0.67, 0.88] |
| Random Forest | 0.87 | [0.80, 0.93] | 0.90 | [0.81, 0.98] |
|  | Recall | Recall 95% CI | Accuracy | Accuracy 95% CI |
| Logistic Regression | 0.84 | [0.74, 0.93] | 0.86 | [0.81, 0.92] |
| XGBoost | 0.80 | [0.70, 0.90] | 0.83 | [0.78, 0.89] |
| Random Forest | 0.84 | [0.74, 0.93] | 0.90 | [0.85, 0.95] |

**Table S7**. NRI and IDI for pairwise model comparisons in HCPs and PCs.

| Model_1 | Model_2 | NRI_Total | NRI_Event | NRI_NonEvent | IDI |
| --- | --- | --- | --- | --- | --- |
| **Healthcare Professionals (HCPs)** | | | | | |
| Logistic | XGBoost | -0.022 | 0.086 | -0.108 | -0.253 |
| Logistic | RandomForest | -0.053 | 0.029 | -0.081 | -0.268 |
| XGBoost | RandomForest | -0.030 | -0.058 | 0.027 | -0.016 |
| **Patients/Caregivers (PCs)** | | | | | |
| Logistic | XGBoost | -0.058 | -0.036 | -0.023 | -0.193 |
| Logistic | RandomForest | 0.068 | 0 | 0.068 | -0.215 |
| XGBoost | RandomForest | 0.127 | 0.036 | 0.091 | -0.022 |

**Table S8**. Calibration metrics across models for HCPs and PCs.

| Model | ECE | MCE | Calibration_Intercept | Calibration_Slope |
| --- | --- | --- | --- | --- |
| **Healthcare Professionals (HCPs)** | | | | |
| Logistic Regression | 0.15 | 0.54 | -0.17 | 0.45 |
| XGBoost | 0.11 | 0.25 | -0.44 | 1.73 |
| Random Forest | 0.15 | 0.28 | -0.32 | 2.24 |
| **Patients/Caregivers (PCs)** | | | | |
| Logistic Regression | 0.04 | 0.29 | -0.13 | 0.89 |
| XGBoost | 0.14 | 0.43 | -0.12 | 1.8 |
| Random Forest | 0.15 | 0.23 | 0.05 | 2.59 |
