# Supplementary figures and images for: Factors Influencing Adoption of Large Language Models in Health Care: Multicenter Cross-Sectional Mixed Methods Observational Study
Source: J Med Internet Res. 2025 Dec 11;27:e84918. doi: 10.2196/84918 (PMC12697921; doi:10.2196/84918)

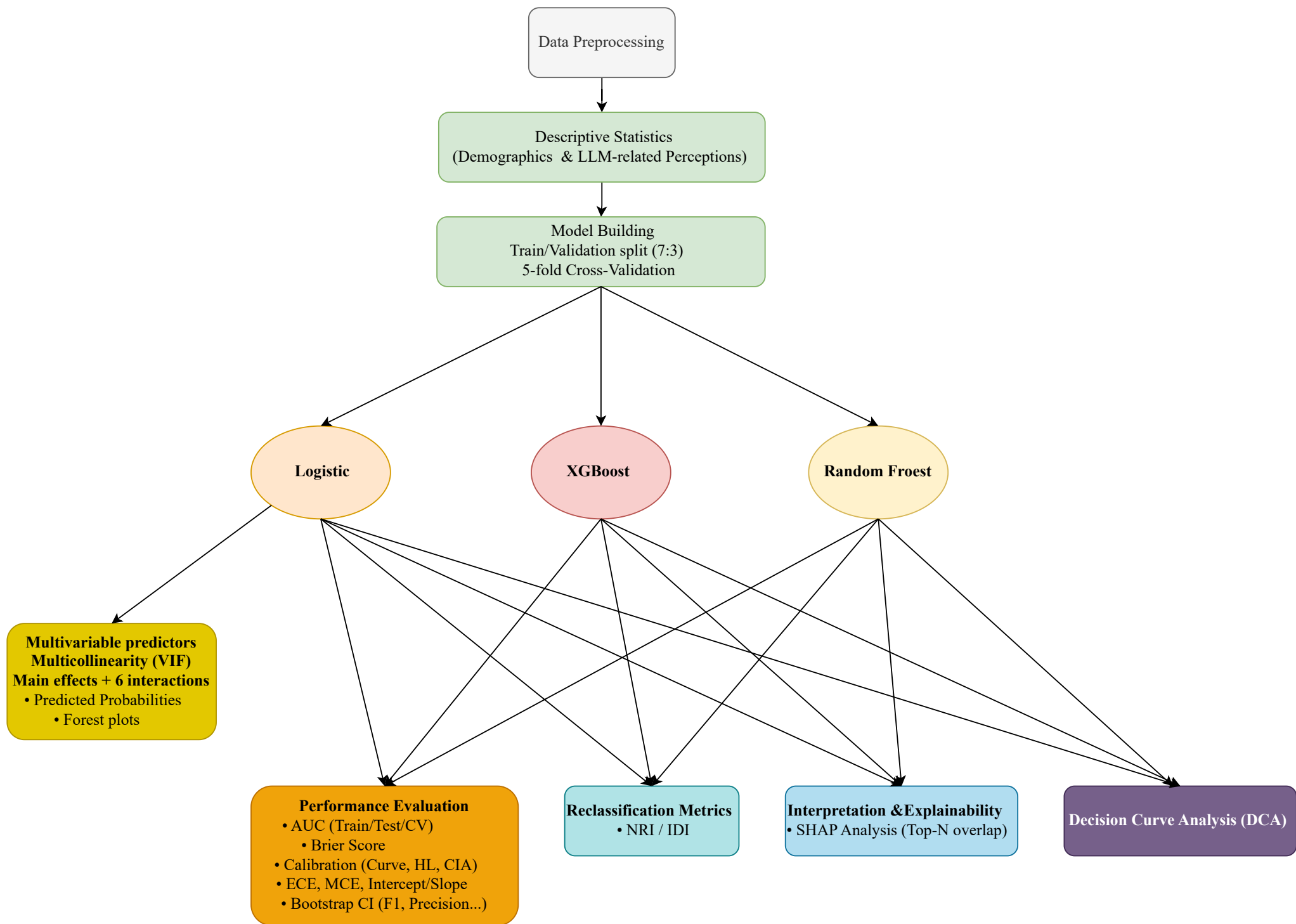

Supplement: Multimedia Appendix 3 [file jmir-v27-e84918-s003.pdf]

Perceptions of LLMs by Group

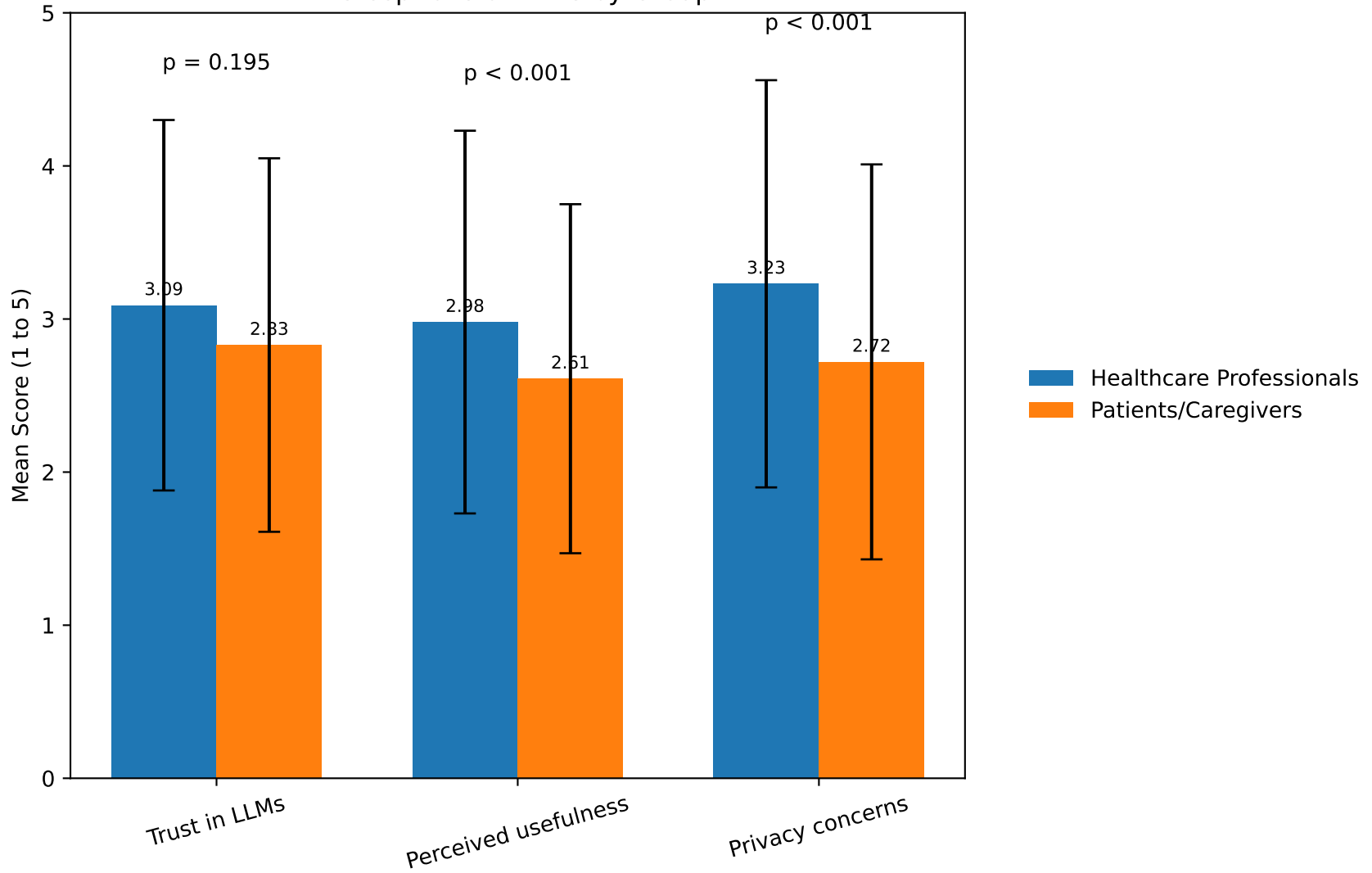

Supplement: Multimedia Appendix 4 [file jmir-v27-e84918-s004.pdf]

**A**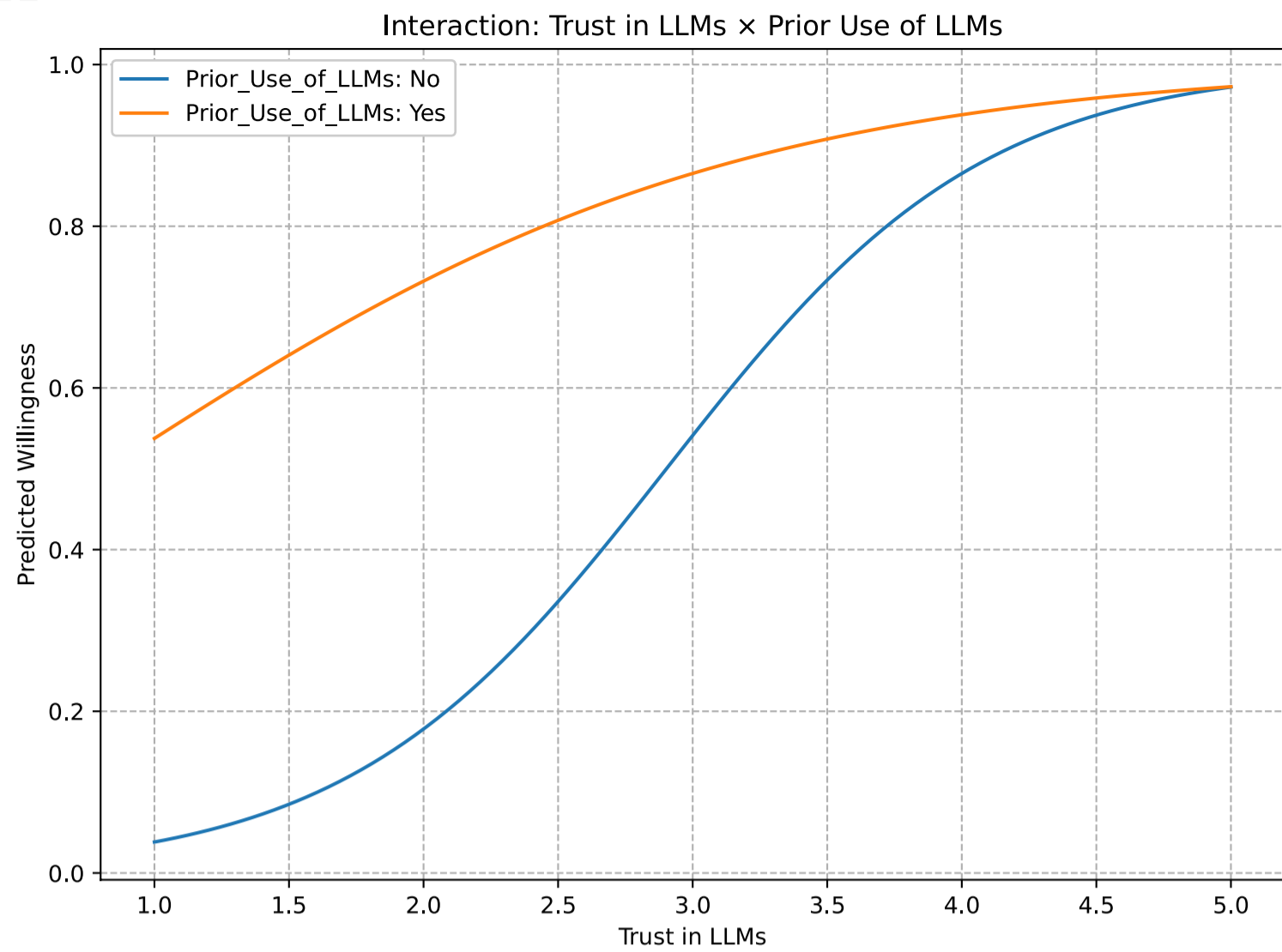**B**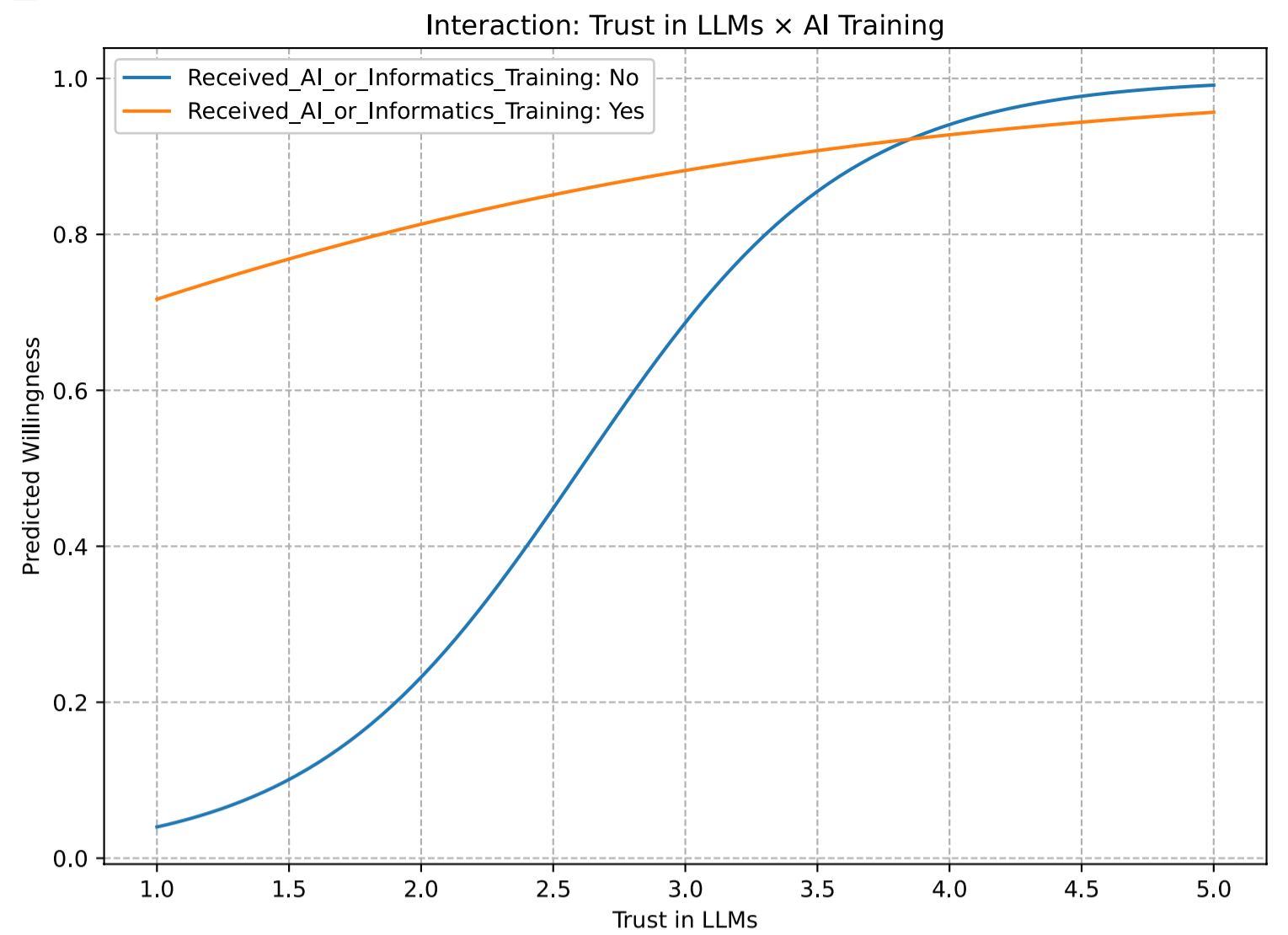**C**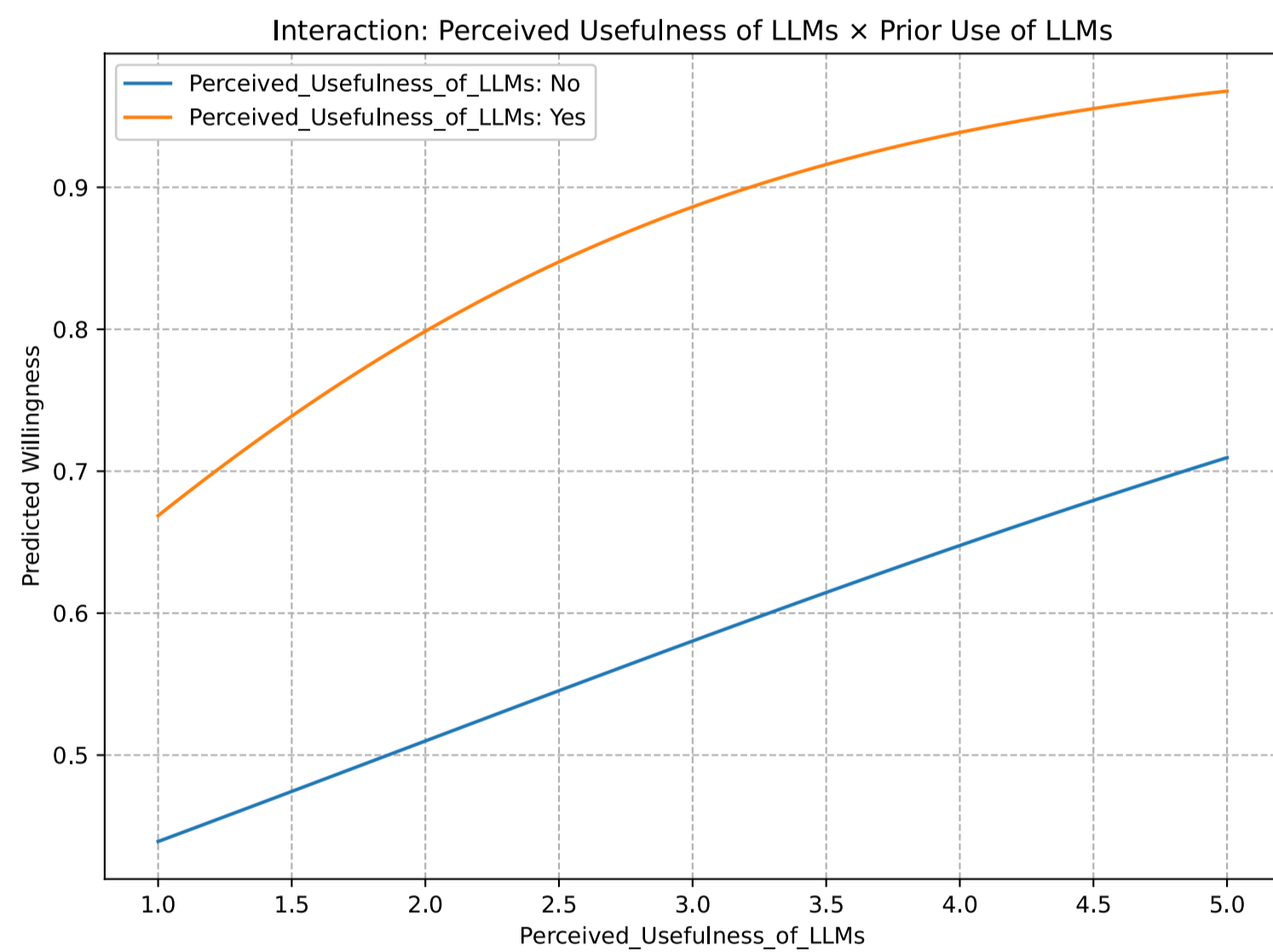**D**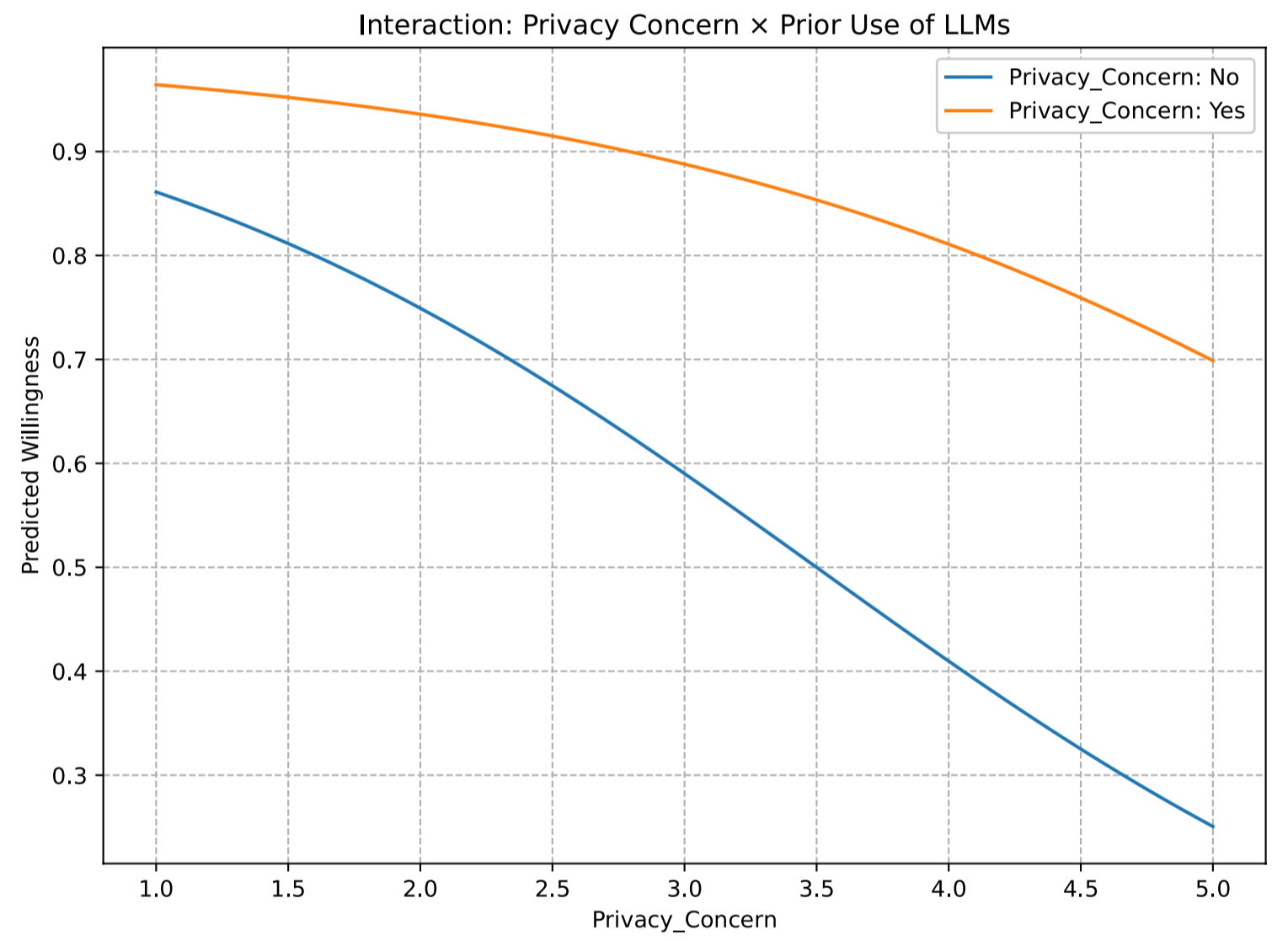**E**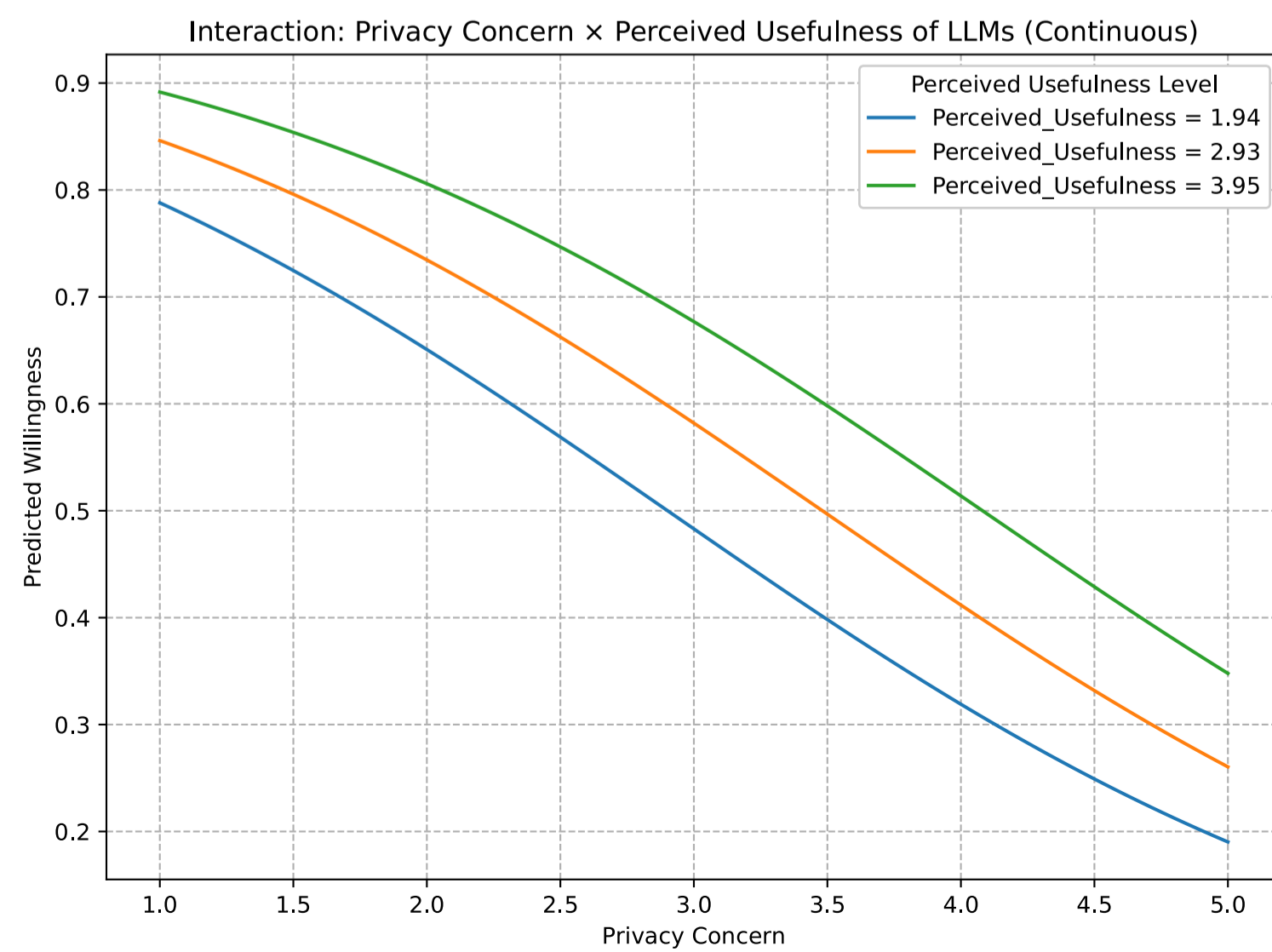**F**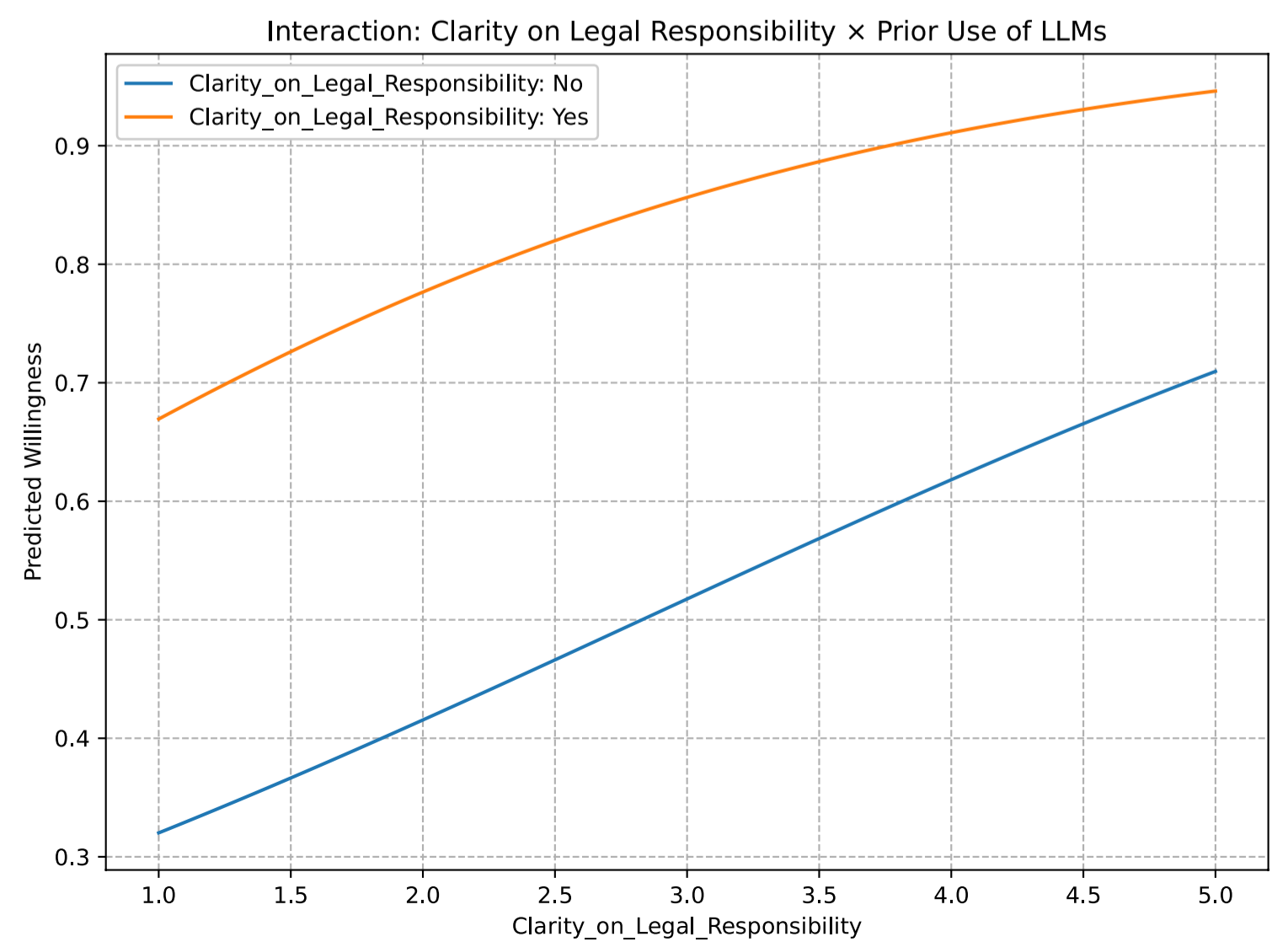

Supplement: Multimedia Appendix 5 [file jmir-v27-e84918-s005.pdf]

**A**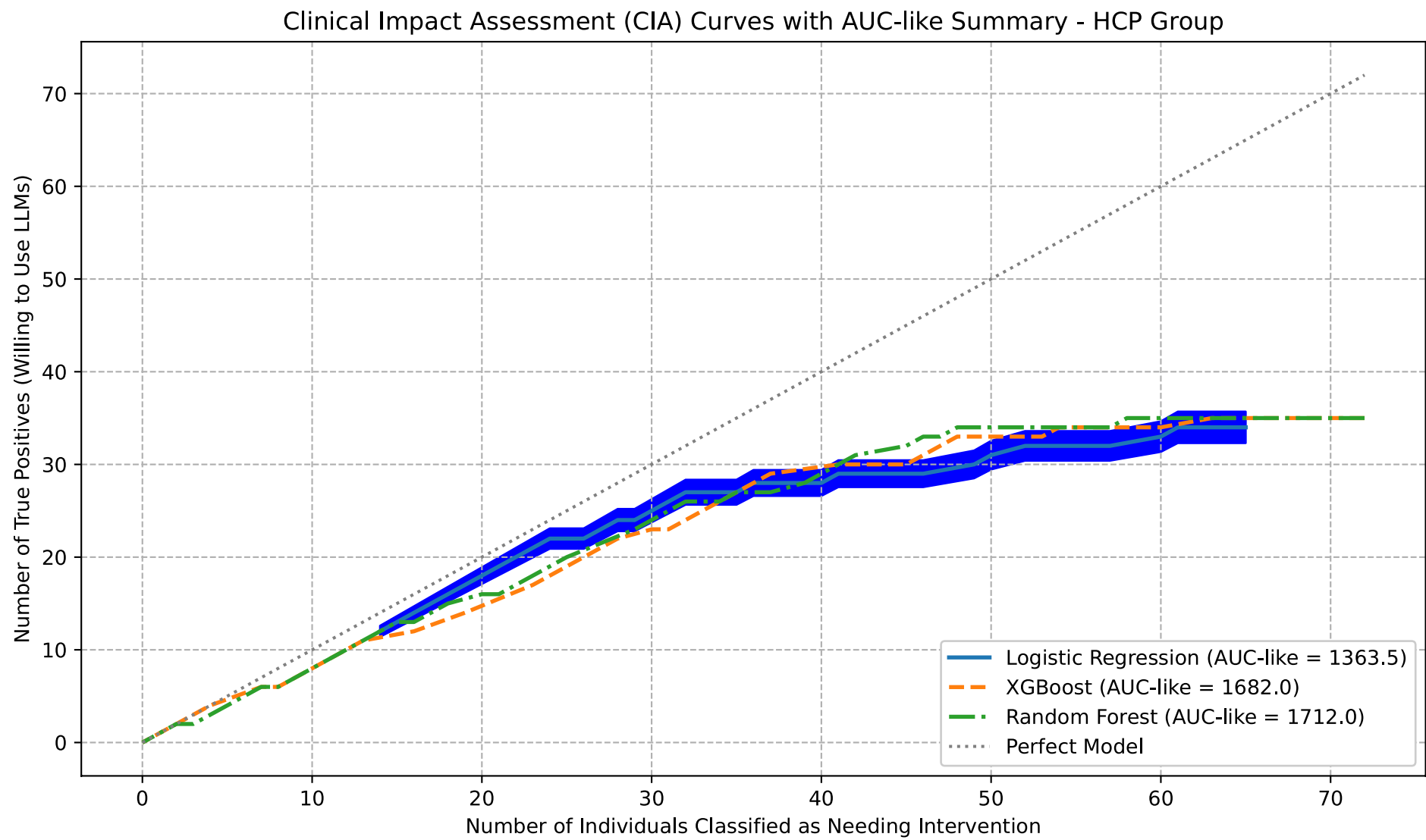**B**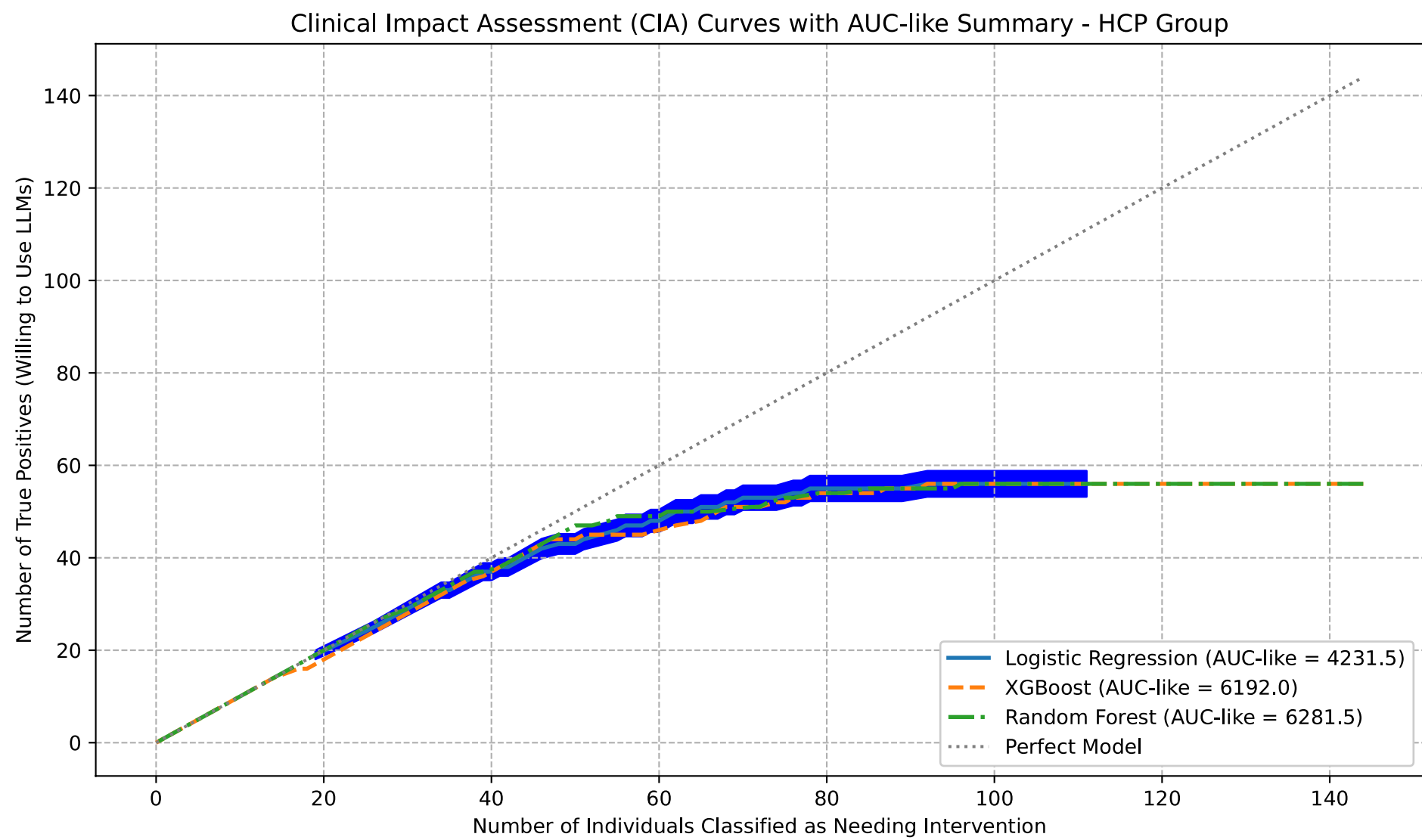**C**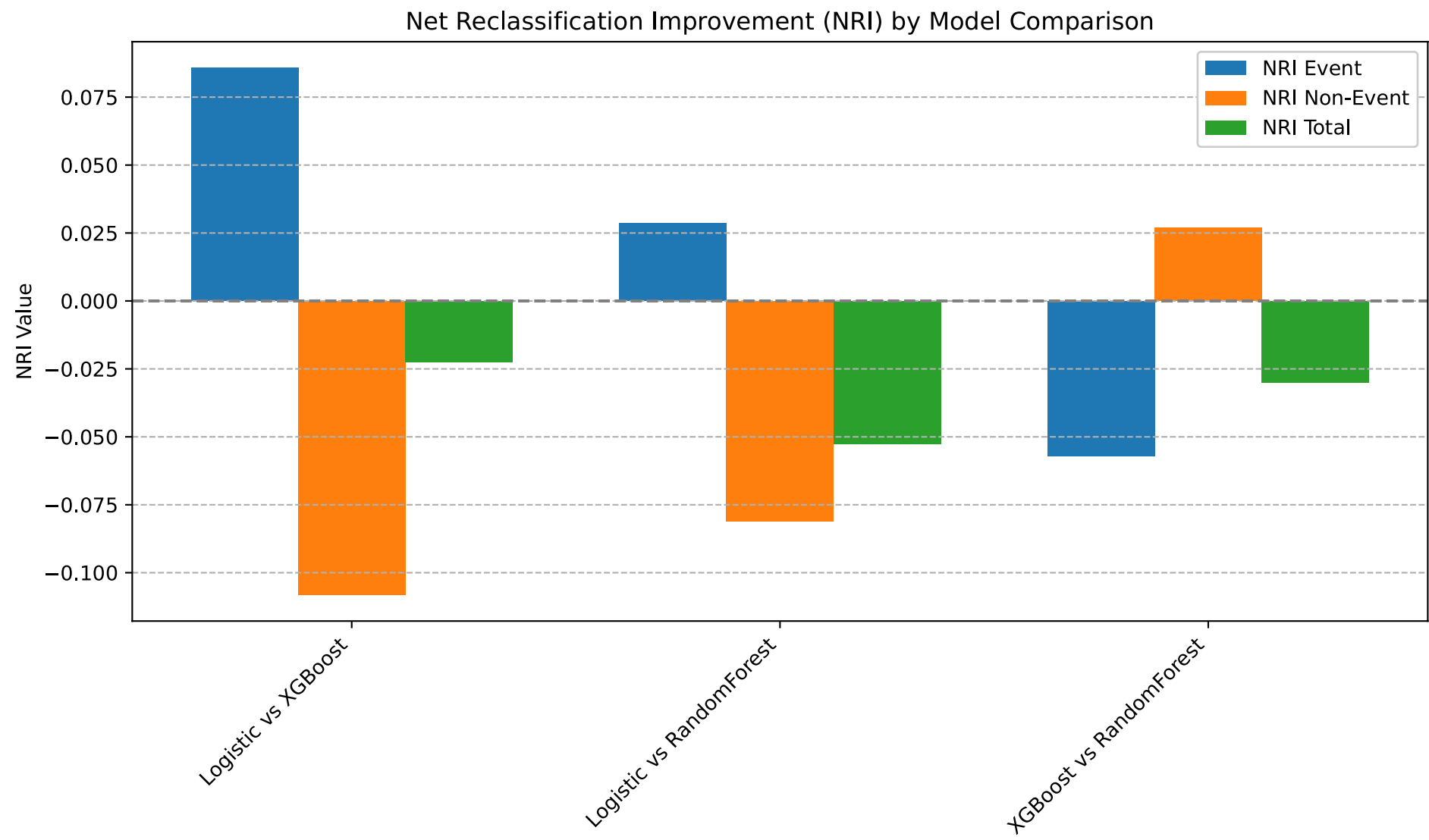**D**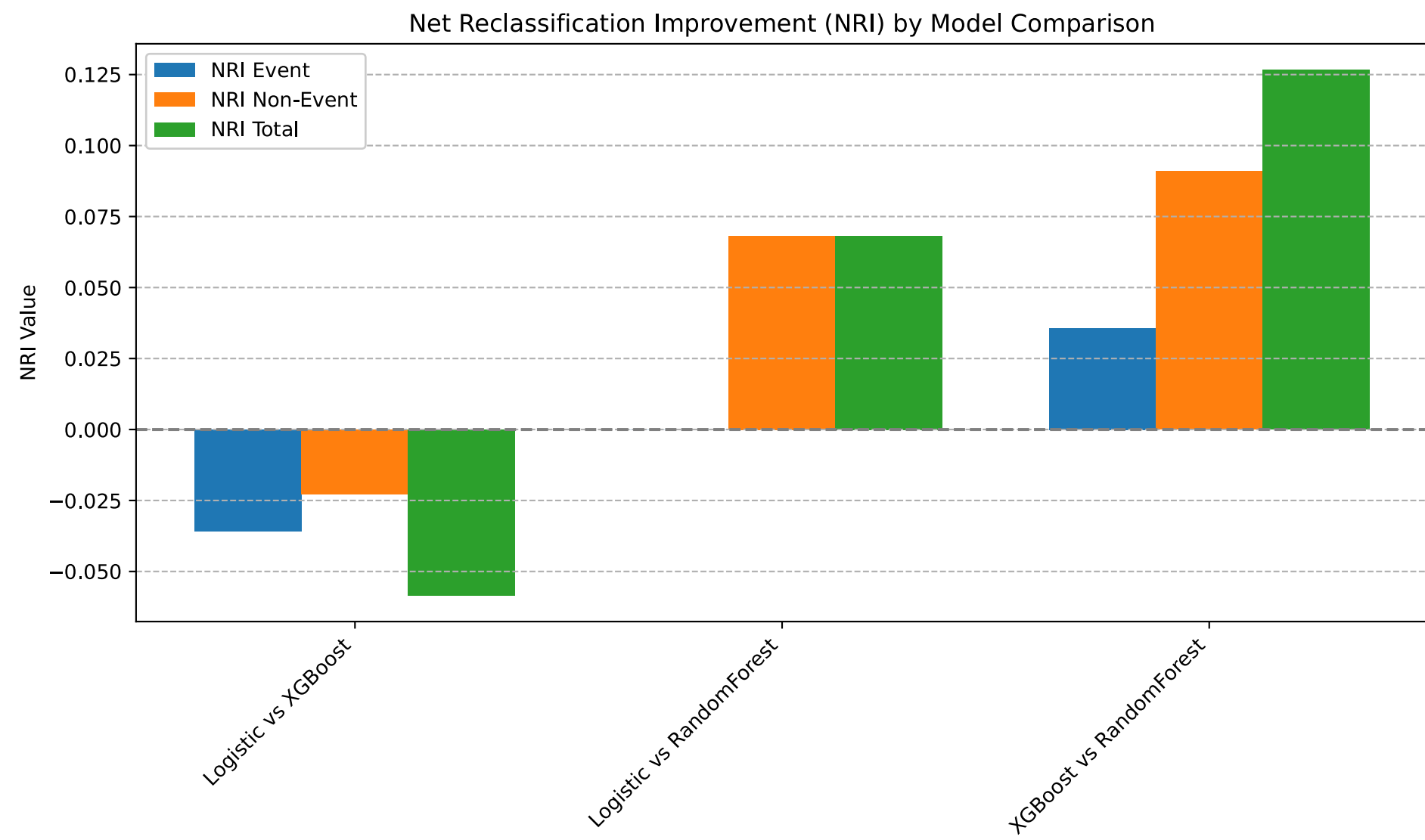

Supplement: Multimedia Appendix 6 [file jmir-v27-e84918-s006.pdf]

A

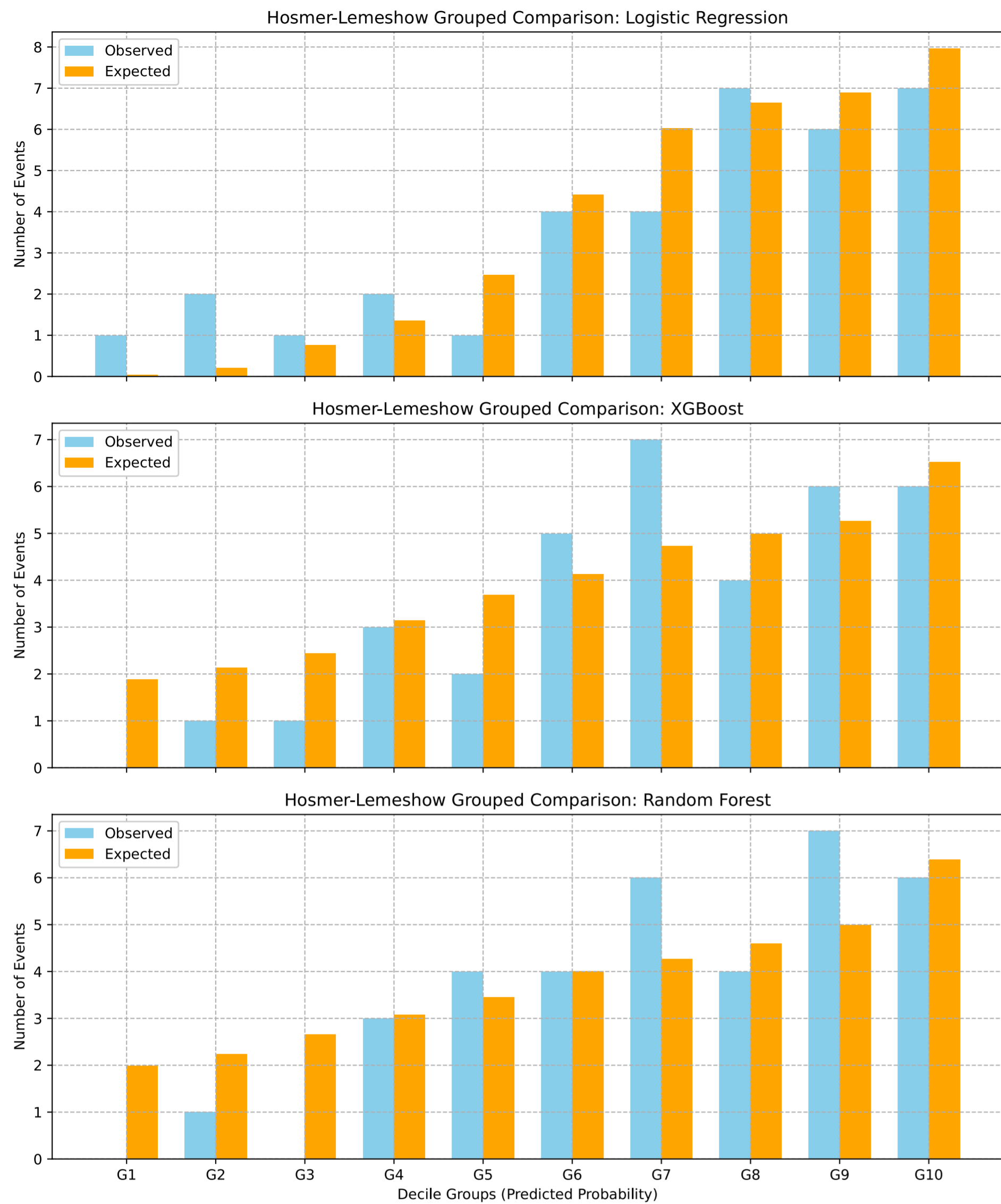

B

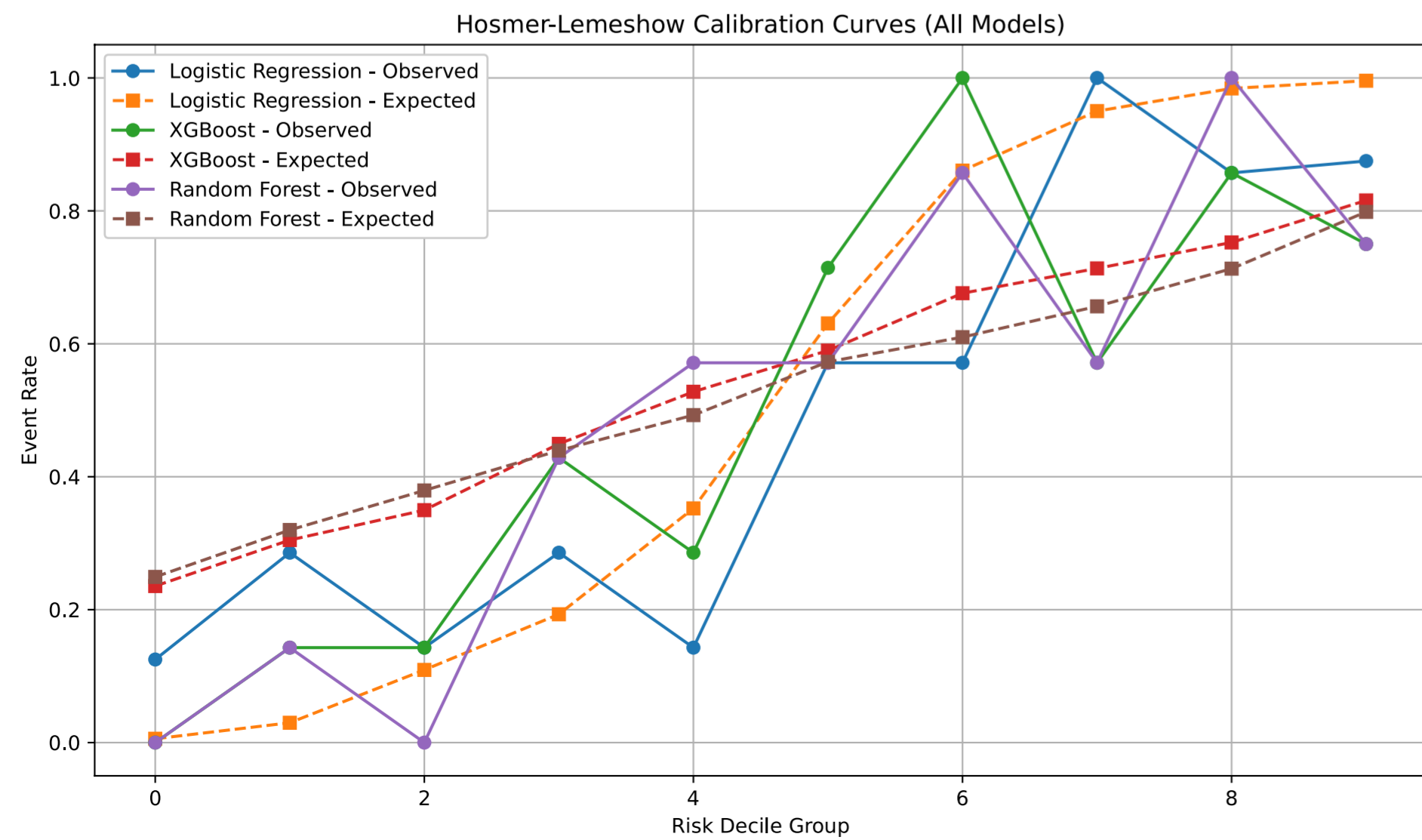

C

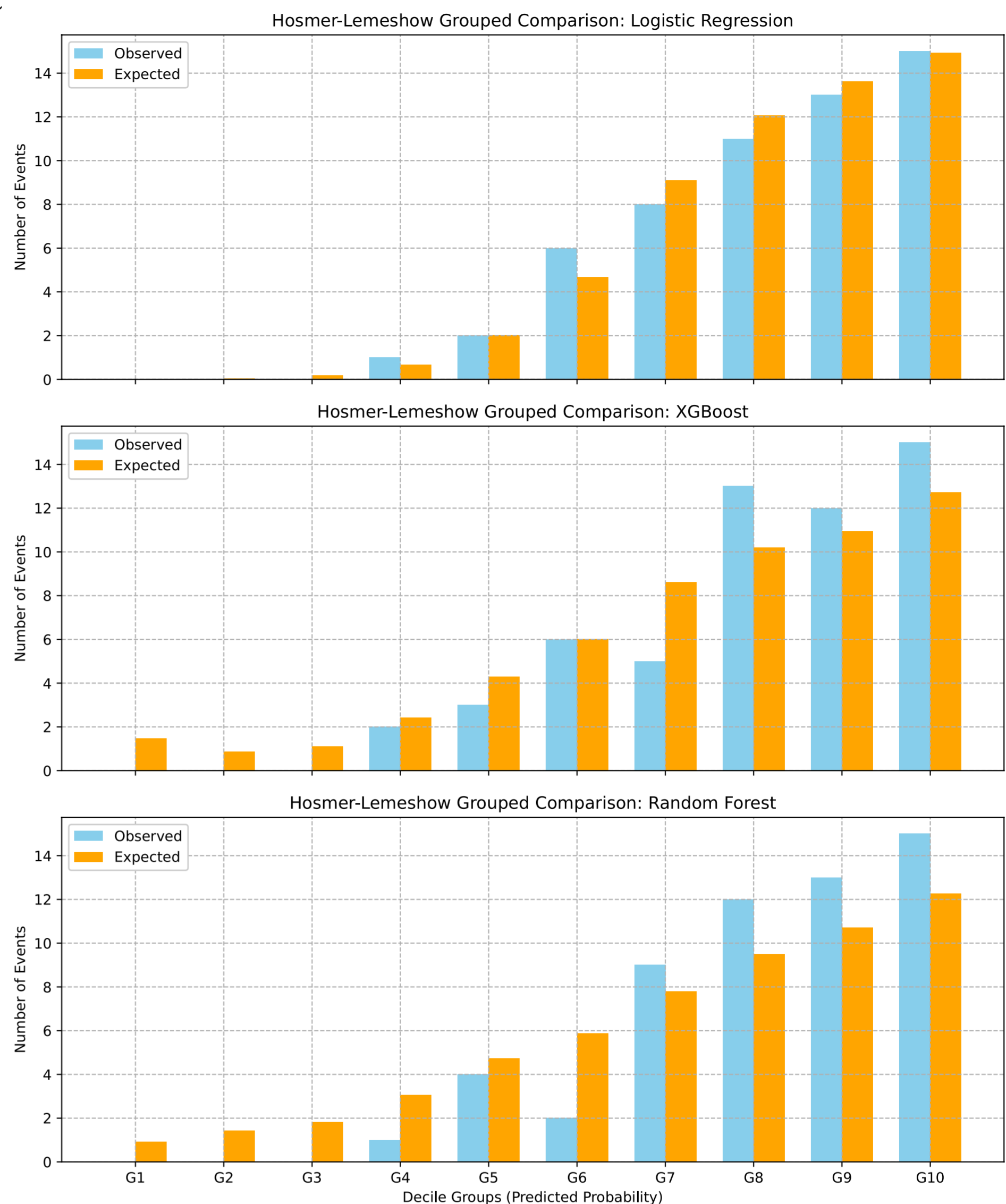

D

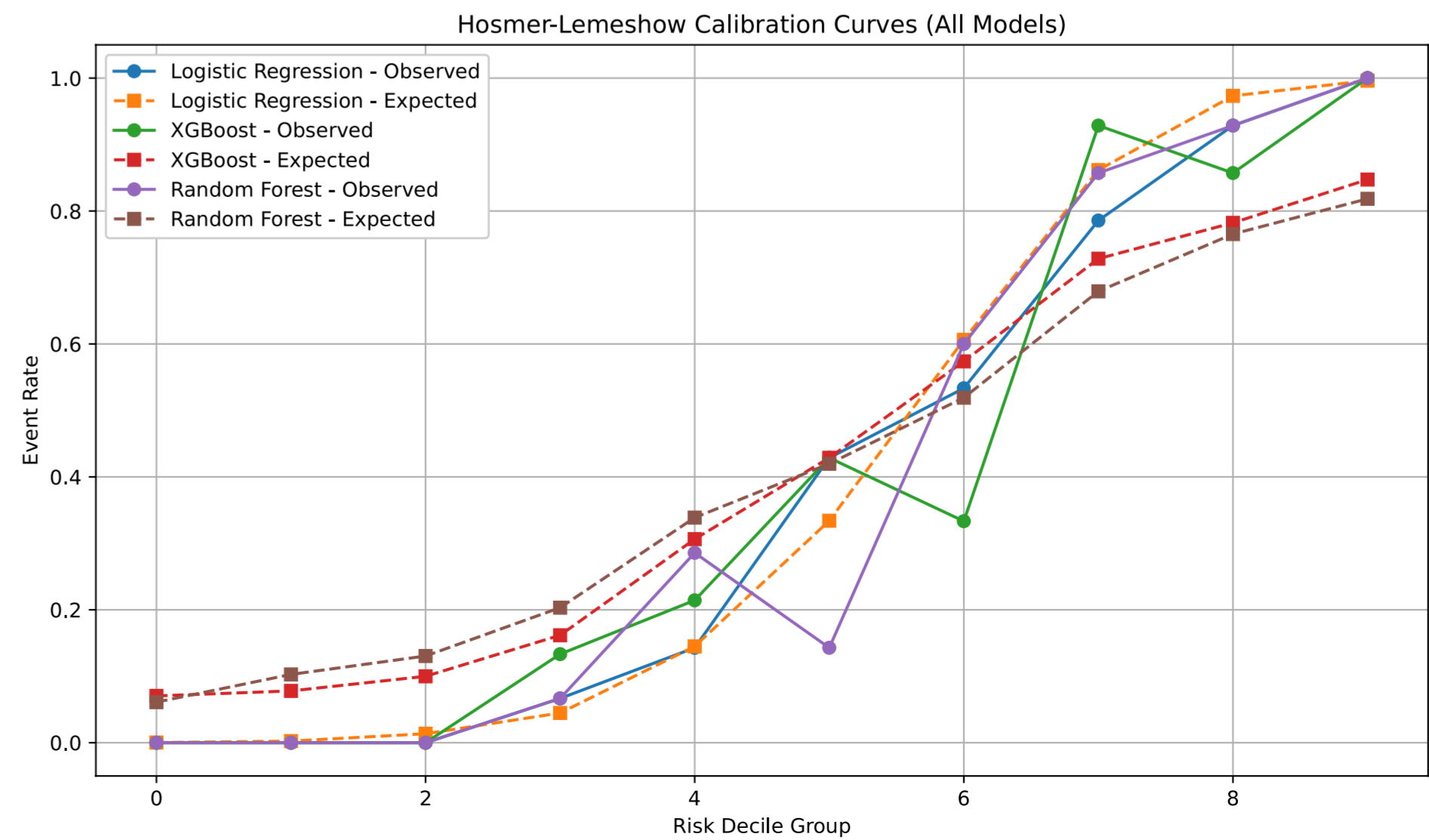

Supplement: Multimedia Appendix 7 [file jmir-v27-e84918-s007.pdf]

**A**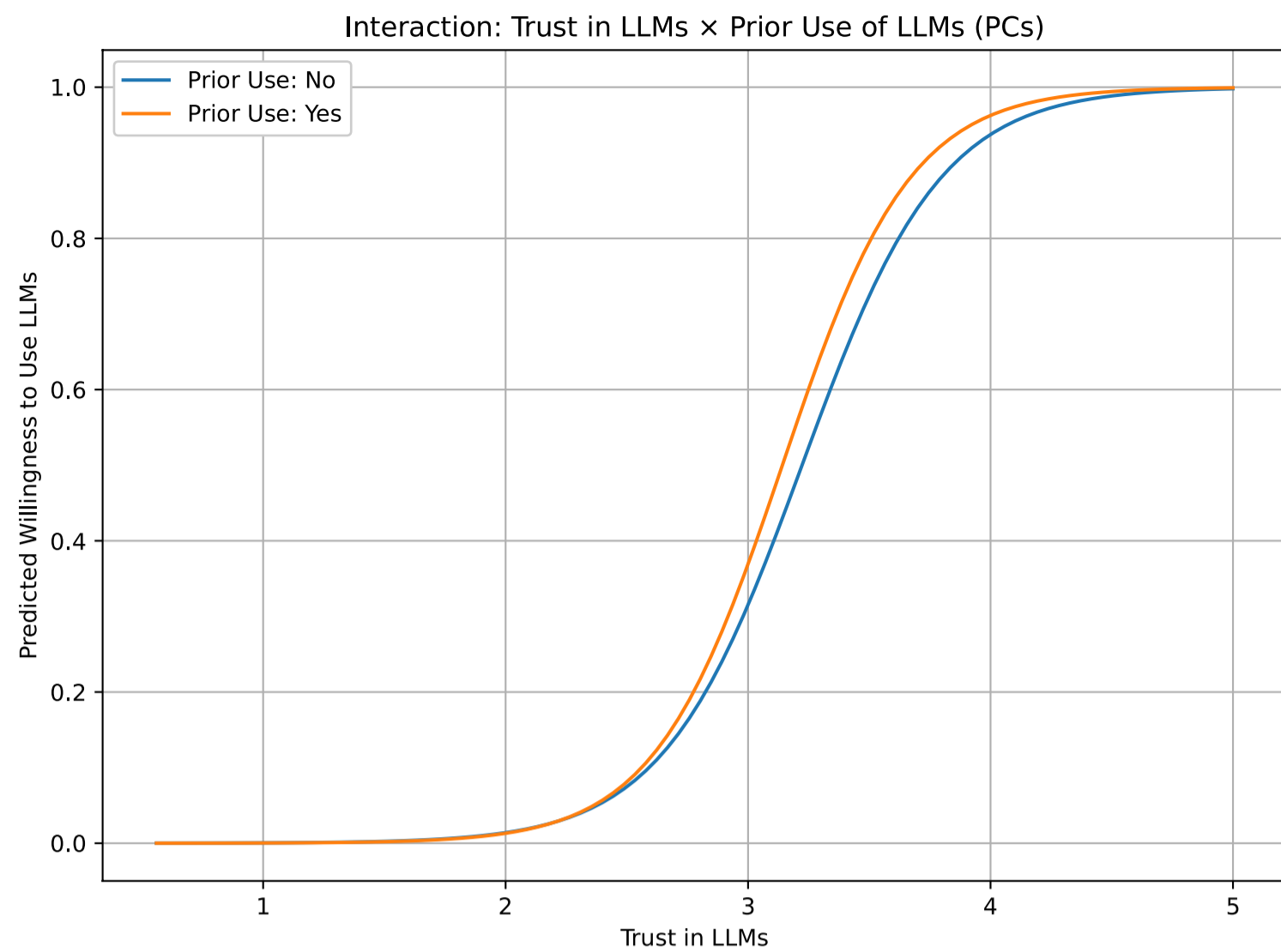**B**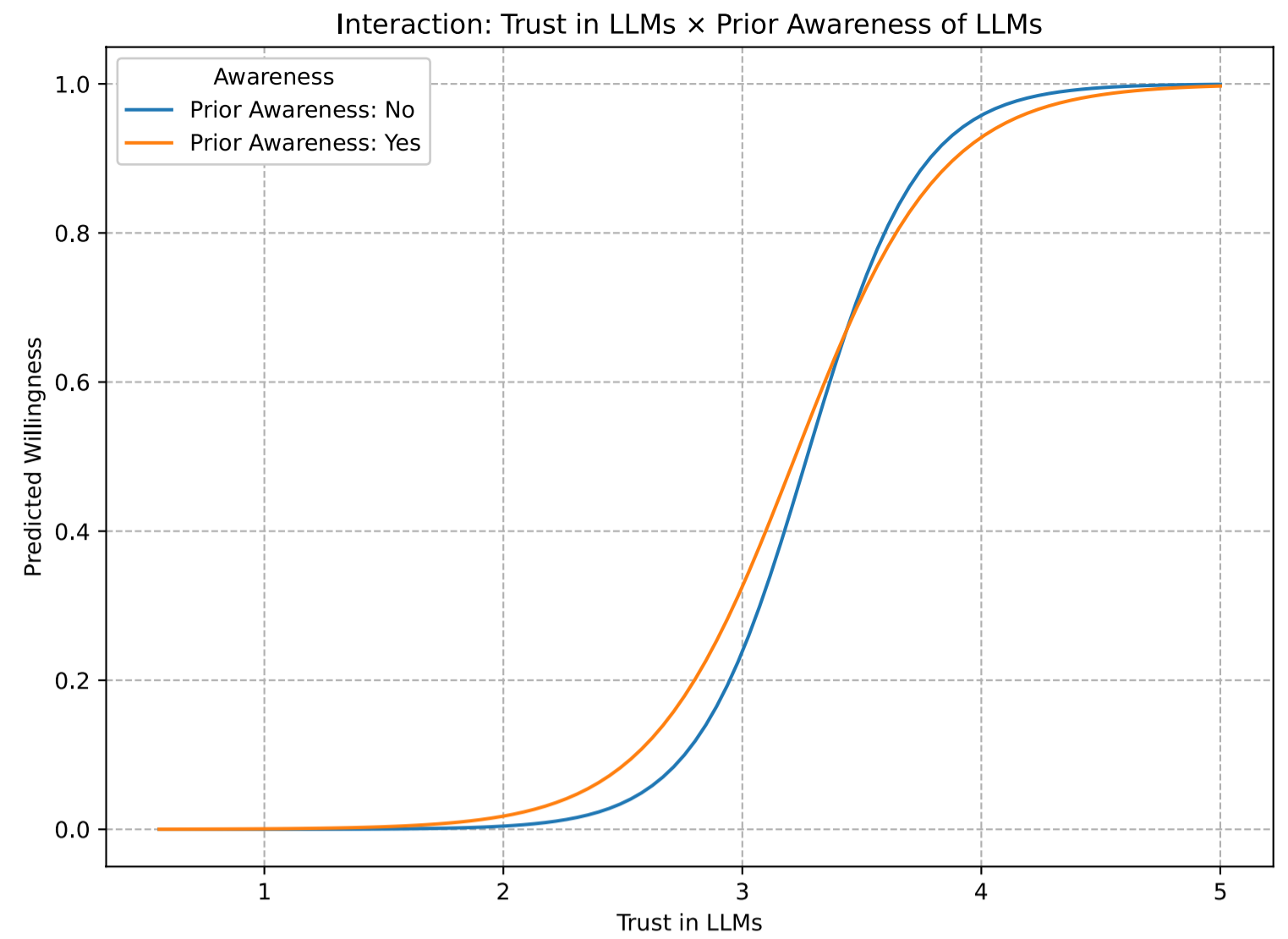**C**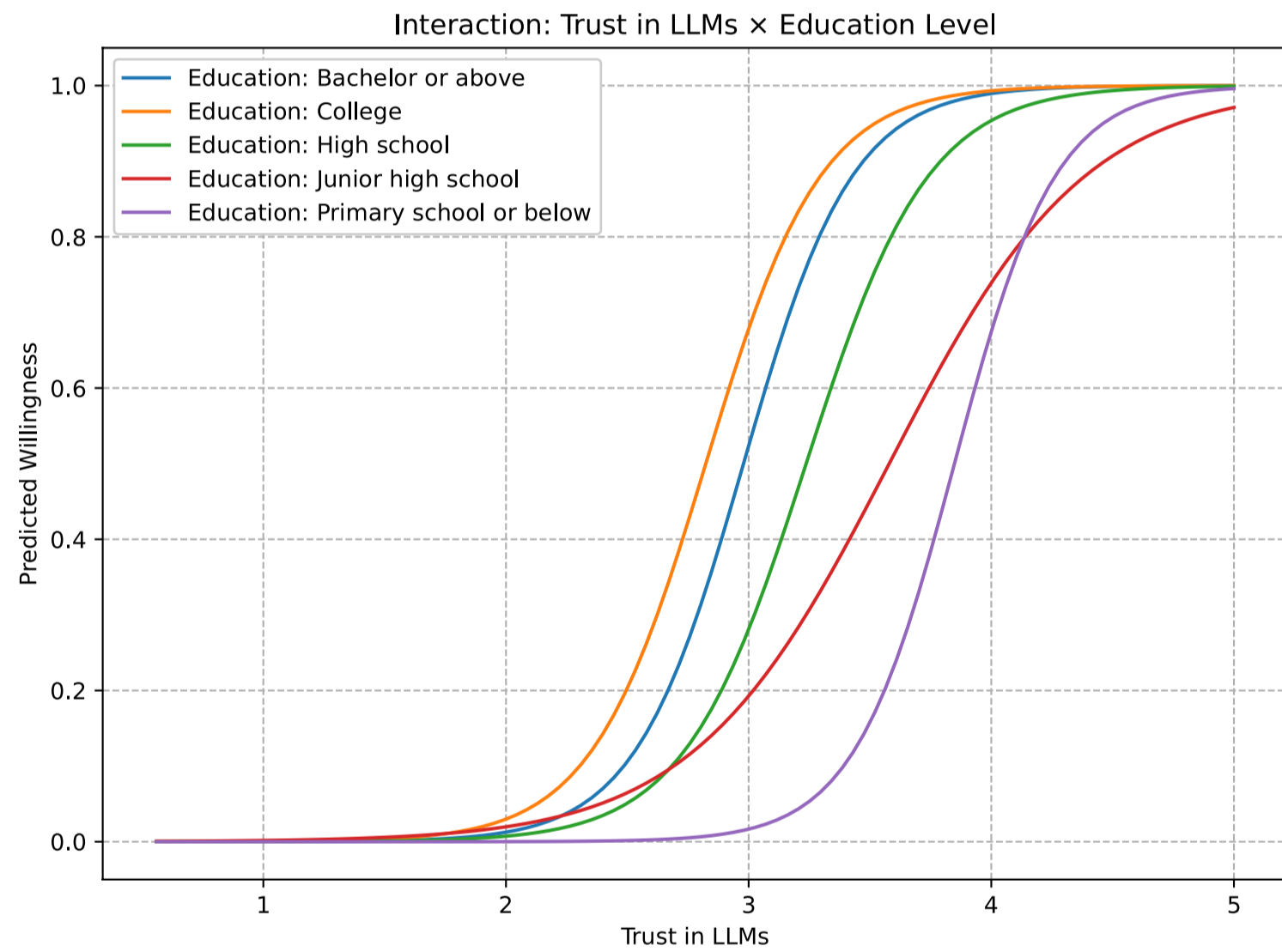**D**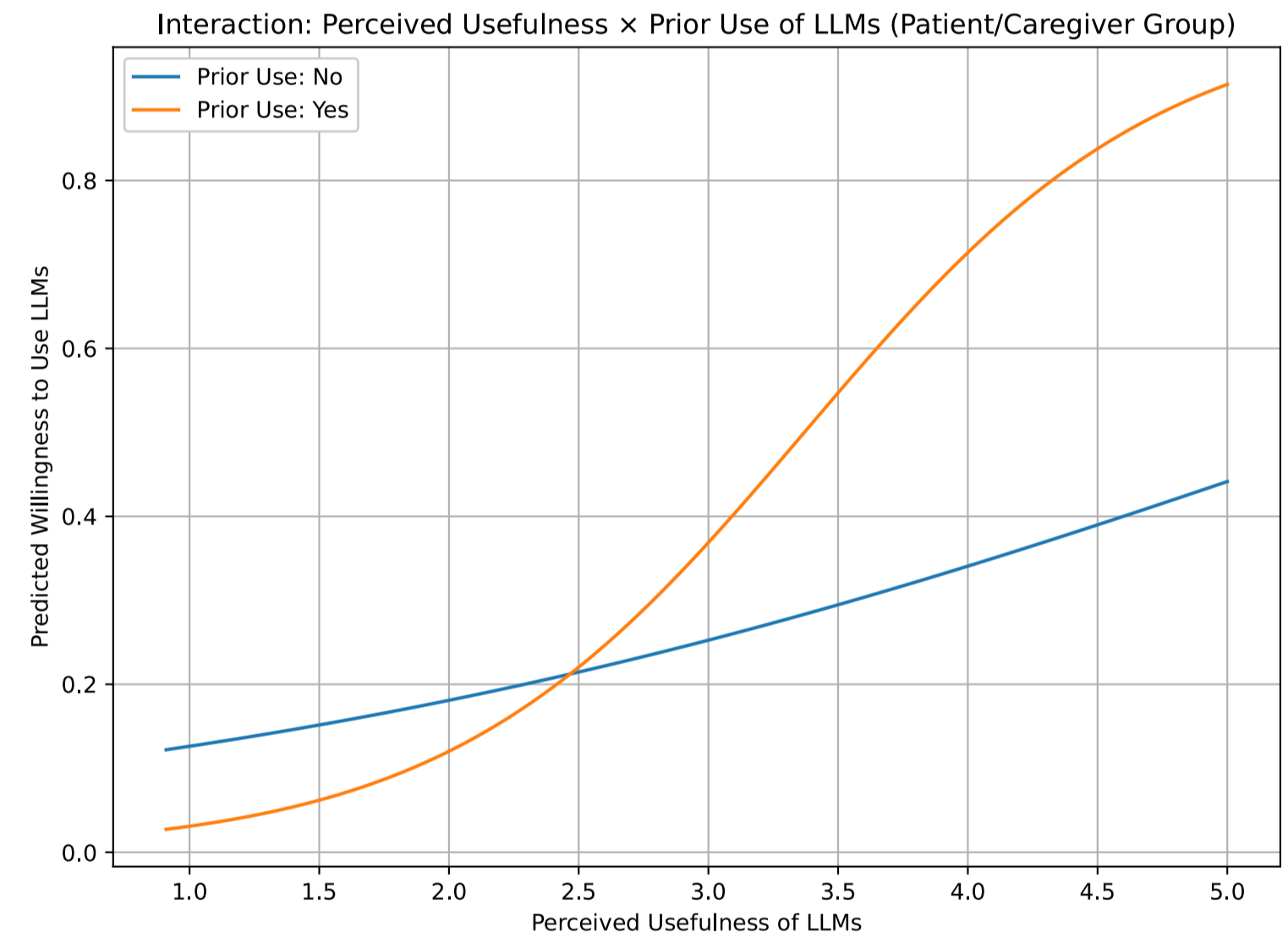**E**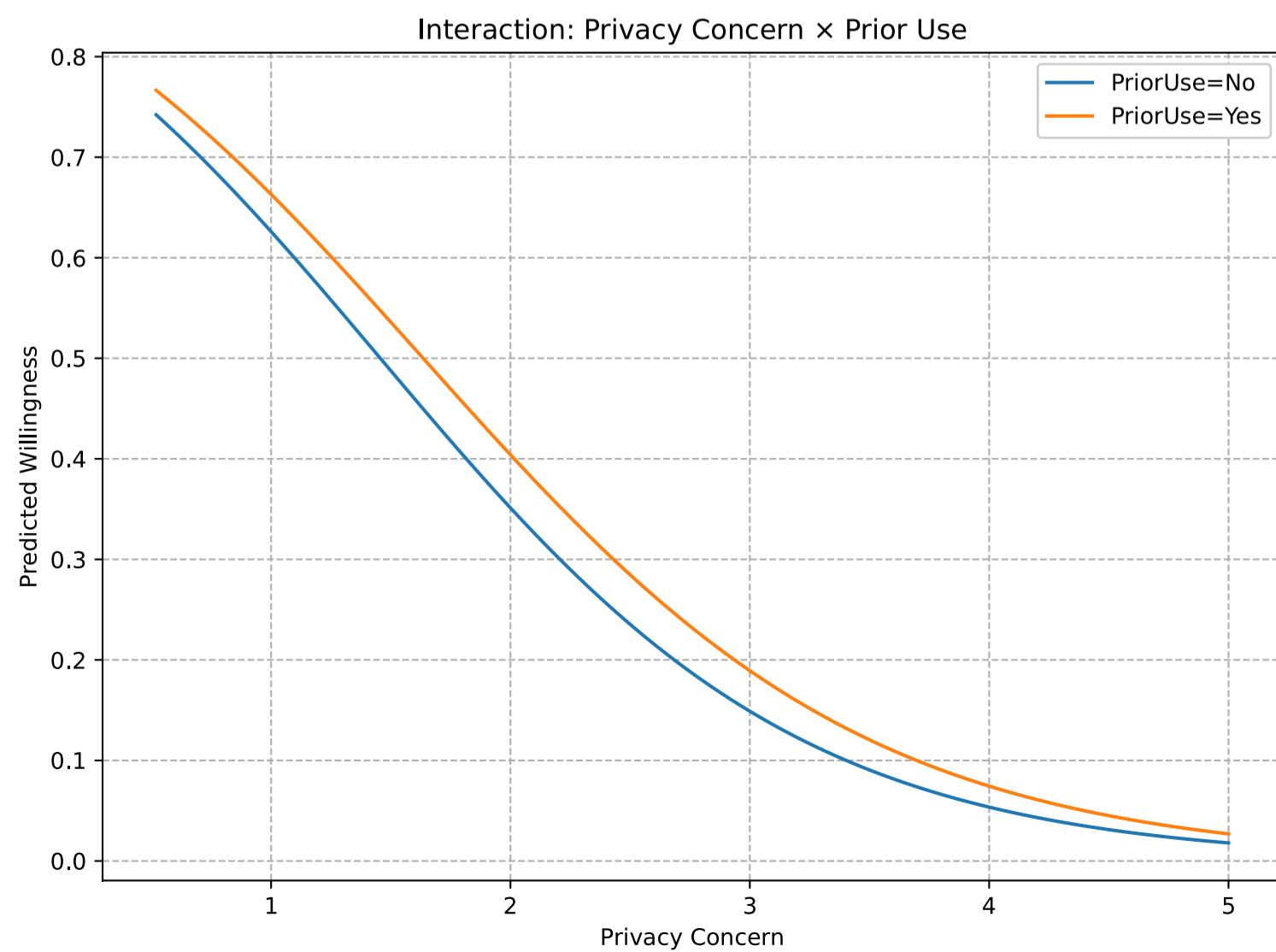**F**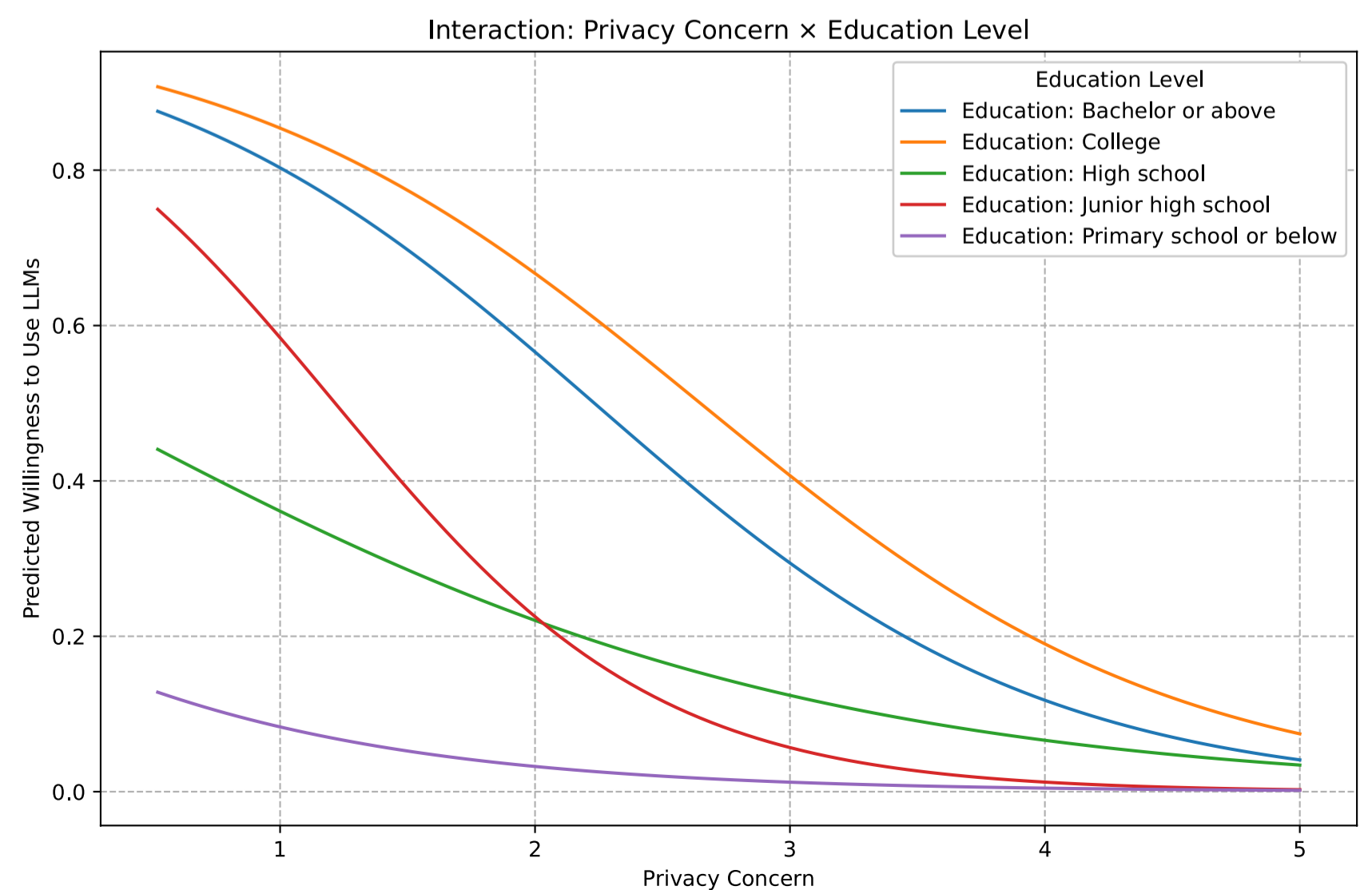

Supplement: Multimedia Appendix 8 [file jmir-v27-e84918-s008.pdf]

A

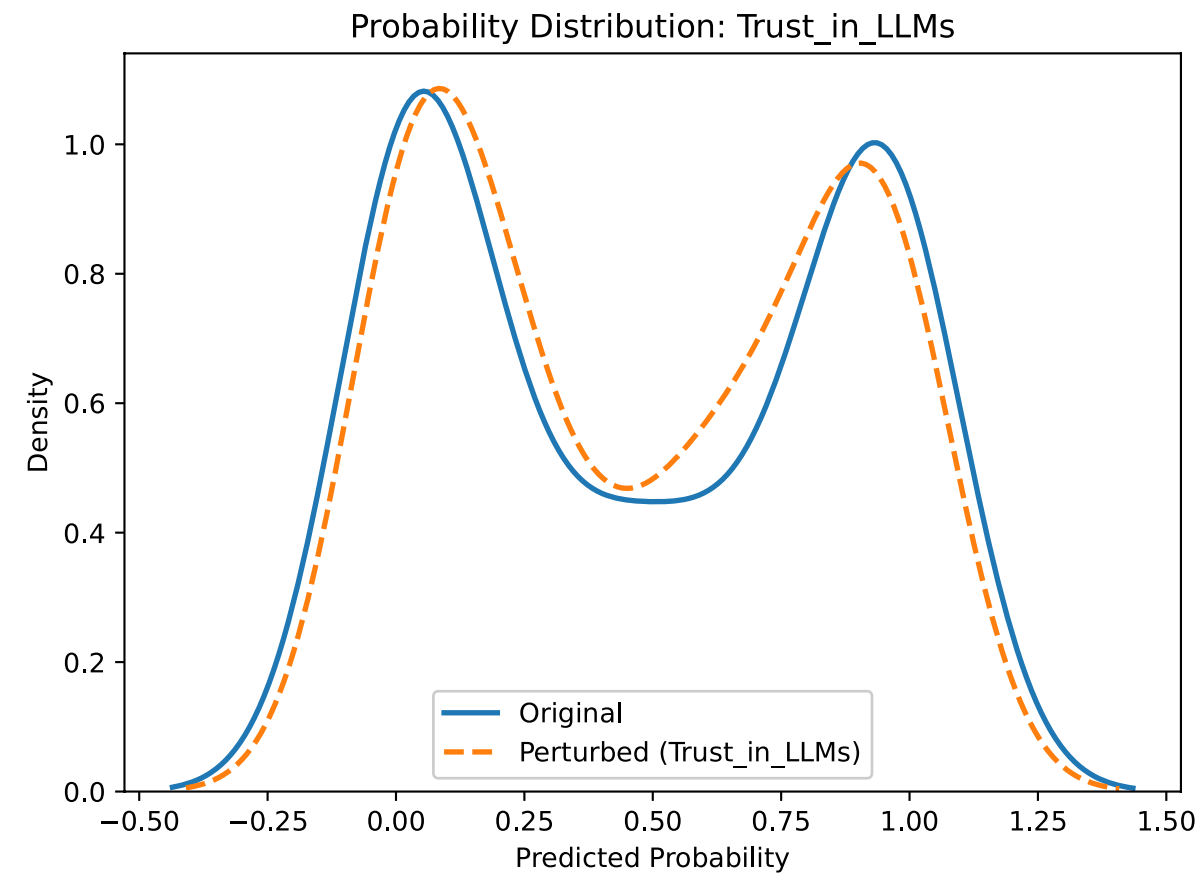

B

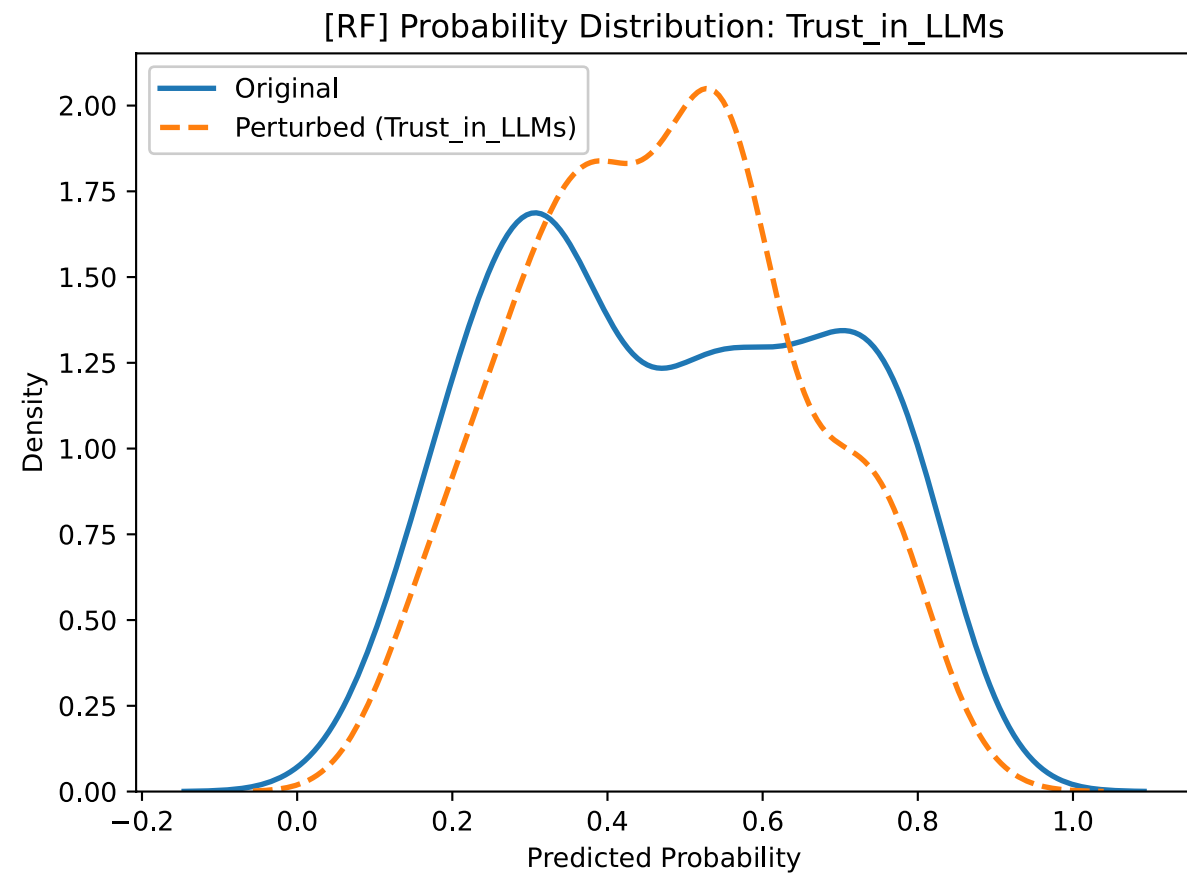

C

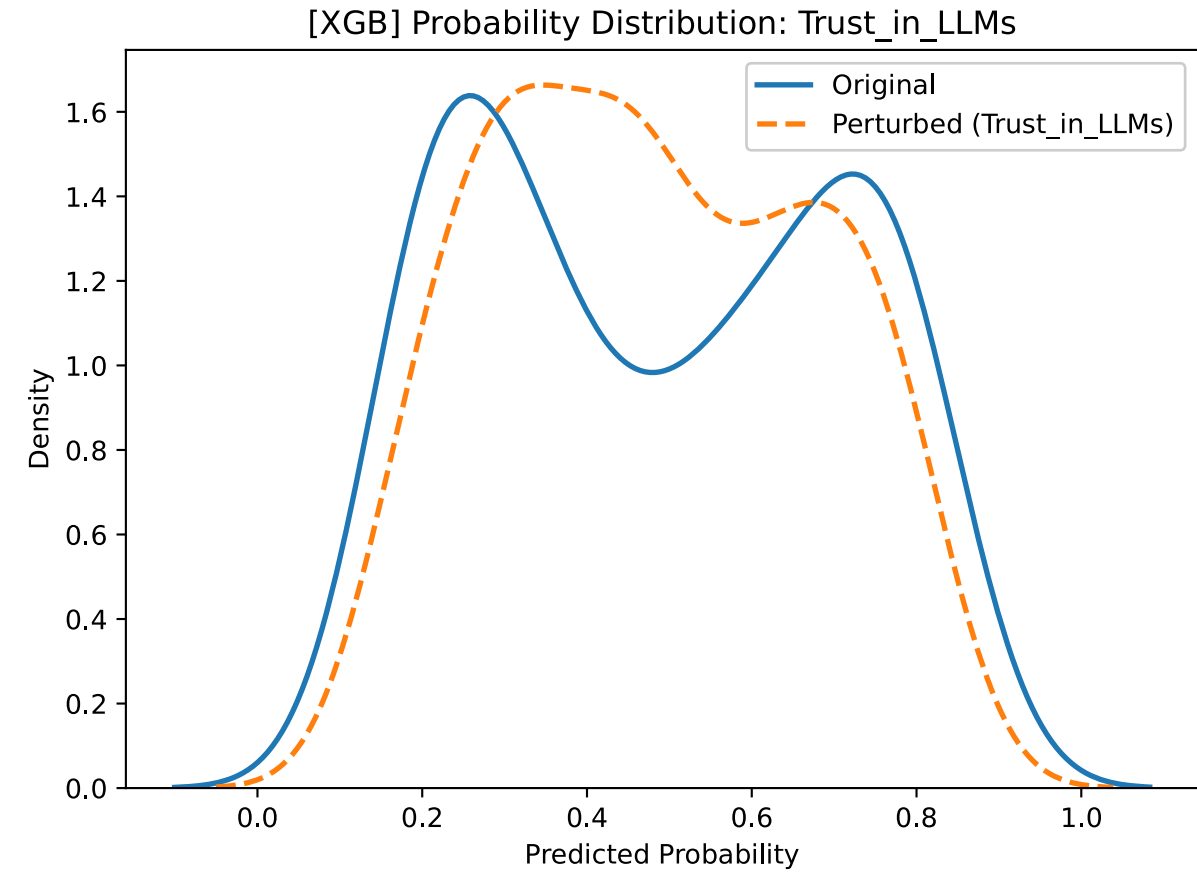

D

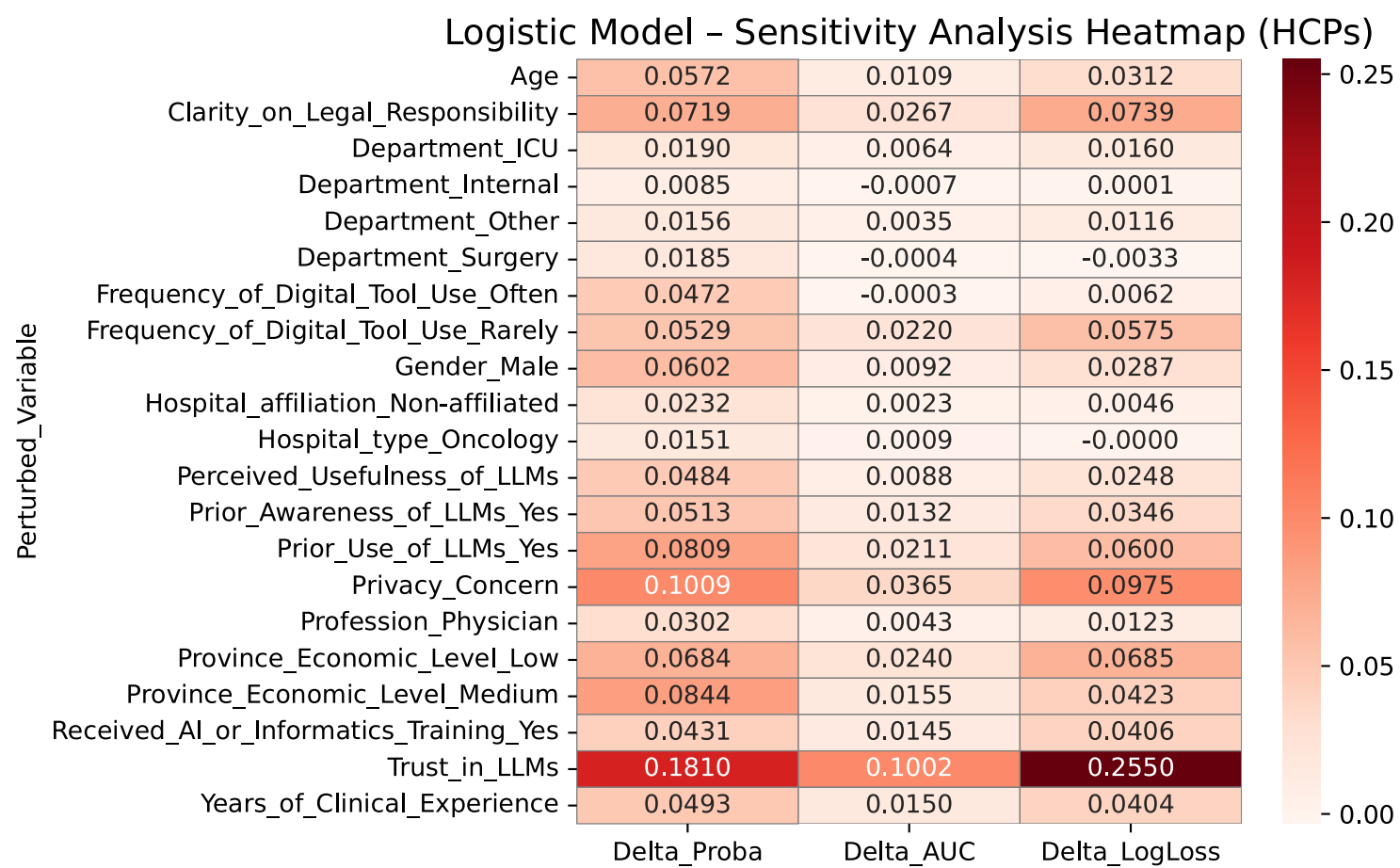

E

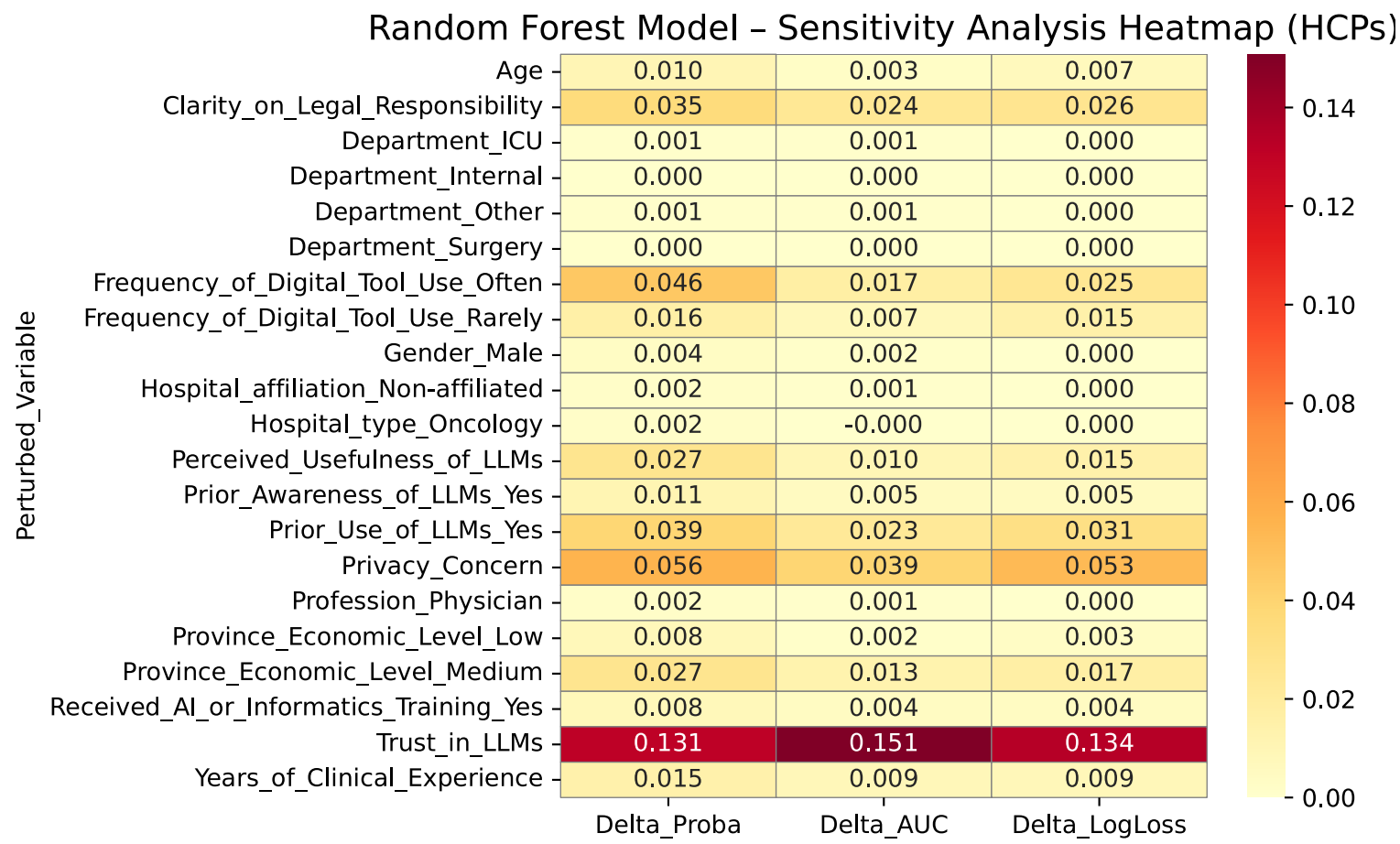

F

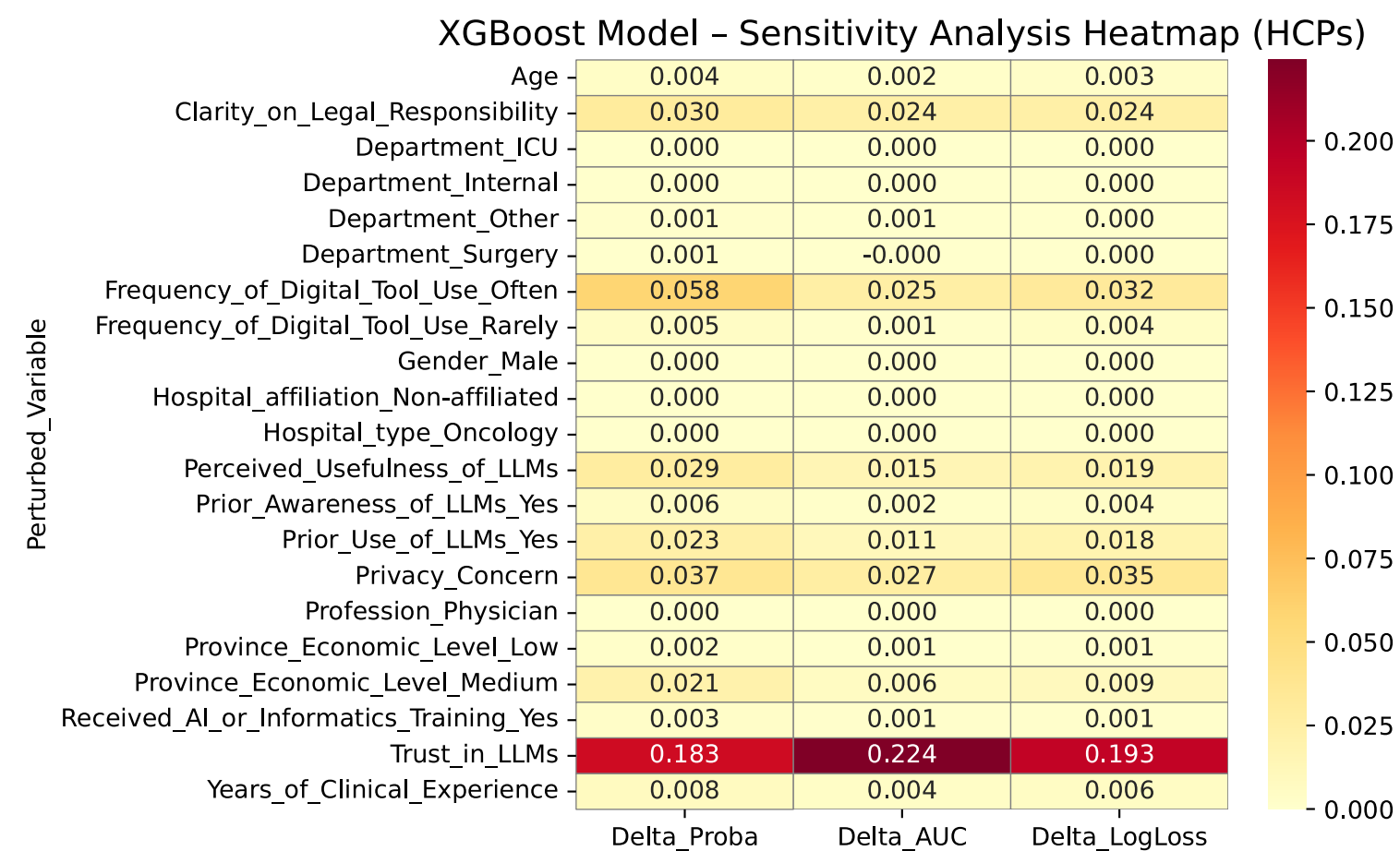

Supplement: Multimedia Appendix 9 [file jmir-v27-e84918-s009.pdf]

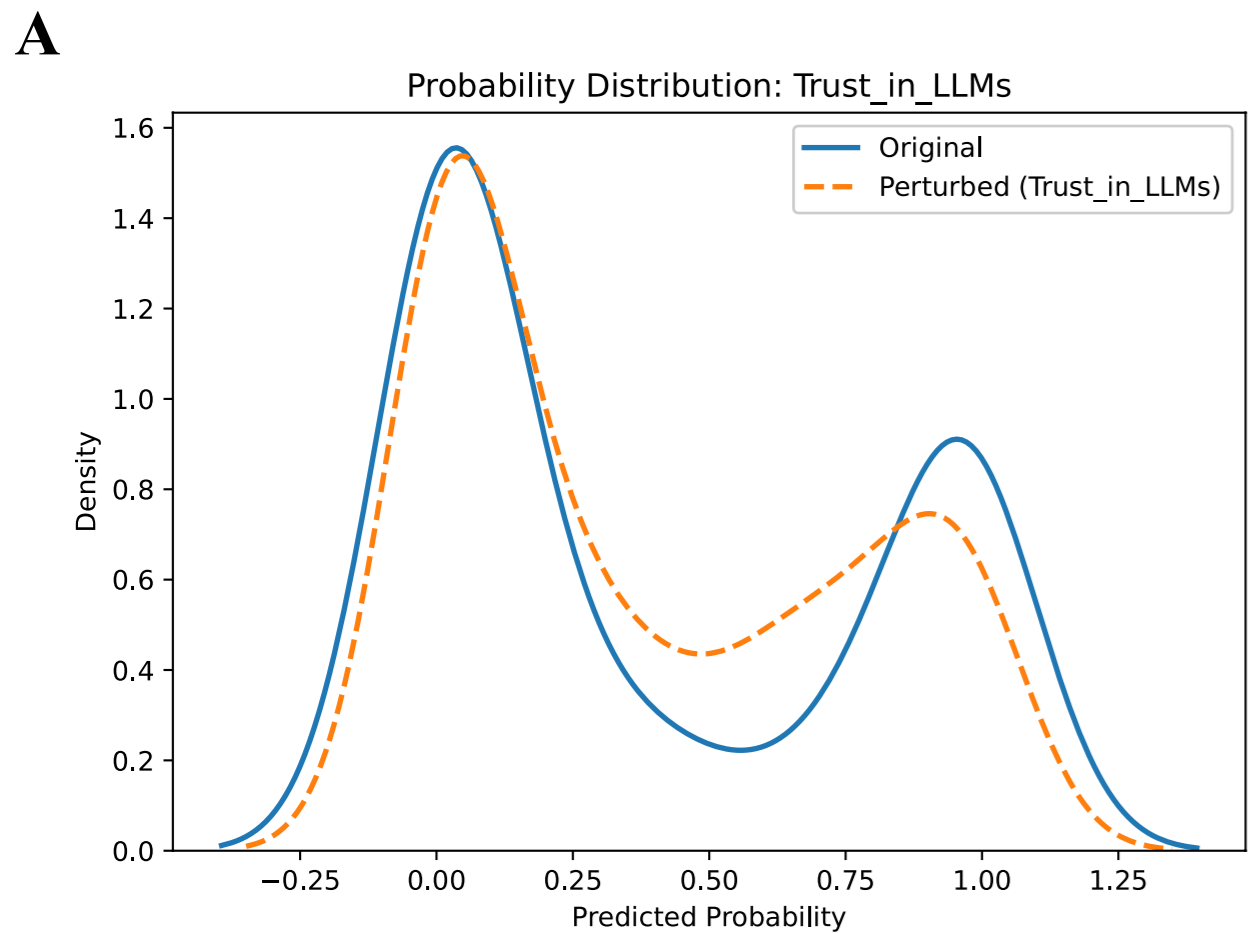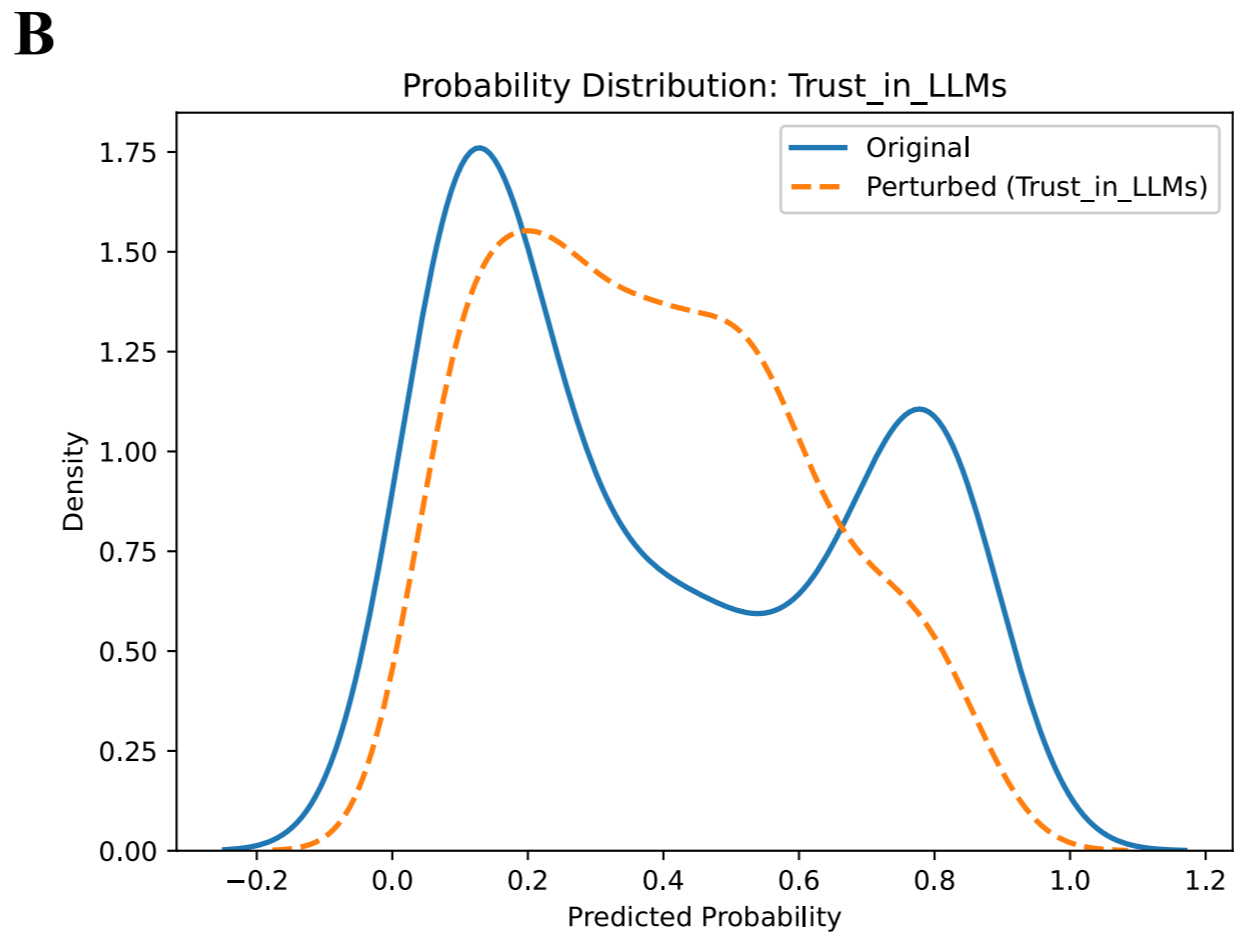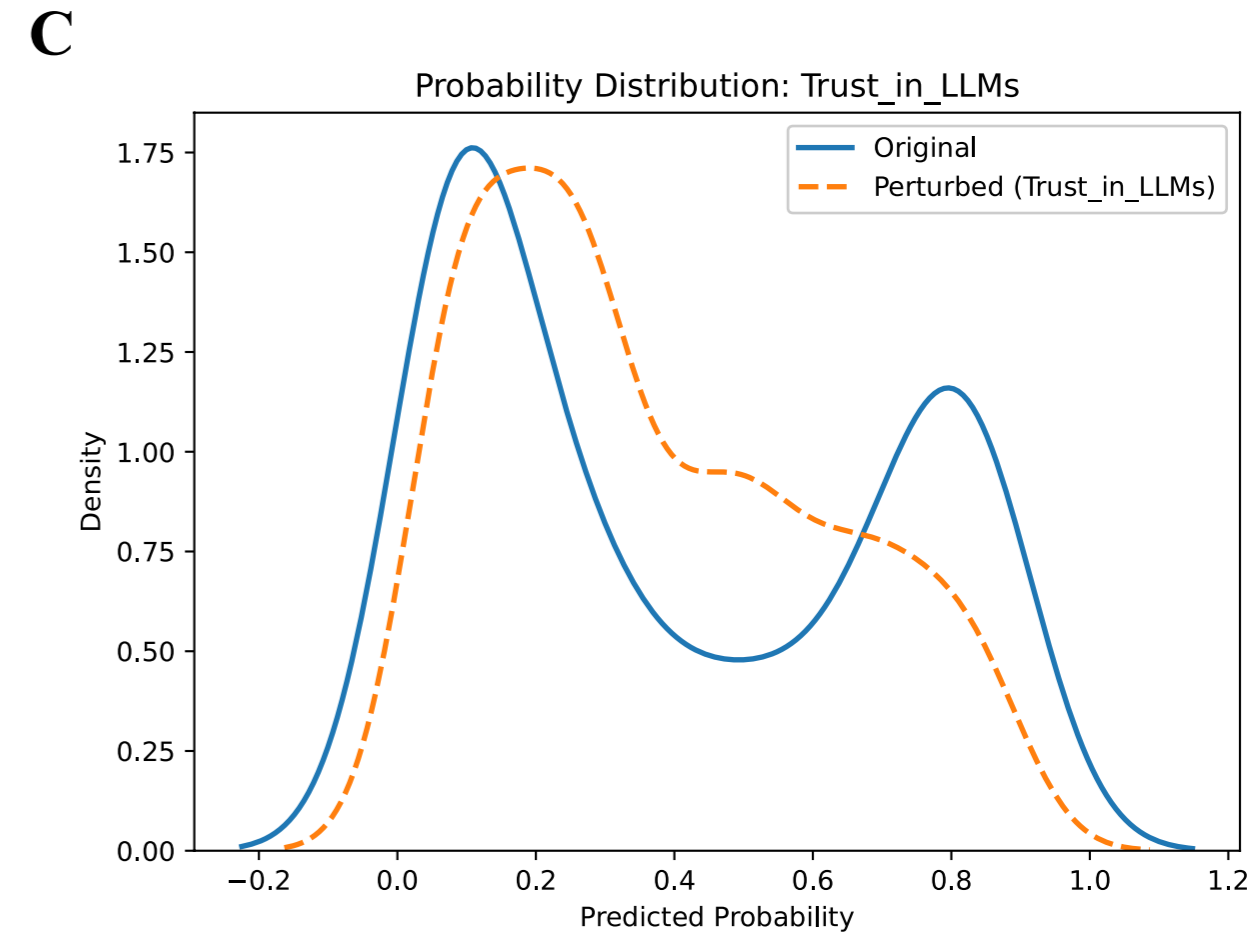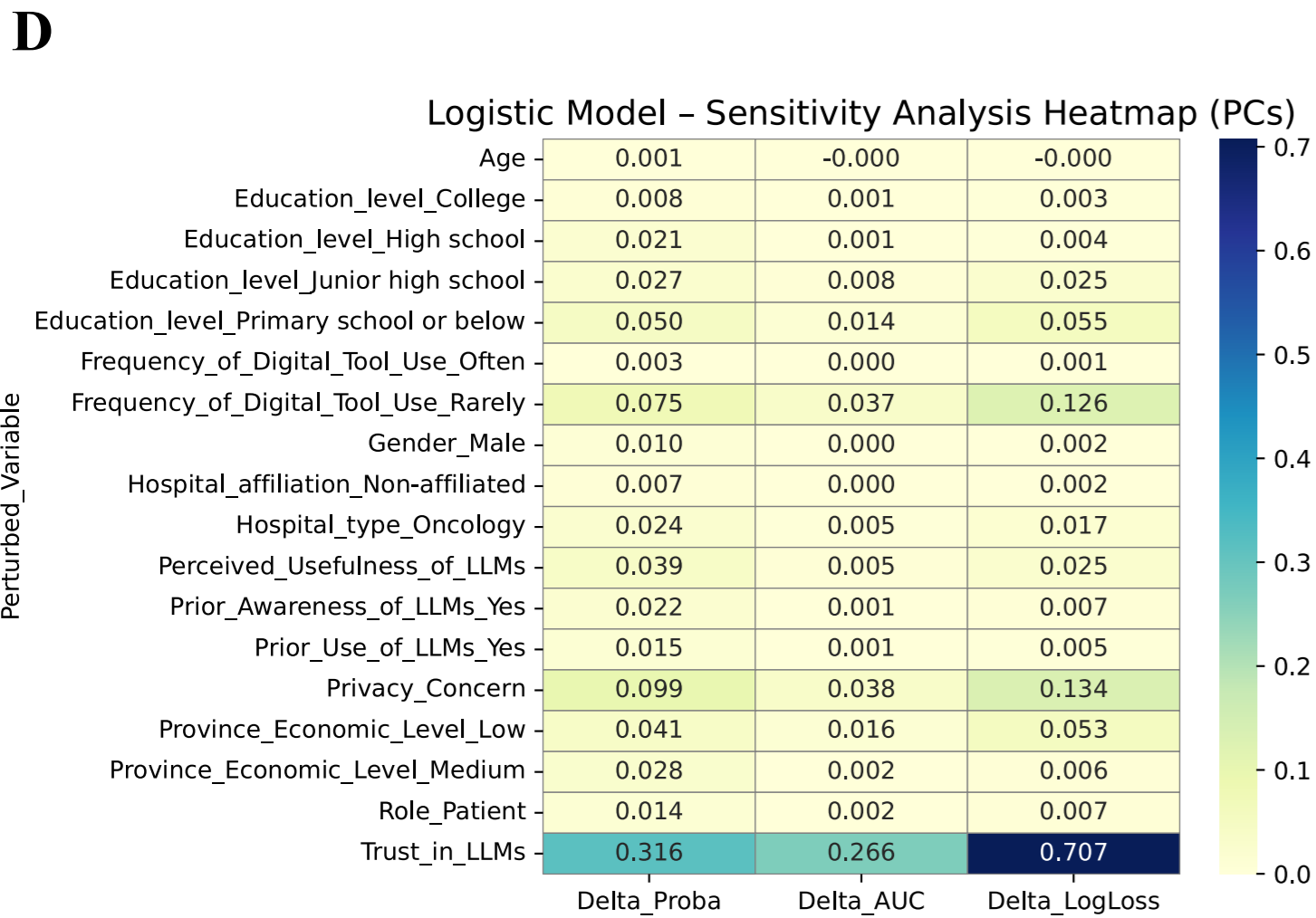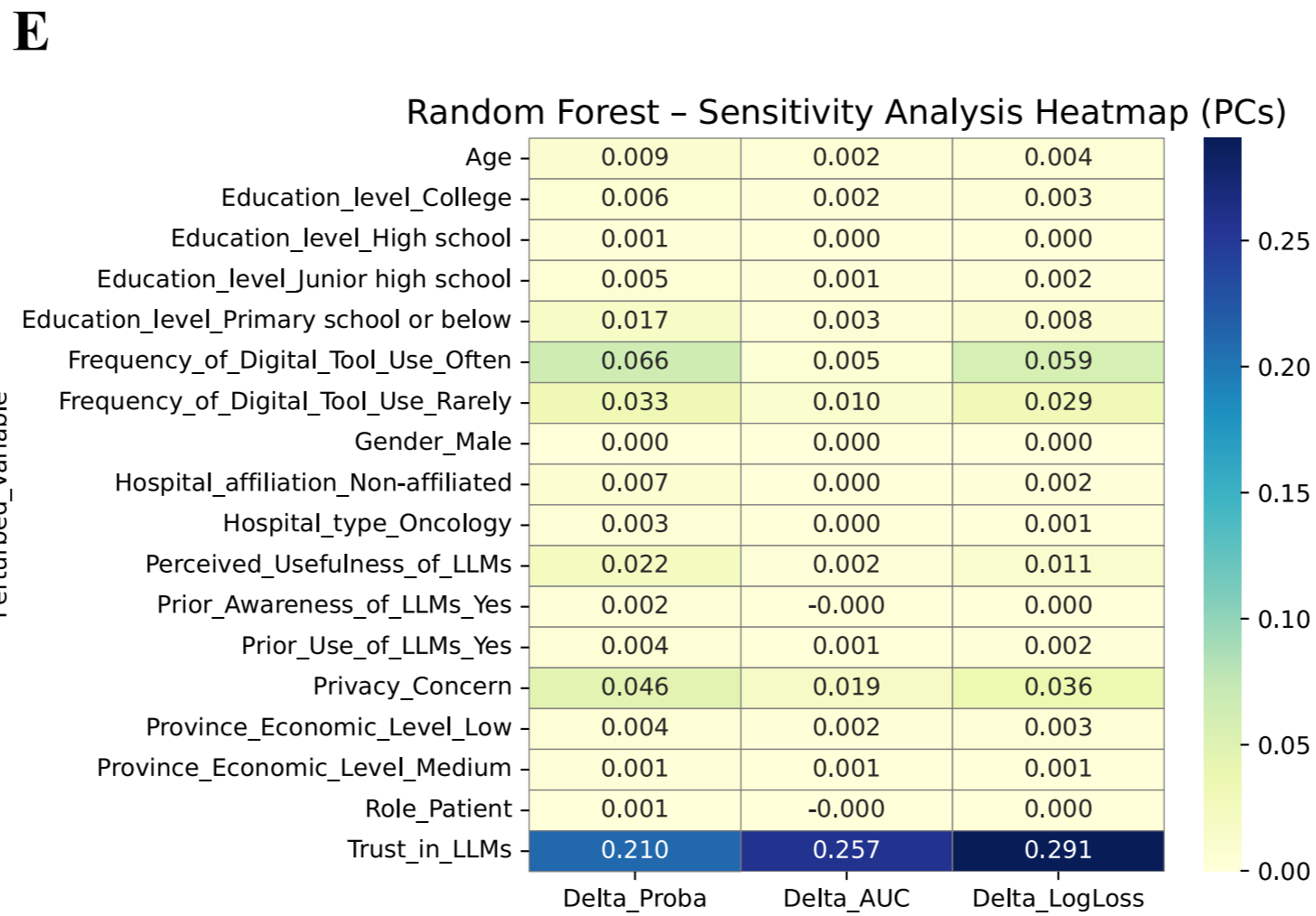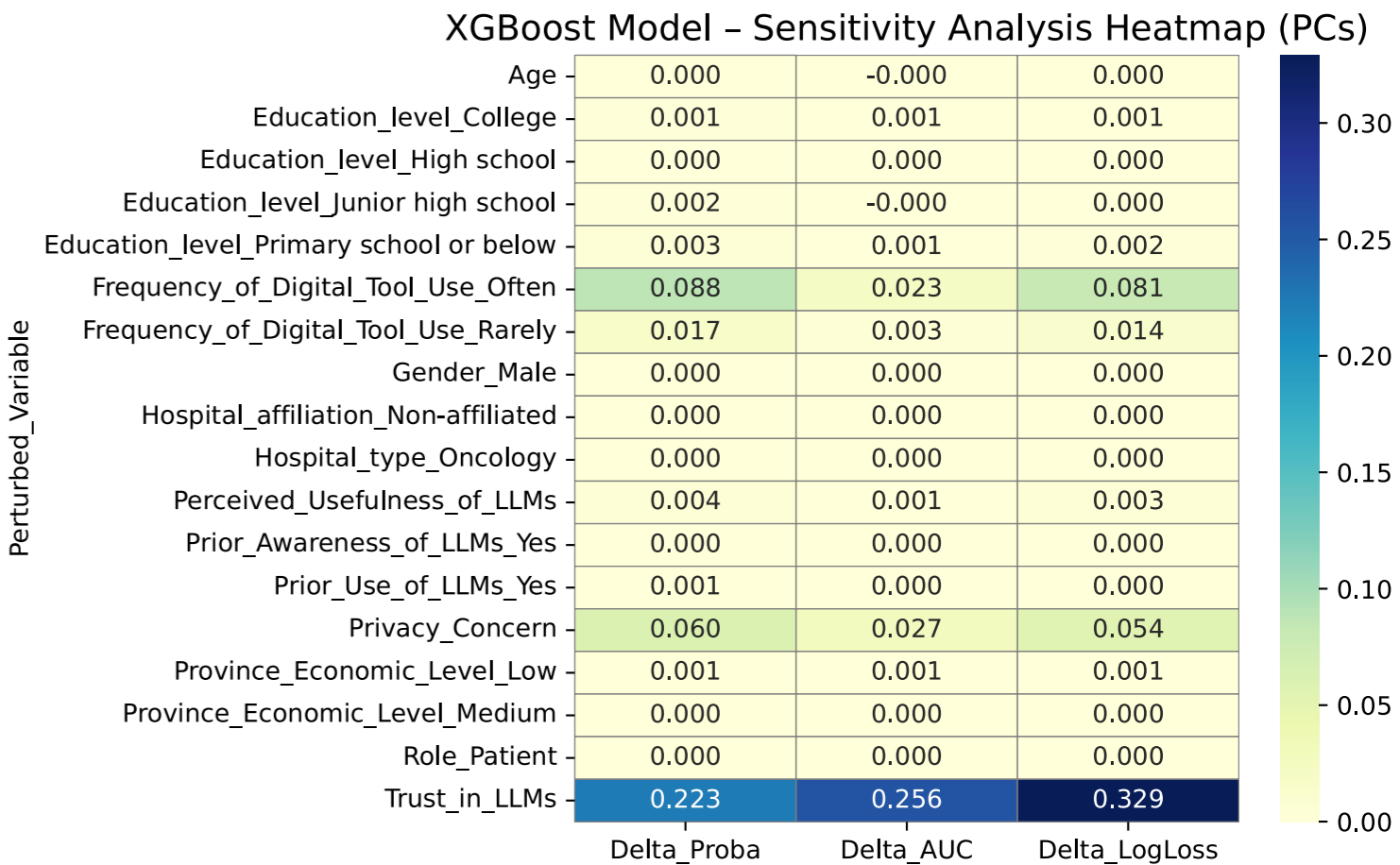

Supplement: Multimedia Appendix 10 [file jmir-v27-e84918-s010.pdf]

A

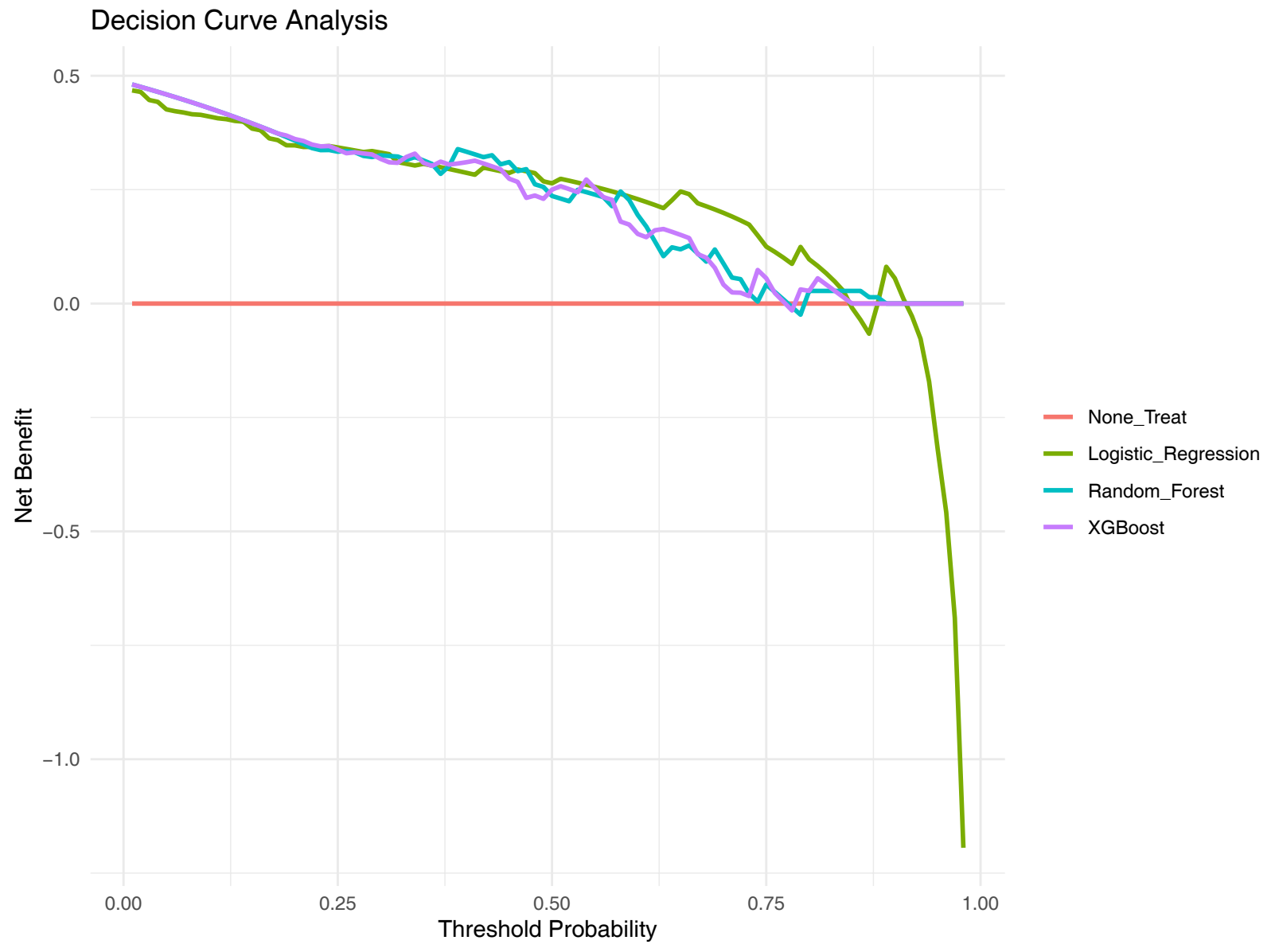

B

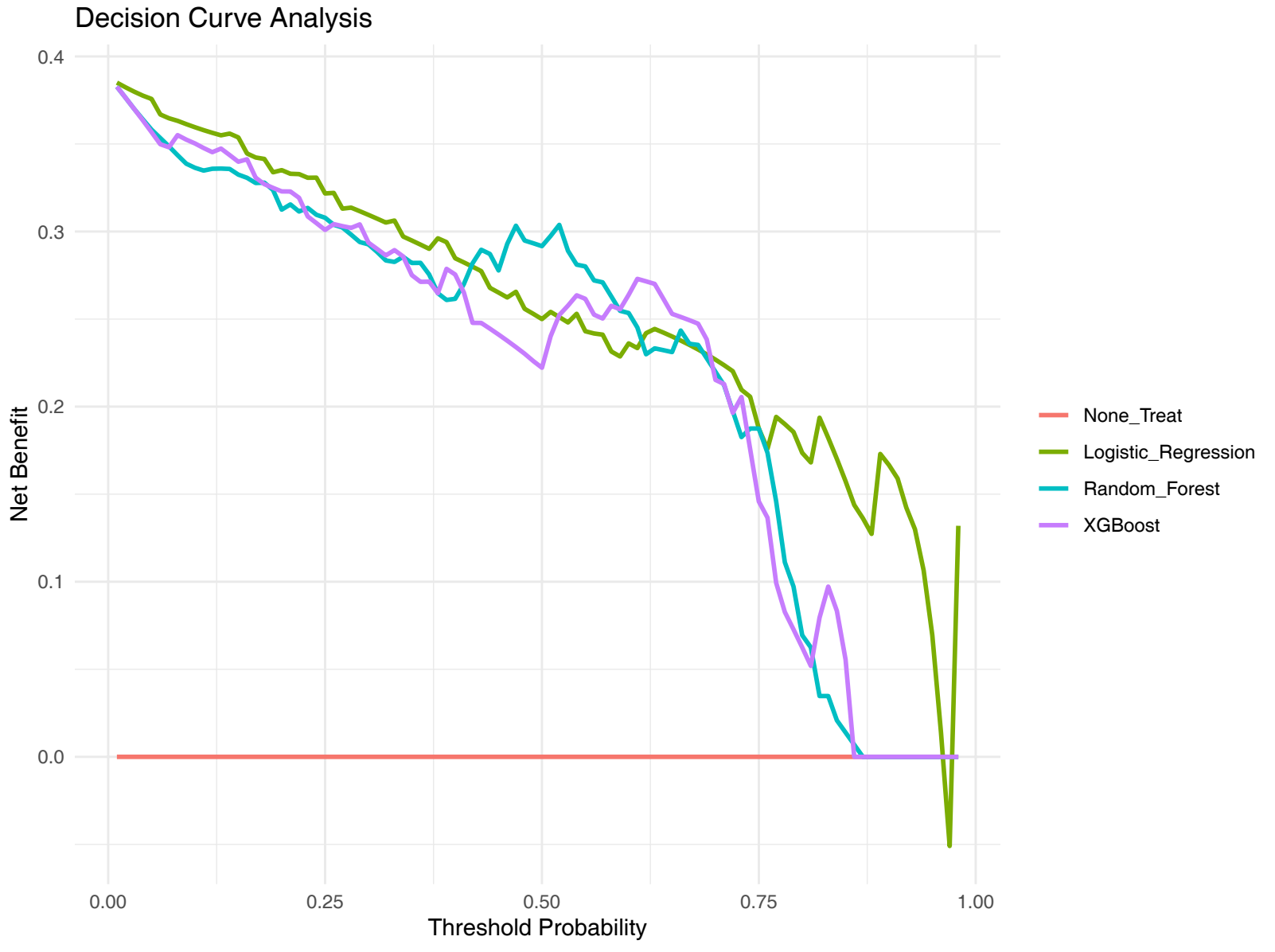

Supplement: Multimedia Appendix 11 [file jmir-v27-e84918-s011.pdf]
