# Supplementary material for: Factors Influencing Adoption of Large Language Models in Health Care: Multicenter Cross-Sectional Mixed Methods Observational Study
Source: J Med Internet Res. 2025 Dec 11;27:e84918. doi: 10.2196/84918 (PMC12697921; doi:10.2196/84918)
Supplement: Multimedia Appendix 12 [file jmir-v27-e84918-s012.pdf]

GPT-5 Interaction Summary

Project Title:

Factors Influencing Adoption of Large Language Models (LLMs) in Health Care: A Multicenter Mixed-Methods Study in China

Time Frame of AI Use:

August 2024 – October 2025

Purpose of AI Assistance:

GPT-5 (OpenAI, San Francisco, CA, USA) was used **exclusively for language refinement and editorial support** during manuscript drafting and revision. Specifically, GPT-5 assisted with:

- English grammar correction and academic style polishing
- Terminology and abbreviation consistency (e.g., standardized use of "healthcare professionals (HCPs)" and "patients/caregivers (PCs)")
- Reformatting of tables, figure captions, and references according to *JMIR* style
- Clarification of reviewer responses and improvement of coherence in *Discussion*, *Limitations*, and *Acknowledgments* sections
- Structural and formatting improvements to meet journal submission requirements

Scope Explicitly Excluded from AI Use:

GPT-5 was **not used** for any scientific or intellectual work, including:

- Study design, hypothesis formulation, or data collection
- Quantitative or qualitative data analysis and interpretation
- Generation, modification, or verification of results or references
- Conceptual framing, theoretical interpretation, or conclusion development

Extent and Oversight:

AI assistance was iterative and limited to text editing. **All AI-suggested edits were reviewed, verified, and approved by the human authors** before inclusion in the manuscript. Final intellectual content, data integrity, and interpretation remain entirely the responsibility of the authors.

Representative Examples of AI Use:

1. Grammar and syntax refinement of the *Discussion* section (e.g., "LLMs' use might impact communication efficiency" → "LLMs may improve communication efficiency between patients and providers").
2. Harmonization of variable labels, statistical notation (e.g., replacing "p < .05" with "P < .05"), and reference formatting.
3. Language and structure refinement for reviewer response letters and the *Acknowledgments* section.

Record Retention:

Due to the extended project duration, some early GPT-5 interaction records were not retained. However, this summary accurately documents the scope, timing, and supervision of AI use in accordance with *JMIR Publications' Policy on the Responsible Use of Generative AI (2024)*.

Prepared by:

Chuan Xu, Email: xuchuan89757@163.com; Telephone Number:13985520269; Postal Code: 550000; 1. Department of Thoracic Surgery, Guizhou Provincial People's Hospital, No. 83, Zhongshan East Road, Guiyang, Guizhou, China. 2. NHC Key Laboratory of Pulmonary Immunological Diseases, Guizhou Provincial People's Hospital, Guiyang, Guizhou, China.

Xiongwen Yang, Email: yangxiongwen@gz5055.com; Telephone Number: 15673740612; Postal Code:

550000; 1. Department of Thoracic Surgery, Guizhou Provincial People's Hospital, No. 83, Zhongshan East Road, Guiyang, Guizhou, China. 2. NHC Key Laboratory of Pulmonary Immunological Diseases, Guizhou Provincial People's Hospital, Guiyang, Guizhou, China.

Date: October 12, 2025
